# Clonal isolates of *Treponema pallidum* subsp. *pallidum* Nichols provide evidence for the occurrence of microevolution during experimental rabbit infection and *in vitro* culture

**DOI:** 10.1371/journal.pone.0281187

**Published:** 2023-03-14

**Authors:** Diane G. Edmondson, Bridget D. De Lay, Blake M. Hanson, Lindsay E. Kowis, Steven J. Norris

**Affiliations:** 1 Department of Pathology & Laboratory Medicine, McGovern Medical School, University of Texas Health Science Center, Houston, Texas, United States of America; 2 Department of Pediatrics, McGovern Medical School, University of Texas Health Science Center, Houston, Texas, United States of America; 3 Department of Epidemiology, Human Genetics & Environmental Sciences, Center for Infectious Diseases, School of Public Health, University of Texas Health Science Center, Houston, Texas, United States of America; 4 Houston Methodist Research Institute, Infectious Disease, Houston, Texas, United States of America; 5 Department of Microbiology & Molecular Genetics, McGovern Medical School, University of Texas Health Science Center, Houston, Texas, United States of America; University of Kentucky College of Medicine, UNITED STATES

## Abstract

The recent development of a system for long-term *in vitro* culture of the syphilis spirochete, *Treponema pallidum* subsp. *pallidum*, has introduced the possibility of detailed genetic analysis of this bacterium. In this study, the *in vitro* culture system was used to isolate and characterize clonal populations of *T*. *pallidum* subsp. *pallidum* Nichols, the most widely studied strain. In limiting dilutions experiments, it was possible to establish cultures with inocula as low as 0.5 *T*. *pallidum* per well despite the long generation time (~35 to 40 hours) of this organism. Six Nichols strain clones isolated by limiting dilution were characterized in detail. All clones exhibited indistinguishable morphology and motility, highly similar *in vitro* multiplication rates, and comparable infectivity in the rabbit model (ID50 ≤ 100 bacteria). Genomic sequencing revealed sequence heterogeneity in the form of insertions or deletions at 5 sites, single nucleotide variations at 20 sites, and polynucleotide (polyG/C) tract length differences at 22 locations. Genomic sequences of the uncloned Nichols strain preparations propagated in rabbits or *in vitro* cultures exhibited substantial heterogeneity at these locations, indicating coexistence of many varied ‘clonotypes’ within these populations. Nearly all genetic variations were specific for the Nichols strain and were not detected in the >280 *T*. *pallidum* genomic sequences that are currently available. We hypothesize that these Nichols strain-specific sequence variations arose independently either during human infection or within the 110 years since the strain’s initial isolation, and thus represent examples of microevolution and divergence.

## Introduction

*Treponema pallidum* is a monophyletic group of pathogens that cause syphilis (subsp. *pallidum*), bejel (also called endemic syphilis; subsp. *endemicum*), and yaws in humans and similar diseases in other primates (subsp. *pertenue*) [1–6]. *Treponema paraluiscuniculi*, the causative agent of sexually transmitted spirochetosis in rabbits and hares, is also a closely related member of this group. Members of this “*T*. *pallidum* group” are obligate pathogens with absolute dependence on their mammalian hosts in nature. They are deficient in many biosynthetic activities and must acquire a wide variety of nutrients from the host, including amino acids, nucleic acid precursors, and fatty acids [4,7,8]. The spirochetes are transmitted by contact of lesion exudates with epidermal or mucosal surfaces (and by transplacental passage in congenital syphilis); they give rise to local lesions and often disseminate to distant tissues. These infections may last and cause manifestations for years to decades, reflecting the expression of immune evasion mechanisms, including the paucity of surface proteins and antigenic variation of the protein TprK through a gene conversion mechanism [9–14].

The *T*. *pallidum* group of spirochetes have a genome consisting of a small, circular chromosome (~1.13 to 1.14 Mb), share extensive gene synteny, and have an overall genome sequence identity of 99.16 to 99.7 percent and a G+C content of approximately 52.4% [15]. In comparison, *Treponema phagedenis*, the species considered to be the closest relative outside of this group, has a 2.8 to 3.7 Mb circular genome with ~39.5% G+C [16]; it also shares only limited regions of gene synteny or high sequence identity with *T*. *pallidum*. Thus the *T*. *pallidum* group appears to have undergone extensive evolution and genome reduction to become highly specialized, host dependent, invasive, and persistent infectious agents; the minor divergence within this group appears to be a relatively recent event that has led to different mammalian host ranges and patterns of pathogenesis. The limited sequence differences among *T*. *pallidum* subsp. *pallidum* strains indicate divergence into two major clusters; these clusters are commonly called “Nichols-like” and “SS14-like” based on their genetic similarities to these two well-characterized strains [17]. The patterns of inter- and intra-species divergence have been reinforced by several studies in which the number of available genome sequences have been expanded greatly through direct sequencing of specimens from infected humans and primates [18–23]. This divergence has been ongoing for centuries based on genomic sequencing results obtained from ancient DNA specimens [24]. There is also evidence for genetic exchange and recombination events between *T*. *pallidum* subspecies and lineages, adding another layer of complexity [21,22].

Until recently, *T*. *pallidum* could not be maintained by *in vitro* culture, so it was necessary to propagate the bacteria through the inoculation of rabbits or other mammalian hosts [25,26]. Long-term *in vitro* culture of *T*. *pallidum* subsp. *pallidum* and subsp. *endemicum* strains was first reported in 2018 [27]. The *T*. *pallidum* culture system includes co-culture with Sf1Ep cottontail rabbit epithelial cells, a modified tissue culture medium called *T*. *pallidum* Culture Medium 2 (TpCM-2), and incubation at 34°C in a microaerobic atmosphere [27–31]. Under these conditions, the spirochete retains its morphology, motility, and infectivity and multiplies with minimum generation times ranging from 30 to 44 h [27,30]; these values are similar to estimated generation times of 32 to 35 h obtained during experimental rabbit infections [32,33]. *In vitro* cultures of several strains of *T*. *pallidum* subsp. *pallidum* and *endemicum* have now been established [30]. To date, attempts to propagate *T*. *pallidum* subsp. *pertenue* and *T*. *paraluiscuniculi in vitro* have been unsuccessful [30].

The availability of an *in vitro* culture system for *T*. *pallidum* strains provides an avenue for more detailed genetic studies such as mutational analysis, as recently demonstrated by Romeis et al. [34]. Genetic analyses are best performed with homogeneous populations, which in bacterial studies are represented by clones derived from single organisms. The resulting isogenic cultures are key to molecular genetic experiments, in that the phenotypic effects of a mutation in an isogenic clone can be more readily and directly attributed to the mutation as opposed to other genetic differences existing in the strain. Further confirmation is of course provided by complementation of the mutation and restoration of the parental phenotype. These principles are also central aspects of the “molecular Koch’s postulates” described by Dr. Stanley Falkow [35], in which he proposed how the role of a gene in pathogenesis should be determined. Clones of *T*. *pallidum* subsp. *pallidum* Nichols strain had been isolated previously using IV inoculation of rabbits and isolation of organisms from well separated skin lesions (see GenBank entries CP010560.1 and CP010561.1 by Iverson-Cabral and Giacani as examples). However, such lesions take 2–3 weeks to develop. During that time, the rabbits’ immune responses apparently selected for variants in the *tprK* immune evasion system [9], so that each of the seven *tprK* variable regions contained a heterogeneous mixture of sequences.

In this study, we utilized the *T*. *pallidum in vitro* culture system to isolate clones from *T*. *pallidum* Nichols and other strains using a limiting dilution method. The results indicate the feasibility of this approach and provide insights into the occurrence of microevolution and divergence in *T*. *pallidum*.

## Results

### Continuous culture of *T*. *pallidum*

Two independent cultures of *T*. *pallidum* subsp. *pallidum* Nichols (hereafter called *T*. *pallidum* Nichols) have now been maintained by serial subculture for over 4.5 years; in this article, the two ‘lineages’ (initiated on Oct. 20, 2017 and Nov. 3, 2017, respectively) are herein referred to as *In vitro* A (IVA) and *In vitro* B (IVB) cultures. The cultures are remarkably stable and represent 1,014 and 1,078 generations of *in vitro* growth, respectively. Motility of *T*. *pallidum* has been sustained at high levels throughout most of the culture period and infectivity has been maintained with fewer than 10 organisms required to result in lesion formation in rabbits [30]. Generation time averaged 41 and 39 hours respectively (range 31–71 hours).

### Generation of clonal isolates of *T*. *pallidum*

To determine if we could generate clonal isolates of *T*. *pallidum*, we first examined the lower limits of *T*. *pallidum* cloning efficiency using limiting dilution. In two replicate experiments, actively growing cultures were diluted serially (1:5) and seeded into prepared 96-well plates. Sixteen wells were plated at each density ranging from 50,000–0.001 *T*. *pallidum* (Nichols strain) per well. Half of the ‘spent’ medium (100 μl) was removed from each well and replaced with fresh TpCM2 at 7-d intervals. The culture supernatants were examined weekly by darkfield microscopy for the presence of *T*. *pallidum*; in some cases, quantitative PCR was also performed to provide another means of *T*. *pallidum* detection.

**Fig 1:**
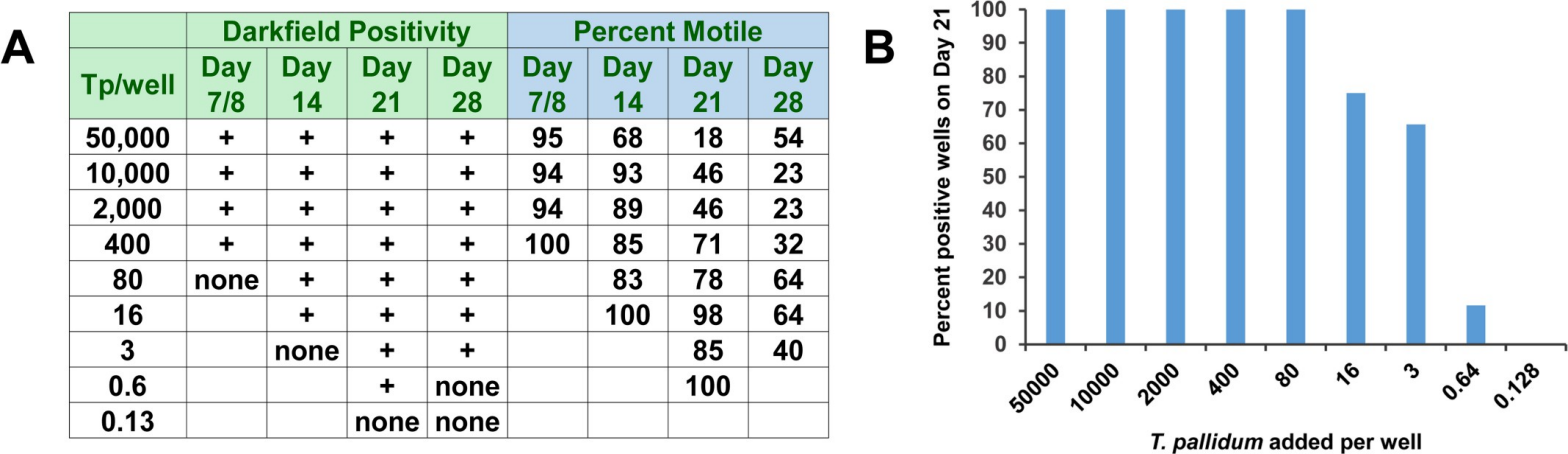
Culture of *T*. *pallidum* from low inocula. *T*. *pallidum* Nichols was inoculated at the indicated concentrations into 96-well plates in co-cultures with Sf1Ep cells and incubated in TpCM-2 under standard *T*. *pallidum in vitro* culture conditions. Half of the medium was replaced with fresh TpCM-2 weekly. (A) *T*. *pallidum* culture positivity and motility at each inoculum concentration, as determined by darkfield microscopy at each time point. (B) Percentage of wells positive for *T*. *pallidum* at 21 days post inoculation.

The combined results of these two experiments are shown in Fig 1. *T*. *pallidum* were observed at inoculation densities as low as 0.6 organisms per well. Motility of *T*. *pallidum* remained high in low-density cultures for up to three weeks but was lost as the Sf1Ep cell layer failed. All cultures had poor motility after 4 weeks. In wells seeded at higher densities (400–50,000 *T*. *pallidum* per well) spirochetes reached densities of ~ 1.0 x 10^7^/ml; however wells seeded at low densities rarely exceeded 2 x 10^6^/ml before failure of the culture. The 14- to 21- day interval required to obtain numbers of *T*. *pallidum* that can be detected by microscopy (>10^4^/ml) is consistent with the ~40-h doubling time for this organism. The average cloning efficiency (percent of wells positive per organism inoculated) at 0.6 *T*. *pallidum* per well was 12.0%, with values of 26.6% and 9.4% in each of the two experiments; this value is thus variable, most likely because of differing phases of growth (e.g. late log vs. stationary) or other properties of the inoculating culture.

Based on these preliminary studies, 96-well plates were inoculated with *T*. *pallidum* Nichols from culture IVB on day 328 of *in vitro* culture. One plate each was inoculated at a calculated density of 2 and 0.5 *T*. *pallidum* per well and incubated under the conditions described above. In this experiment, 100 μl of each culture supernatant were transferred to fresh cultures at 14 days, due to the limited lifespan of Sf1Ep cells under these conditions. The cloning methodology is described in greater detail in another article [31].

*T*. *pallidum* were detected by microscopy in 24 of 96 wells in the 2 *T*. *pallidum*/well plate and 2 of 96 wells in the 0.5 *T*. *pallidum*/well plate, representing cloning efficiencies of 12.5% and 6.7%, respectively. To screen the recovered isolates for clonality, we performed PCR of *TP0222* and *TP0488*, genes known to have polymorphisms in the Nichols strain of *T*. *pallidum* (at nucleotides 228259 and 523669 in the reference sequence NC_021490.2, respectively). Sanger sequencing of the PCR products and examination of the resulting chromatographs demonstrated that 24 of 26 potential clones had a single nucleotide (either A or C) at each of these positions, whereas the rabbit-propagated Nichols strain control had a mixture of the two nucleotides at these locations.

Putative clones TpN-CL1, TpN-CL3, TpN-CL4, TpN-CL2, TpN-CL5, and TpN-CL8 were homogeneous at these sites and were chosen for further characterization. TpN-CL1, TpN-CL3, and TpN-CL4 had A at both the *TP0222* and *TP0488* sites, whereas TpN-CL2, TpN-CL5, and TpN-CL8 had C at both sites; these differences provided an early indication of the presence of genetic similarities and differences among the putative clones.

### Growth rates and infectivity of clones

**Fig 2:**
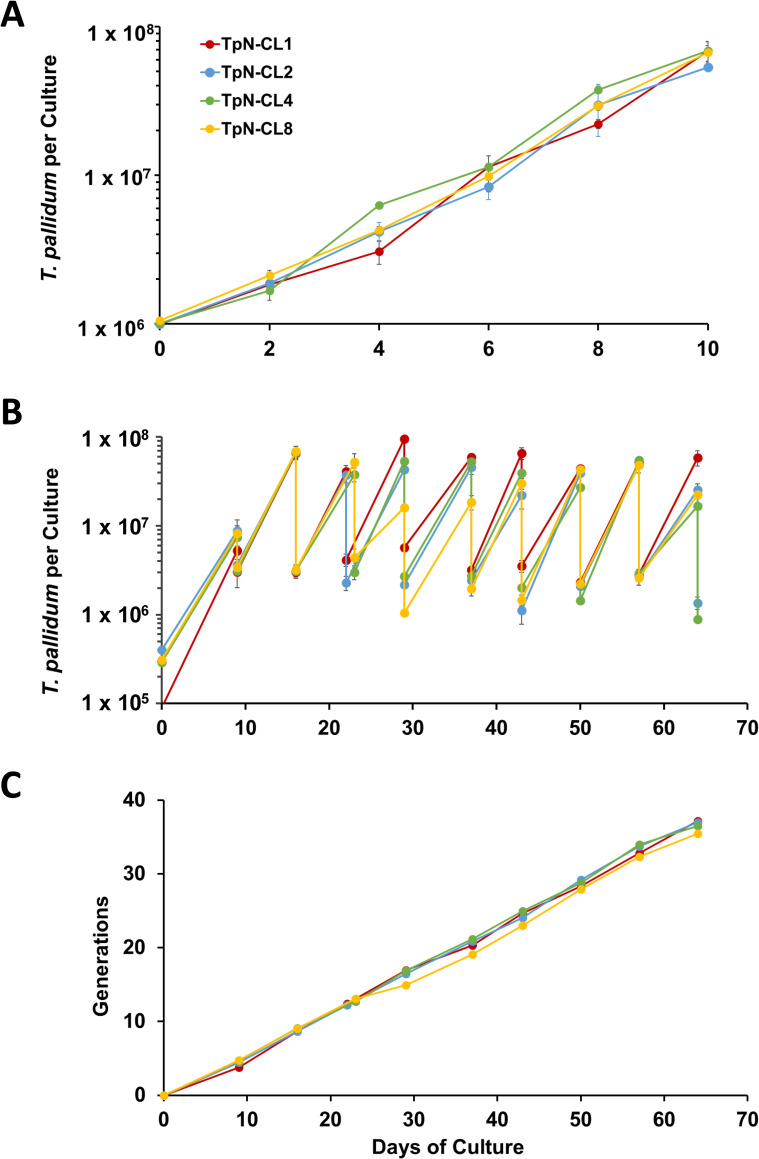
Growth patterns of *T*. *pallidum* Nichols clones TpN-CL1, TpN-CL2, TpN-CL4, and TpN-CL8 under standard culture conditions. (A) Near equivalent multiplication of clones during a single passage, as determined by harvest of triplicate cultures at 2-day intervals. (B) Serial passage of clones through 9 passages. Cultures were initiated from frozen stocks and subcultured on the days indicated. The higher value at each time point represents the total culture yield, and the lower value is the number of *T*. *pallidum* transferred to new cultures. (C) Cumulative number of generations obtained in the cultures shown in B. *T*. *pallidum* per culture values represent the mean ± S.E.M. of triplicate wells.

Four clones (TpN-CL1, TpN-CL2, TpN-CL4, and TpN-CL8) were examined for their growth rates *in vitro*. As shown in **Fig 2A**, the clones had no significant differences in growth rate over a 10-d culture period. The clones were further cultured for 9 serial passages (a total of 64 d) to determine if minor differences in long-term growth rates existed (**Fig 2B**). Average doubling times over the 64-d period were 47.2, 45.7, 43.7, and 44.0 h, respectively. Student’s t-test showed no significant differences (*p* > 0.05) in clone generation time, as determined by comparing the 9 generation time values obtained for each clone during the experiment in **Fig 2B**. In addition, the cumulative number of generations (**Fig 2C**) were similar over the entire culture period.

The infectivity of the *T*. *pallidum* clones was assessed using an intradermal lesion development model in rabbits. For each clone, the shaved backs of rabbits were inoculated intradermally with 10^5^, 10^3^, or 10 *T*. *pallidum* per site at a total of 4 sites, and the injection sites were observed daily for the development of lesions. The time of lesion development is inversely proportional to the number of infectious organisms injected, i.e., higher dosages produce lesions more rapidly. As shown in **Table 1**, all four clones examined produced lesions at all dosages. As observed in prior *T*. *pallidum* inoculation studies [27,31], the number of sites positive at the 10-organism inoculum varied from 4 of 4 to 1 of 4. These results are consistent with an approximate ID_50_ of 10 *T*. *pallidum* for these clones. Needle aspirates from representative lesions were consistently positive for motile treponemes by darkfield microscopy.

**Table 1:** Infectivity of *T*. *pallidum* Nichols clones in an intradermal infection rabbit model.

*T*. *pallidum* Dosage per Site	Lesions/Sites Inoculated	Day of Lesion Development	Average
**TpN-CL1**			
1 x 10^5^	4 / 4	11, 12, 12, 14	12.3
1 x 10^3^	4 / 4	13, 14, 14, 22	15.8
1 x 10^1^	4 / 4	14, 14, 19, 24	17.8
**TpN-CL2**			
1 x 10^5^	3 / 4	11, 11, 12	11.3
1 x 10^3^	4 / 4	12, 13, 13, 13	12.8
1 x 10^1^	2 / 4	20, 29	24.5
**TpN-CL4**			
1 x 10^5^	4 / 4	10, 11, 11, 12	11.0
1 x 10^3^	4 / 4	11, 12, 12, 12	11.8
1 x 10^1^	4 / 4	14, 15, 18, 20	16.8
**TpN-CL8**			
1 x 10^5^	4 / 4	10, 10, 11, 11	10.5
1 x 10^3^	4 / 4	12, 12, 12, 13	12.3
1 x 10^1^	1 / 4	34	34

Finally, to determine whether we could successfully generate isogenic lines of other *T*. *pallidum* strains, we repeated our cloning experiments using a *T*. *pallidum* strain of the SS14 clade, UW249B, and a second strain of the Nichols clade, Mexico A. After four weeks, five of 96 UW249B wells and four of 96 Mexico A wells were plated at 0.5 Tp/well were positive by qPCR and darkfield microscopy. Two potentially isogenic lines were successfully expanded from each strain; these will be characterized in future studies.

### Genomic sequencing

To examine the genomic variation among *T*. *pallidum* Nichols subpopulations, we performed whole genome sequencing of DNA isolated from the Nichols strain propagated in rabbits, TpN clones, and long-term cultures at various time points during the culture. The frozen stock of rabbit-propagated *T*. *pallidum* Nichols used for sequencing was the same one used to initiate the long-term *T*. *pallidum* cultures (and hence the clones). TpN clones 1, 2, 3, 4, 5 and 8 were selected for genomic sequencing. These six clones were chosen to provide a range of similar and different genotypes, based on the preliminary sequencing of PCR products as described above. In addition, four *in vitro* cultures from different time points of the IVA (days 137, 368, 473) and IVB (day 538) long-term culture lineages were sequenced. All of these preparations were subjected to Illumina sequencing; the clones were further analyzed by PacBio long-read circular consensus sequencing (CCS) to aid in the resolution of long repeat regions and indels. Unfortunately, the amounts of DNA available from the corresponding long-term IVA and IVB culture preparations were insufficient to perform long-read sequencing. However, PacBio CCS data was obtained from two additional long-term samples at 1274 days (IVB d1274) and 1288 days (IVA d1288) of continuous *in vitro* culture.

The general properties of the sequences from the preparations are provided in **S1 Table**. As expected, the rabbit-derived and *in vitro* culture-derived preparations of *T*. *pallidum* contained high concentrations of DNA from New Zealand white rabbits and Sf1Ep cottontail rabbit epithelial cells, respectively, since no purifications steps (other than low-speed centrifugation to remove intact rabbit cells and high speed centrifugation and washes to concentrate the *T*. *pallidum*) were undertaken to avoid possible sequence bias introduced by potential sequence-specific biases in hybridization or amplification procedures. As a result, the proportion of sequences mapping to the *T*. *pallidum* genome ranged from 4.6 to 91.4 percent, depending on the preparation. The high proportion of *T*. *pallidum* sequences in the TpNRabbit preparation (91.4%) was likely due to a combination of high *T*. *pallidum* concentration in the extract and the 10X-greater volume of phosphate-buffered saline (PBS) used during the high speed centrifugation wash step, which may have been more effective in removing free rabbit DNA present in the tissue extract.

The average coverage for the Illumina sequencing was 183.3 (range 76.2–646.0), whereas the coverage for PacBio CCS sequencing was 0.9–15.1 (**S1 Table**). All the assembled genome sequences were highly similar and exhibited identical synteny in terms of gene order. However, intrastrain heterogeneities were present within the rabbit-derived *T*. *pallidum* preparation as well as within each of the *in vitro* culture populations (**Table 2**). In contrast, each clone was homogeneous in its genomic sequence, with the exception of a heterogeneity in the number of *TP0470* repeat sequences in TpN-CL4. There were sequence differences between the clones, consistent with the co-existence of genetically distinct subpopulations within the Nichols strain.

**Table 2:** Nucleotide polymorphisms identified among uncloned and cloned populations of *T*. *pallidum* subsp. *pallidum* Nichols. ^a^ Genotypes consistently present in *T*. *pallidum* strains other than the Nichols strain are indicated in blue, and the Nichols strain-specific genotypes are marked in yellow. The presence of heterogeneity is indicated as orange.

Location in NC_021490.2	Gene (*TP*) or Intergenic Region (IGR)	Common *T*. *pallidum* genotype	Nichols strain-specific mutation	Nichols reference sequence NC_021490.2		TpN_Rabbit	IVA_d137^b^	IVA_d368	IVA_d473	IVB_d538		TpN-CL1	TpN-CL3	TpN-CL4	TpN-CL2	TpN-CL5	TpN-CL8	Other GenBank entries with Nichols strain-specific mutation
**Deletions and Repeat Sequences**																		
**148537–149740**	***0126d* (HP), *0128* (HP)**	**No deletion**	**1204-nt deletion**	**No deletion**		**ND** ^**c**^	**ND**	**ND**	**ND**	**ND**		**1204-nt deletion**	**No deletion**	**No deletion**	**No deletion**	**No deletion**	**No deletion**	**AE000520.1 (TpN Fraser)** **CP010560.1 (TpN Clone E)**
**157653**	**IGR** ***0135-t0009***	**1 63-nt sequence**	**2 63-nt sequences**	**1 63-nt sequence**		**1 63-nt sequence (43%)** **2 63-nt sequences (57%)**	**ND**	**ND**	**2 63-nt sequences**	**1 63-nt sequence**		**2 63-nt sequences**	**2 63-nt sequences**	**2 63-nt sequences**	**1 63-nt sequence**	**1 63-nt sequence**	**1 63-nt sequence**	**Variable among strains: 1 copy (69.4%); 2 copies (19.8%); 3 copies (10.8%)**
**498847**	***0470*** **HP**	**Variable repeat number**	**Variable repeat number**	**17 repeats** **(24-nt)**		**17 repeats (50%)** **19 repeats (40%)** **23 repeats (10%)**	**ND**	**ND**	**ND**	**17 repeats**		**17 repeats**	**16 repeats**	**14 repeats (80%)** **13 repeats (20%)**	**16 repeats**	**18 repeats**	**16 repeats**	**Variable number of repeats; some match clone sequences**
**974054**	***0893*** ***rimP***	**CACGCACG**	**CACG**	**CACGCACG**		**CACGCACG (74%)** **CACG (26%)**	**CACGCACG (76%)** **CACG (24%)**	**CACGCACG (90%)** **CACG (10%)**	**CACGCACG (80%)** **CACG (20%)**	**CACGCACG (7%)** **CACG (93%)**		**CACGCACG**	**CACGCACG**	**CACGCACG**	**CACG**	**CACG**	**CACG**	
**975823–975889**	**IGR** ***0896–0897***	**No deletion**	**67-nt deletion**	**No deletion**		**No deletion (75%)** **67-nt deletion (25%)**	**No deletion (92%)** **67 nt deletion (8%)**	**No deletion**	**No deletion**	**No deletion (6%)** **67 nt deletion (94%)**		**No deletion**	**No deletion**	**No deletion**	**67-nt deletion**	**67-nt deletion**	**67-nt deletion**	**CP010422.1 (TpN Seattle)** **AF194364.1 (Tpp Bal7 124)** **AF194365.1 (Tpp Bal7 126)**
**Single Nucleotide Variations**																		
**7179**	***0006* HP** **(stop codon)**	**C**	**T**	**T**		**C (32%)** **T (68%)**	**C (43%)** **T (57%)**	**C (7%)** **T (93%)**	**C (9%)** **T (91%)**	**C (99%)** **T (1%)**		**T**	**T**	**T**	**C**	**C**	**C**	**AE000520.1 (TpN Fraser)** **CP010560.1 (TpN Clone E)**
**59894**	***0051*** ***prfA***	**C**	**T**	**T**		**C (34%)** **T (66%)**	**C (36%)** **T (64%)**	**C (14%)** **T (86%)**	**C (9%)** **T (91%)**	**C**		**T**	**T**	**T**	**C**	**C**	**C**	**AE000520.1 (TpN Fraser)** **CP010560.1 (TpN Clone E)**
**149360**	**IGR** ***0126d- 0126c***	**T**	**C**	**C**		**T**	**T**	**T**	**T**	**T**		**Region deleted**	**T**	**T**	**T**	**T**	**T**	
**228259**	***0222*** **HP**	**A**	**C**	**A**		**A (72%)** **C (28%)**	**A (63%)** **C (37%)**	**A (85%)** **C (15%)**	**A (88%)** **C (12%)**	**A (4%)** **C (96%)**		**A**	**A**	**A**	**C**	**C**	**C**	
**307493**	***0292*** ***ompF***	**A**	**G**	**A**		**A**	**A**	**A**	**A**	**A**		**A**	**A**	**G**	**A**	**A**	**A**	
**463184**	***0433*** ***arp***	**C**	**T**	**C**		**C**	**C**	**C**	**C**	**C**		**C**	**C**	**C**	**T**	**T**	**T**	
**500905**	***0471*** **TPR domain protein**	**G**	**A**	**G**		**G (70%)** **A (30%)**	**G (52%)** **A (48%)**	**G (91%)** **A (9%)**	**G (84%)** **A (16%)**	**G (1%)** **A (99%)**		**G**	**G**	**G**	**A**	**A**	**A**	**CP010561.1 (TpN CloneJ)**
**523669**	***0488*** ***mcp2***	**A**	**C**	**A**		**A (72%)** **C (28%)**	**A (55%)** **C (45%)**	**A (93%)** **C (7%)**	**A (76%)** **C (24%)**	**A (3%)** **C (97%)**		**A**	**A**	**A**	**C**	**C**	**C**	
**534222**	***0497*** ***mreB***	**C**	**T**	**C**		**C**	**C**	**C**	**C**	**C (2%)** **T (98%)**		**C**	**C**	**C**	**C**	**C**	**C**	
**536766**	***0500*** ***mrdA***	**G**	**A**	**G**		**G**	**G (96%)** **A (4%)**	**G**	**G**	**G (98%)** **A (2%)**		**A**	**G**	**G**	**G**	**G**	**G**	
**635418**	***0548* HP (promoter)**	**T**	**G**	**T**		**T (48%)** **G (52%)**	**T (71%)** **G (29%)**	**T (98%)** **G (2%)**	**T (99%)** **G (1%)**	**T (99%)** **G (1%)**		**G**	**T**	**T**	**T**	**T**	**T**	**AE000520.1 (TpN Fraser)** **CP010560.1 (TpN Clone E)**
**700635**	***0639*** ***mcp3***	**C**	**T**	**C**		**C**	**C**	**C**	**C**	**C**		**C**	**C**	**C**	**T**	**C**	**C**	
**767985**	**IGR** ***0698–0700***	**A**	**G**	**G**		**A (31%)** **G (69%)**	**A (35%)** **G (65%)**	**A (6%)** **G (94%)**	**A (17%)** **G (83%)**	**A (98%)** **G (2%)**		**G**	**G**	**G**	**A**	**A**	**A**	**AE000520.1 (TpN Fraser)** **CP010560.1 (TpN Clone E)** **CP010561.1 (TpN CloneJ)**
**810772**	***0746*** ***ppdk***	**C**	**T**	**C**		**C (99%)** **T (1%)**	**C (95%)** **T (5%)**	**C (23%)** **T (77%)**	**C (17%)** **T (83%)**	**C**		**C**	**C**	**T**	**C**	**C**	**C**	
**811414**	***0746*** ***ppdk***	**G**	**A**	**G**		**G**	**G**	**G**	**G**	**G (98%)** **A (2%)**		**A**	**G**	**G**	**G**	**G**	**G**	
**874548**	***0804*** ***ugpC***	**A**	**G**	**A**		**A**	**A**	**A**	**A**	**A**		**G**	**A**	**A**	**A**	**A**	**A**	
**884280**	***0814 txrB* (promoter)**	**C**	**T**	**C**		**C**	**C**	**C (97%)** **T (3%)**	**C (98%)** **T (2%)**	**C (2%)** **T (98%)**		**C**	**T**	**C**	**C**	**C**	**C**	
**975954**	***0897*** ***tprK***	**C**	**A**	**C**		**C (89%)** **A (11%)**	**C (91%)** **A (9%)**	**C (96%)** **A (4%)**	**C (99%)** **A (1%)**	**C (97%)** **A (3%)**		**A**	**C**	**C**	**C**	**C**	**C**	
**1057089**	***0972*** ***ftr1***	**C**	**T**	**C**		**C**	**C**	**C**	**C**	**C**		**C**	**C**	**C**	**T**	**T**	**T**	**AE000520.1 (TpN Fraser)**
**1057375**	***0972*** ***ftr1***	**G**	**A**	**G**		**G**	**G**	**G**	**G**	**G**		**G**	**A**	**G**	**G**	**G**	**G**	

a Homopolymeric tract and *tprK* polymorphisms are addressed in separate tables or figures. The column labeled “Reference” refers to the sequence the *T*. *pallidum* Nichols genome (GenBank No. NC_021490.2) as previously determined by Pětrošová et al. [36]. “Location” is the nucleotide number in this reference sequence, which may differ from the corresponding nucleotide number in the other genomes because of preceding relative indels. “Gene” is the abbreviated gene name (e.g. *0006* means *TP0006*) and annotation. In the subsequent columns, heterogeneity is indicated by the genotypes occurring at that position followed by the corresponding percentage of reads. A single genotype without a percentage indicates that >99% of the reads were consistent with that genotype.

b IVA = *In vitro* culture lineage A, begun 20 Oct 2017. IVB = *In vitro* culture lineage B, begun 3 Nov 2017. The day of culture in which *T*. *pallidum* were harvested for DNA extraction are indicated (e.g. d137).

c Not determined due to lack or deficiency of long read data.

**Fig 3:**
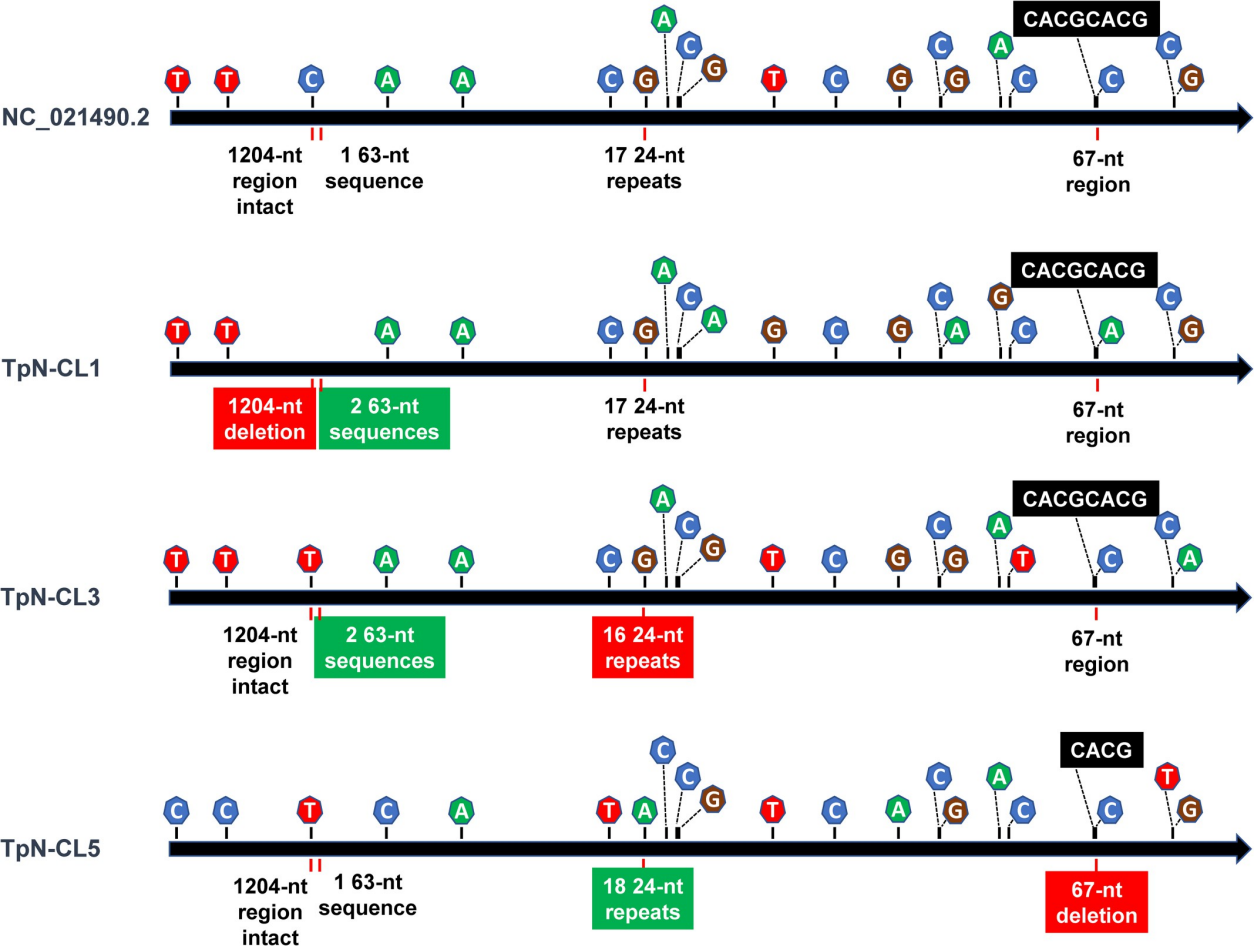
Heterogeneities in *T*. *pallidum* clones. Linearized map of the circular chromosome of *T*. *pallidum* Nichols reference sequence (NC_021490.2) and three representative clones, showing the locations of heterogeneities. The locations of single nucleotide variants (SNVs) and small indels are indicated above the arrow representing each genome, whereas the larger indels are shown below the arrow. The precise locations of the 20 SNVs and 5 indels are provided in Table 2.

The distribution of the polymorphisms in the chromosome is depicted in **Fig 3**, using the Pětrošová et al. [36] reference sequence and three divergent clones as examples. These differences consisted of insertions and deletions (indels) ranging from 4 nt to 1,204 nt in length, single nucleotide variants (SNVs), heterogeneities in the lengths of homopolymeric tracts (predominantly in polyG/polyC regions), and differences in *tprK* variable region (VR) sequences. Heterogeneities within each clone were limited to the VRs of *tprK*, a few polymorphisms in polyG/polyC homopolymeric regions, and (in the case of TpN-CL4) presence of varied numbers of repeats in a 24-nt repeat region. Most of these results are summarized in **Table 2**; the homopolymeric tracts and *tprK* heterogeneities are addressed separately because of their greater complexity.

We were surprised to find that the SNVs found within the Nichols strain were strain-specific (**Table 2**). The Nichols strain-specific alleles were not found in **any** non-Nichols strain sequence, including the other strains within the Nichols-like cluster. These alleles are shown in the Nichols strain-specific mutation column and are marked in yellow in **Table 2**. Those sites that had the genotype common to all the other *T*. *pallidum* strains are marked in blue. The TpNRabbit and in vitro culture preparations contained mixtures of the two genotypes at multiple positions, as indicated by the presence of both blue and yellow coloration as well as the corresponding percentages. The 1204 nt deletion identified in TpN-CL1, the initial Nichols strain genome sequence [7], and the Nichols strain Clone E sequence was not found in any other *T*. *pallidum* strain sequence. The 4-nt deletion at nt 974054 was also unique to the Nichols strain. The other indels were found in other strains (**Table 2**).

### Insertions and deletions

**Fig 4:**
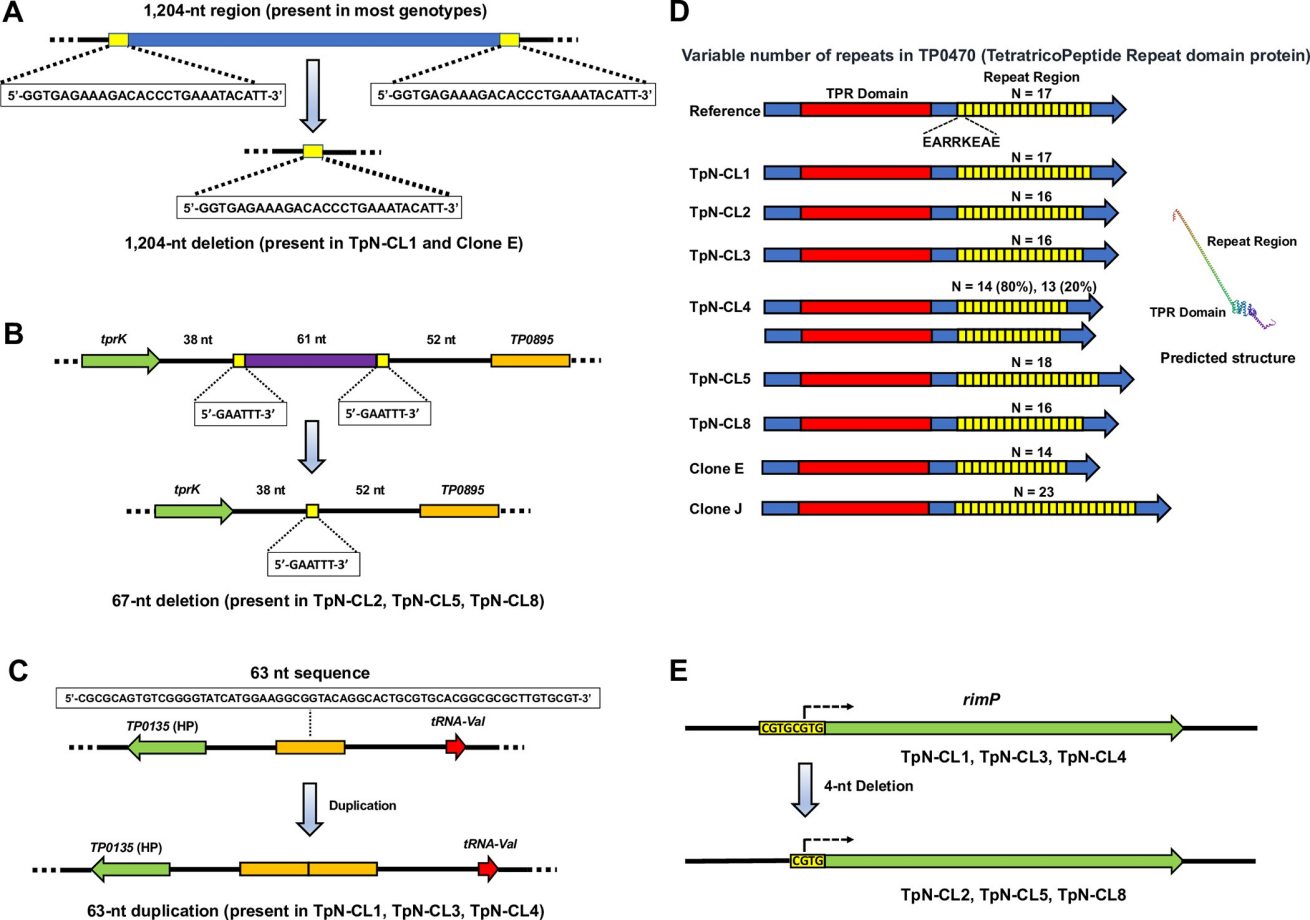
Insertions and deletions present in the *T*. *pallidum* Nichols genome. (A) A 1,204 nt deletion present in TpN-CL1 and *T*. *pallidum* Nichols Clone E (CP010560.1), resulting from recombination between two 33-nt direct repeats. (B) Deletion of a 67-nt region downstream of *tprK*; the deleted region is flanked by a 5-nt direct repeat. (C) Duplication of a 63-nt intergenic region near *TP0135*. (D) Occurrence of varied number of 24-nt repeats with *TP0470*; the inset shows the predicted structure of the TP0470 protein product. (E) A 4-nt indel at the beginning of *TP0893* (*rimP*) that may alter its promoter and RBS spacing, but does not change the start codon.

Five examples of insertions or deletions (indels) were identified that resulted in heterogeneities among the 6 *T*. *pallidum* Nichols clones examined. The largest of these was found in TpN-CL1 and consisted of a 1,204-nt deletion in the region corresponding to nt 148537–149740 in the NC_021490.2 reference sequence. The recombination that resulted in this 1,204-nt deletion appears to have occurred between two identical copies of a 33 nt sequence (5’-GGTGAGAAAGACACCCTGAAATACATT-3’) that flank the deleted region; these flanking direct repeat sequences are present in the Pětrošová et al. [36] genome sequence and all of our other clonal sequences. In TpN-CL1, the flanking direct repeats and all of the intervening region are replaced by a single copy of the 33-nt sequence, consistent with a single crossover event between the two homologous sequences (**Fig 4A**). This relative indel was first noted by Centurion-Lara et al. [9] as a difference between the previously published Nichols strain sequence [7] (which lacked the 1,204 nt region) and the Nichols Seattle (NicholsSea) and Chicago strain sequences (which contained this region). Mikalová et al. [37] later verified the existence of subpopulations within the Nichols strain with the deletion, and also noted that the deletion was not present in other *T*. *pallidum* genomic sequences available at the time; indeed, the many genome sequences obtained to date indicate that this deletion is a rare genetic abnormality not present in other strains. Of all of the previously available *T*. *pallidum* genomic sequences, only the first Nichols strain genome sequence by Fraser et al. [7] (AE000520.1) and the sequence of the Nichols strain Clone E (CP010560.1) contain this large deletion. Evidence for this deletion was also present in our rabbit-propagated *T*. *pallidum* population; however, the long read (PacBio) coverage in this region was too sparse to provide a numerical value for this occurrence. The deletion present in TpN-CL1 eliminates 20 of the 54 donor sequences available for sequence variation of *tprK* in other Nichols genome sequences [9,12–14]. Thus TpN-CL1 has a reduced number of donor sequences for *tprK* variation, yet is still infectious in the rabbit model (**Table 1**).

A 67-nt deletion (at nt 975817–975883 of the reference sequence) is located in an intergenic region 38 nt downstream of the stop codon of *tprK* and 52 nt upstream of *TP0895*, encoding a DUF302 domain-containing protein of unknown function (**Fig 4B**). The deletion is consistently present in TpN-CL2, TpN-CL5, and TpN-CL8 and is absent in the other clones sequenced; it was also found in ~5% of the sequences from rabbit-propagated *T*. *pallidum*. The deletion apparently occurred as the result of a single crossover event between two copies of the sequence 5’-GAATTT-3’ that flank the area of that is deleted from the ‘wild type’ sequence (**Fig 4B**); only a single copy of this 6-nt sequence is present in all reads containing this mutation. The deleted segment does not contain recognizable promoter sequences or other aspects that provide insight into a possible associated function, and its removal does not result in any obvious phenotypic changes. This 67-nt deletion is also present in 25% of the TpNRabbit sequences (**Table 2**) and in two previously reported sequences of the *tprK* region from *T*. *pallidum* subsp. *pallidum* Baltimore 7 strain [38] (GenBank entries AF194364.1, AF194365.1).

A single, 63-nt sequence present at nt 157653 of the reference sequence is duplicated as two consecutive copies in clones TpN-CL1, TpN-CL3, and TpN-CL4 (**Fig 4C**); it was also found in 57% of the reads in the rabbit-propagated population (Rb6L105) and was predominant in the *in vitro* culture IVA_d473 (**Table 2**). Single copies of the 63-nt region were found in the reference sequence NC_021490.2 as well as in clones TpN-CL2, TpNCL5, and TpNCL8 and 43% of the rabbit-propagated Nichols strain. BLASTn searches of the complete *T*. *pallidum* genome sequences currently available in the NCBI database revealed heterogeneity in this region, with strains having 1, 2, or 3 copies (**Table 2**). Therefore, variation in the number of 63-nt tandem repeats in this region is a common difference among *T*. *pallidum* strains. This sequence feature is located in an unannotated region between *TP0135* (encoding a hypothetical protein) and *TPt0009* (tRNA-Val), and any functional effects of this heterogeneity have not as yet been identified.

*TP0470* is annotated as a tetratricopeptide repeat protein (TPR) domain protein **(Fig 4D**) and contains a variable number of copies of a 24-nt repeat (5’- GAAGCCCGGCGCAAGGAGGCGGAG -3’, encoding the highly charged sequence EARRKEAE). The TPR domain (a common motif involved in protein-protein interactions) is located in the N-terminal region of the predicted TP0470 protein, whereas the 8 aa repeats are present in the C-terminal region. We used PacBio sequencing to determine the number of *TP0470* 24-nt repeats, since the shorter 150-nt Illumina reads led to ambiguous assemblies in this region. Thus this analysis was dependent upon the relatively small number of PacBio reads obtained. The number of repeats found in the TpN clones ranged from 13 to 18 (**Fig 4D**, **Table 2**). TpN-CL4 was the only clone that exhibited heterogeneity, with 80% of reads having 14 repeats and 20% having 13 repeats. This region represented the only difference (besides heterogeneities in *tprK* and homopolymeric tracts) present between TpN-CL5 and TpN-CL8, which had 18 and 16 copies of the 24-nt repeat respectively.

The gene *TP0433* encodes the Acidic Repeat Protein (Arp), which contains a region of 60 nt direct repeats that translate to 20 amino acid sequences [39]. In the NC_021490.2 reference sequence, there are 14 such repeats, along with a 9 nt partial repeat. The repeats are not identical, differing from each other by one to three amino acids; the most common repeat sequence is REVEDVPKVVEPASEREGGE. In our study, variation within the *TP0433* repeat region was analyzed using a set of PacBio reads that contained the full-length sequence of the gene; there were a total of 54 such sequences in all the *T*. *pallidum* specimens examined. The *TP0433* repeat regions in all the *T*. *pallidum* clones were identical to the reference sequence in both the number and order of repeat variants; this was also the case for the rabbit-derived *T*. *pallidum* sequences. Some differences did exist in the uncloned in vitro culture preparations. The TpNIVB_d1274 reads (N = 4) all contained 19 repeats instead of 14; one of these sequences also contained a 5-nt insert near the beginning of the repeat region that would lead to a frameshift and a truncated protein product. One of the TpNIVA_d1288 sequences had a deletion that encompassed nearly all of the repeat region and also caused a frameshift. Although the number of sequences available for this analysis was limited, the results indicate the *T*. *pallidum* Nichols clones examined retained the predominant *TP0433* repeat region sequence whereas variation in this region was observed in the uncloned in vitro cultures.

A small, 4-nt indel heterogeneity was observed at nt 974054 in the reference sequence (**Fig 4E**). This indel encompasses the predicted start codon of gene *TP0893*, encoding a RimP, predicted to be a ribosomal maturation factor. The reference sequence contains a duplicated 4-nt sequence (CACG), which in the reverse orientation of *TP0893* reads 5’-CGTGCGTG-3’; the predicted start codon is underlined. Deletion of one copy of the 4-nt repeat is present in the genomic sequences of TpN clones 2, 5, and 8, as well as in a considerable proportion of the rabbit-propagated and *in vitro* culture derived *T*. *pallidum* populations (**Table 2**). This change (to 5’-CGTG-3’) would result in the same predicted start codon (underlined) and thus would not affect the reading frame. However, this deletion would change the spacing of any upstream promoter and ribosome binding site (RBS) sequences relative to the start codon, with a potential impact on gene expression.

### Single nucleotide variations (SNVs)

In comparing all the genomic sequences reported in this study, there were a total of 20 SNVs scattered throughout the length of the aligned genomes (**Fig 2**). The precise locations of these are indicated in **Table 2**. Using the SNV at nt 7179 as an example, the Pětrošová et al. [36] reference sequence (NC_021490.2) has a T at that position. Our rabbit-propagated *T*. *pallidum* preparation (TpRb6L105) contained a mixture of T (68%) and C (32%) reads at this location. Similar polymorphisms were observed in the rabbit-derived specimen at 9 of 11 SNV positions.

For the *in vitro* cultures, the mixture of genotypes was initially similar to that of the rabbit-propagated organisms, as exemplified by the IVA_d137 sample. However, there tended to be a decrease in the heterogeneity as the number of days of culture increased, resulting in a more homogeneous genotype. For example, IVA_d473 had 91% T and 9% C at the nt 7179 location. Only a single time point, at day 538 of culture, was examined for the IVB lineage. Interestingly, the SNV ratio changed in the opposite direction in this lineage, and was more extreme (99% C, 1%T). This pattern held for the other SNV sites in this sample, and was also observed for the 4-nt indel at the location 974054 and a 67 nt indel at 975817–975883 (**Table 2**). The IVA and IVB cultures were maintained in a near identical manner, although each had different periods of low *T*. *pallidum* numbers that may have resulted in ‘bottlenecks’. It is possible that these bottlenecks resulted in random selection of different subpopulations within the initial inocula.

### Homopolymeric tracts

DNA replication errors occur at a higher rate at tracts of single nucleotides, such as polyG stretches. In this study, variations in the lengths of polyG and polyC tracts (hereafter called polyG/C tracts, due to their antiparallel strand symmetry) occurred in the assembled genome sequences (**S2 Table)**; no differences were observed in shorter tracts of these nucleotides, or in any of the polyA or polyT homopolymeric tracts. Although sequencing errors are also more common in polyG/C tracts, we found that the results were remarkably consistent within each clone, with >90% of the reads having the same homopolymeric length at nearly all locations. Length variants was present at 24 of 46 (52%) of the polyG/C tracts of ≥8 nucleotides, and each of the TpN clones exhibited a different set of polyG/C length variations (**S2 Table**). Most of the polyG/C tracts that exhibited variation in length were within intergenic regions (IGRs), but 6 of the 22 polyG/C variations in TpN clones were within predicted genes and would thus affect the reading frame of the gene. Five of these were within hypothetical proteins with no predicted function, so it is not possible to predict the biologic effects of the resulting frameshifts. One polyG/C variation was within *mcp1*, encoding a methyl-accepting chemotaxis protein. In the reference sequence NC_021490.2 and in TpN-CL3, this gene encodes an 812 aa protein, whereas in all of the other TpN clones the polyG/C difference results in a different C-terminus and an 807 aa predicted protein. Because the C-terminus is involved in CheW interactions and signaling, chemotaxis may be affected in *T*. *pallidum* bearing this variation. It is of course possible that variations in the lengths of polyG/C tracts in intergenic regions could affect gene transcription or translation, as has been indicated for such tracts located upstream of *TP0126* (encoding an OmpW homolog) and subfamily II *tpr* genes in experiments using a heterologous expression system in *Escherichia coli* [40,41].

### *tprK* sequence variation

Sequence differences occurring in each genomic sequence with *tprK* were examined using two approaches. In the first method, sequences corresponding to each variable region (VR1 through VR7) were extracted from the Illumina data, aligned, and analyzed for the number of different variants and their proportions (as percent of the total reads analyzed). For the second method, reads containing the entire *tprK* open reading frame were obtained from the PacBio CCS data. The longer read length for this sequencing method made it possible to examine which VR variants occurred together, whereas this linkage could not be discerned from the shorter read Illumina data. In both procedures, sequences that differed by only a single nucleotide from the most common sequence and were found in only one read were excluded, because of the high probability that these differences represented sequencing errors.

**Fig 5:**
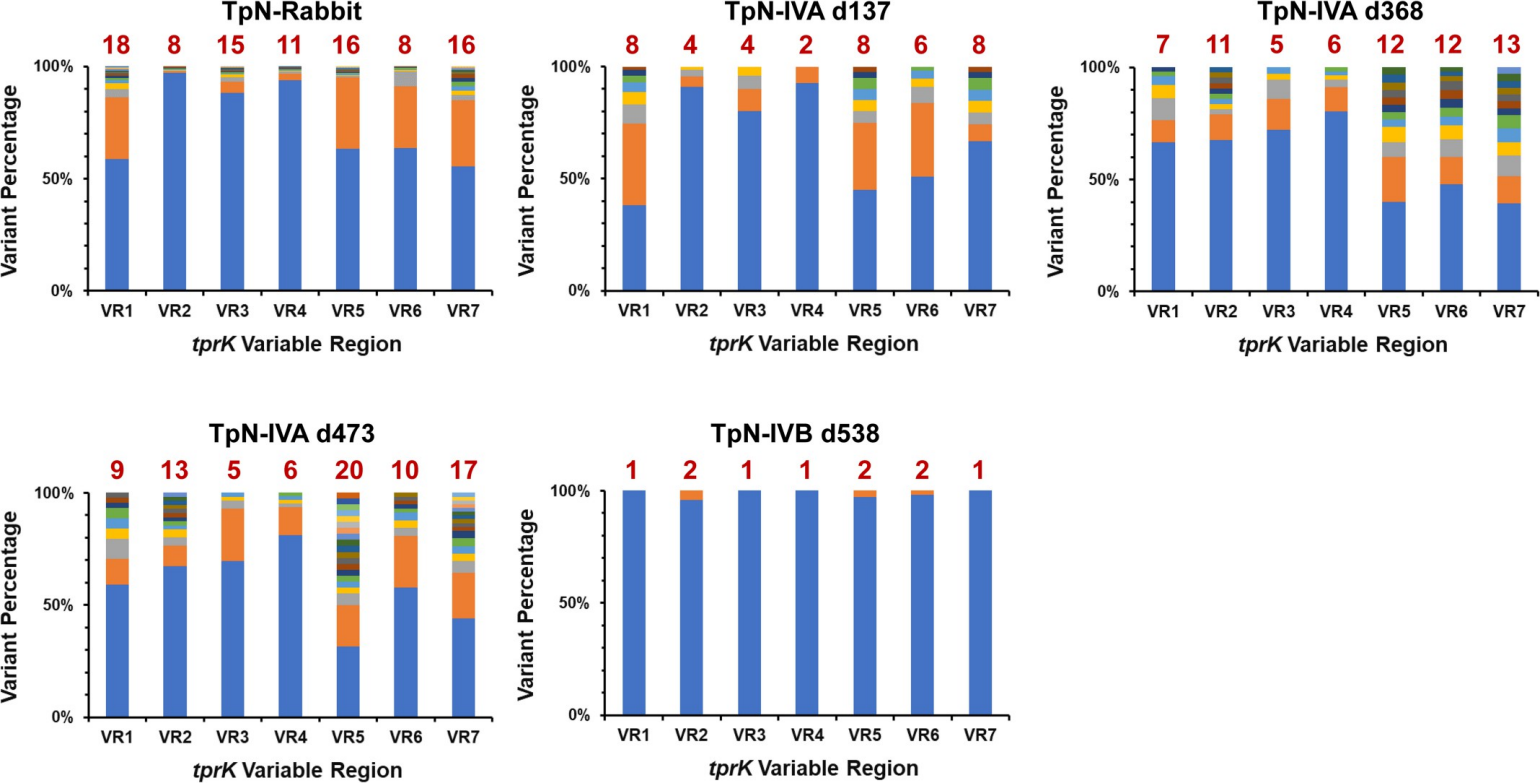
Sequence variation in the *tprK* variable regions (VR1 to VR7) in uncloned populations of *T*. *pallidum* Nichols. Results were obtained with Illumina sequencing of rabbit-propagated organisms (TpN-Rabbit) and two independent *in vitro* culture lineages (TpN-IVA and TpN-IVB) sampled after the indicated number of days of *in vitro* culture. The numbers above the bars indicate the number of different variable region sequences detected. The bar colors are arranged from most frequent to least frequent variant, and do not denote sequence identity or similarity. The single time point obtained in the TpN-IVB lineage had considerably fewer variants than observed in the TpN-IVA time points.

In the VR analysis, the rabbit-propagated and *in vitro* culture-derived Nichols strain genomic *tprK* sequences generally exhibited a high degree of variability, as measured by both the number of different variants detected and their relative proportions (**Fig 5**). An exception to this pattern was the IVB_d538 *in vitro* culture preparation, in which only 1 or 2 VR sequences was detected for each variable region, and the predominant VR represented 96 to 100 percent of the total.

**Fig 6:**
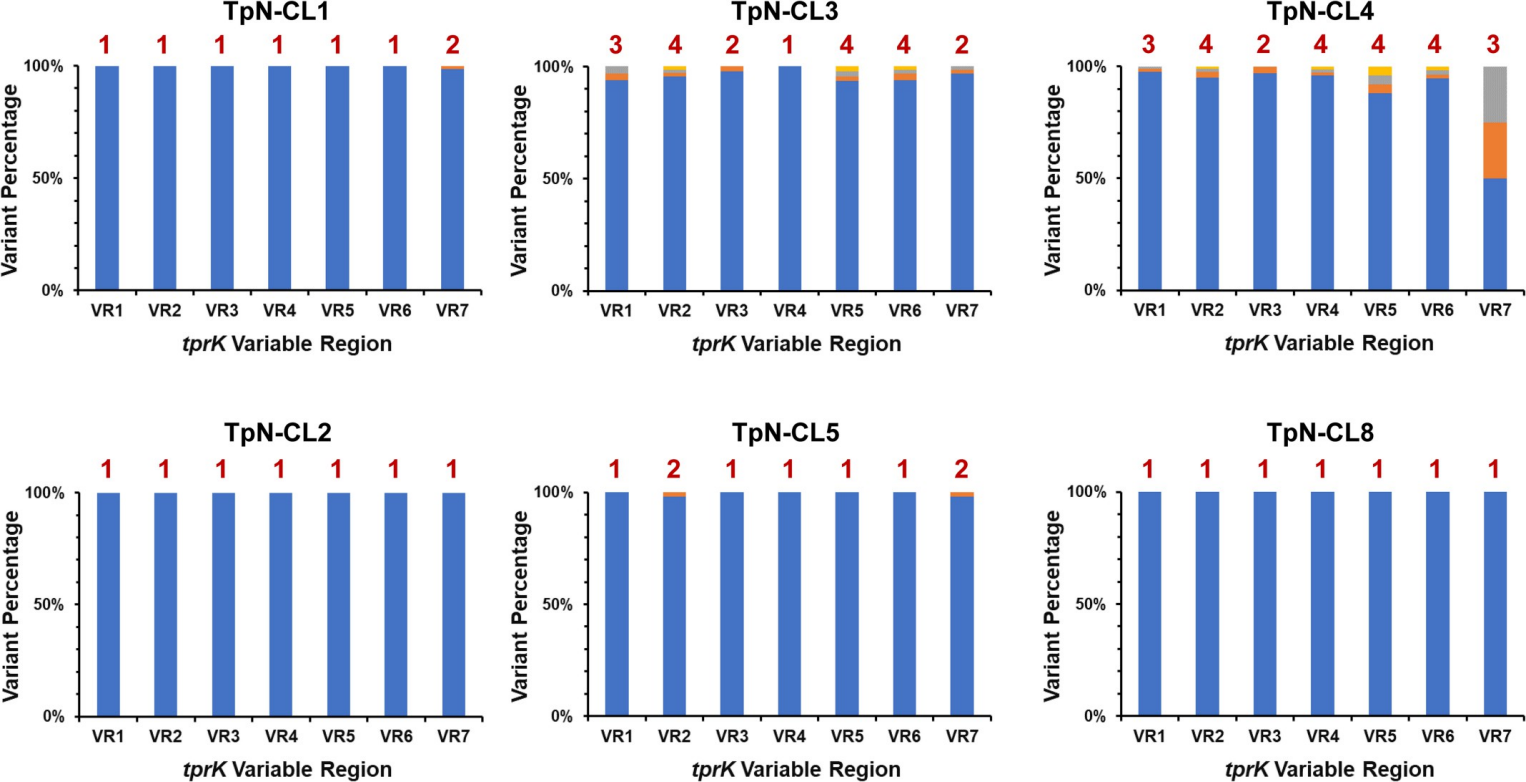
*tprK* variable regions in the *T*. *pallidum* Nichols clones exhibit a low degree of sequence variations. Results were obtained with Illumina sequencing of six *T*. *pallidum* Nichols clones generated *in vitro*. The numbers above the bars indicate the number of different variable region sequences detected. The bar colors are arranged from most frequent to least frequent variant, and do not denote sequence identity or similarity. Either a single or (rarely) two variable region sequences were detected in clones TpN-CL1, TpN-CL2, TpN-CL5, or TpN-CL8, whereas TpN-CL3 and TpN-CL4 had between one and four different variants within each variable region.

The VR content of the TpN clones exhibited a pattern of relatively high homogeneity within each clone (**Fig 6**). The tprK VR sequences of TpN clones 2, 5, and 8 were highly homogeneous, with the predominant VR sequences of these clones being identical to each other. In this group, only TpN-CL5 had any variation from the predominant sequence, with only rare variants being present in VR2 and VR7. TpN-CL1 was also relatively invariant, with a single rare variant seen at VR7. The clones TpN-CL4 and TpN-CL3 exhibited sequence variation at nearly all the VRs, with TpN-CL4 showing the highest heterogeneity in VR7 **(Fig 6**). These results provide evidence that *tprK* sequence variation can occur during *in vitro* culture, and that the amount of variation may differ in individual clones.

**Fig 7:**
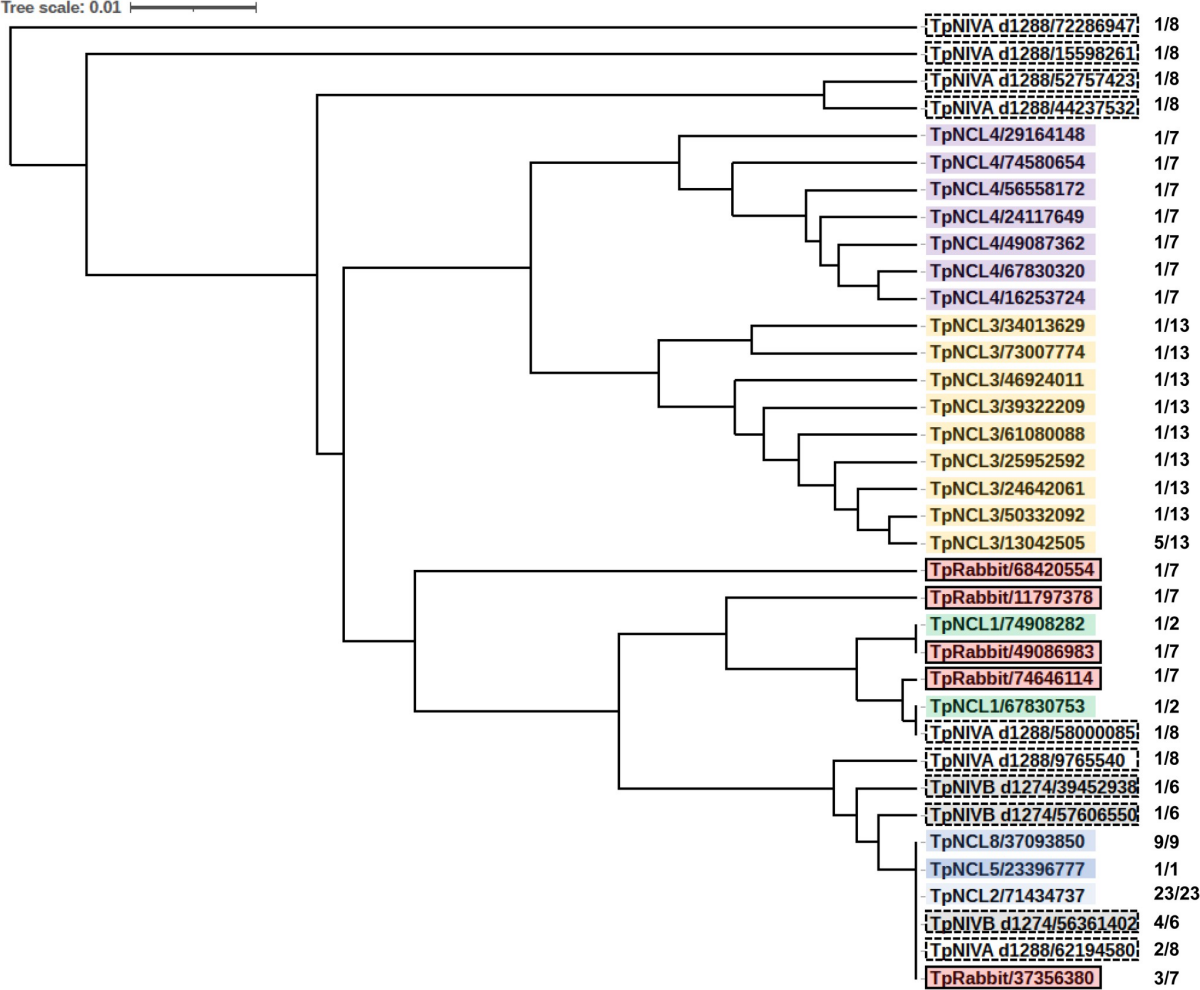
Relatedness of the full length *tprK* sequences of the *T*. *pallidum* Nichols clones and uncloned preparations. The nucleotide sequences of all available full length *tprK* sequences from PacBio CCS data were aligned, analyzed for relatedness using the UPGMA algorithm, and then depicted as an unrooted phylogenetic tree. The sequences from each clone are color coded; the rabbit-derived preparation is marked by red boxes, whereas the *in vitro* culture preparations are in shaded, dashed boxes. The PacBio identification number of a read for the sequence is included in each box; these are the same as the read IDs in S1 and S2 Figs. The numbers at the far right indicate the number of identical reads over the total number of available full-length *tprK* reads from that DNA preparation. The results indicate that the sequences from each of the clones are closely related, consistent with their derivation from a single precursor *tprK* sequence. In addition, identical full length *tprK* sequences were detected for clones TpN-CL2, TpN-CL5, and TpN-CL8.

Most studies of *tprK* sequence variation have examined each variable region separately, because short-read sequences such as those provided by Illumina sequencing do not permit assembly of the entire open reading frame of this divergent gene. The availability of long-read CCS sequences in this study permitted the assessment of the full-length *tprK*. A total of 76 full-length reads of the *tprK* ORF were obtained, including those from the TpNRabbit preparation, IVA_d1288, TpN IVB_d1274, and the six TpN clones. The resulting nucleotide and predicted amino acid sequences were aligned (**S1** and **S2 Figs**) and subjected to phylogenetic analysis (**Fig 7**). The UPMGA phylogenetic analysis algorithm was used rather than nearest neighbor analysis as the UPMGA process includes the analysis of regions with gaps, which are common in *tprK* alignments. This information reinforced the VR analysis by showing that clones TpN-CL2, TpN-CL5, and TpN-CL8 and certain rabbit- and *in vitro*-culture derived clonotypes were identical and thus highly conserved in their *tprK* sequences, whereas TpN-CL3 and TpN-CL4 each represented a separate, diverse cluster of *tprK* genotypes (**Fig 7**). The existence of these clusters is consistent with each of these two clones beginning with a single *tprK* genotype and then diverging during *in vitro* culture. The data for TpN-CL1 were limited to two sequences that showed closely related, yet different, sequences. Overall, our *tprK* results confirm those in the prior study by Lin et al. [10], indicating that *tprK* sequence variation can occur during *in vitro* culture.

### Comparison of clonal genomic sequences

**Fig 8:**
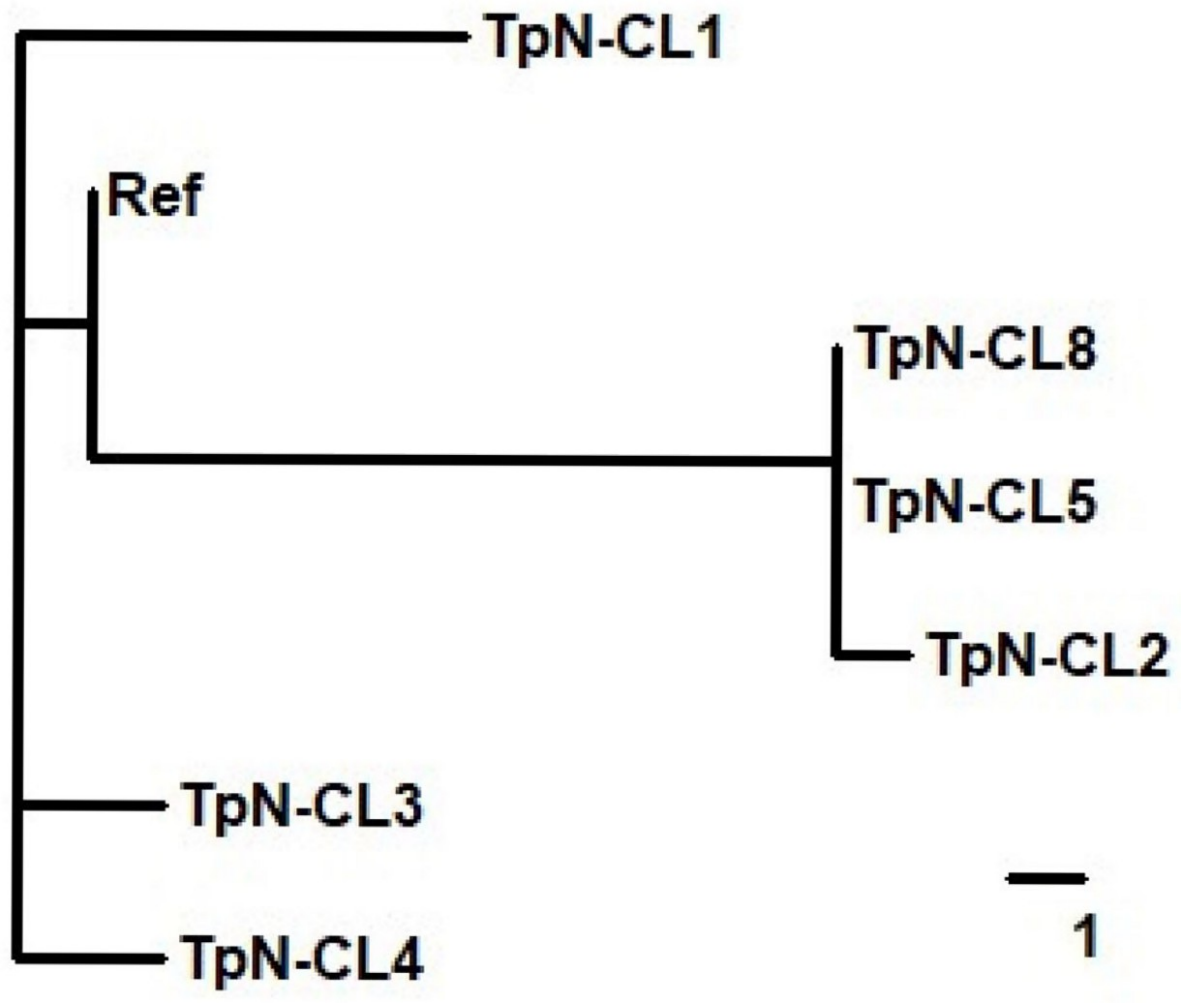
Cladogram showing the degree of genomic identity among the *T*. *pallidum* Nichols clones and the reference *T*. *pallidum* Nichols sequence NC_021490.2. The diagram is based on the 24 SNV and indel sites in Table 2 that have only two genotypes (e.g. T or C, or 1 or 2 repeat sequences); only *TP0470*, which has a variable number of repeat sequences, and the VR differences observed in *tprK* are excluded. One difference is equivalent to the horizontal distance shown by the reference bar in the lower right corner. The results indicate the close overall sequence identity of TpN-CL2, TpN-CL5, and TpN-CL8, the relatedness of TpN-CL3 and TpN-CL4, and the relatively frequent sequence differences in TpN-CL1 as compared to the other clones.

Examination of the sequences of the six clones revealed the presence of both distinct and shared genotypes. The overall pattern of relatedness was examined using a cladogram (**Fig 8**) that utilized the sequence features that exhibited a binary characteristic (e.g. SNV sites that have two different nucleotides, and indels in which only two arrangements were observed). Clones TpN-CL5 and TpN-CL8 were nearly identical, with identity at all of the 20 SNV sites and 4 of 5 of the indel sites (**Table 2**); the sole difference was the presence of 18 of the 24-nt repeats in TpN-CL5 and 16 repeats in TpN-CL8. Another difference was the presence of rare *tprK* variants in TpN-CL5 as opposed to the lack of detected variants in TpN-CL8. TpN-CL2 was also identical to TpN-CL8, except for a one nucleotide difference (T vs. C) at the reference position 700635. The difference is within the open reading frame of *TP0639*, encoding Mcp3, a methyl-accepting chemotaxis protein; it results in a T439I conversion in the predicted protein sequence. This mutation was found only in TpN-CL2 and is not present in the other *T*. *pallidum* preparations sequenced in this study or in other available genomic sequences; therefore, it likely represents a rare point mutation present in this clone.

The clones TpN-CL3 and TpN-CL4 were also highly similar, sharing identity at 19 of the 23 SNV and indel sites in **Table 2**. One of the four differences was at nt 810772 site in *TP0746*, which encodes the glycolytic pathway enzyme pyruvate phosphate dikinase (Ppdk). TpN-CL3 had SNVs at two locations relative to TpN-CL4 and the other clones sequenced. One of these differences is at nt 307493 within the ORF of *TP0292*, encoding an OmpF homologue. This A→G substitution results in an Q261R conversion and is not found in other *T*. *pallidum* genomes. A C→T point mutation is also located at nt 884280, 10 nt upstream of the start codon for *TP0814*, encoding thioredoxin-disulfide reductase (TxrB); there is no apparent promoter element or ribosome binding site in that region. This nucleotide substitution was present in a small proportion (2 to 3%) of IVA_d368 and IVA_d473 reads, but in a high proportion of IVB_d538 reads (98%). A search of all *T*. *pallidum* genomic sequences available in NCBI did not yield a single genome containing this C→T substitution. Two SNVs were present in *TP0972*, encoding the high affinity Fe2+/Pb2+ permease Ftr1; one in TpN-CL3 at the reference nt 1057375 results in a synonymous mutation (V165V), whereas the other found in TpN clones 2, 5, and 8 would cause a M244I conservative mutation.

### Potential functional correlates of heterogeneities

The availability of clonal populations with different mutations permits the evaluation of potential effects of these genetic differences on the biology and pathogenesis of *T*. *pallidum*, as summarized in **Table 3**. Many of the SNVs and indels are in intergenic regions, so their effects (except for those in potential promoter/operator regions) are difficult to predict; therefore these are excluded from this consideration. However, several are within open reading frames and are thus of interest to examine.

**Table 3:** Potential biological effects of *T*. *pallidum* Nichols sequence heterogeneities.

Ref. location^a^	Mutation type	Ref. genotype	TpN clone(s) differing from reference genotype	Nucleotide differences(s)	Amino acid effects	Gene	Product	Potential effect
7179	SNV	T	CL2, CL5, CL8: C	**T**AG → **C**AG	Stop56Q	*TP0006*	HP^b^	Change from truncated to full-length product
49361	polyG/C	10 Gs	CL1, CL2, CL4, CL5, CL8: 11 Gs	3’ end frameshift	C-terminus difference	*TP0040*	Methyl-accepting chemotaxis protein 1 (Mcp1)	Change in C-terminus may affect chemotaxis
59894	SNV	T	CL2, CL5, CL8: C	**T**CT → **C**CT	P104P (no change)	*TP0051*	peptide chain release factor RF1 (PrfA)	Synonymous mutation; no effect. Heterogeneous in Tp rabbit, *in vitro* cultures
148537–149740	Indel	No indel	CL1: deletion	1,204 nt deletion	--	*TP0126a*, *TP0126c*	HPs; *tprK* sequence donor site	Deletion of a subset of *tprK* antigenic variation donor sites
150149	polyG/C	10 Gs	CL3, CL4: 11 Gs	Mid-gene frameshift	C-terminus difference (aa 118–229)	*TP0127*	DUF2715 domain-containing protein	Major protein sequence change; effect unknown. Alteration also present in TpN Clone E and 4 other strains
156833	polyG/C	9 Gs	CL2: 11 Gs CL5, CL8: 10 Gs	3’ end frameshift	Affects AA 257–283	*TP0135*	HP	Clones have different ORFs annotated that do not include the polyG/C region
228259	SNV	A	CL2, CL5, CL8: C	GA**A** → GA**C**	E46D	*TP0222*	Hypothetical protein	Conservative replacement; no known effect
307493	SNV	A	CL3: G	C**A**G →C**G**G	Q261R	*TP0292*	OOP family OmpA-OmpF porin	Effect not known
463184	SNV	C	CL2, CL5, CL8: T	C**C**T → C**T**T	P497L	*TP0433*	Acidic Repeat Protein (Arp)	Nonconservative replacement; effect not known
498840	Indel	17 24-nt repeats	Varied number of 24-nt repeats	Repeat encoding 8 aa sequence	EARRKEAE	*TP0470*	HP	Varied length of predicted alpha-helical region
500905	SNV	G	CL2, CL5, CL8: A	**G**AT → **A**AT	D357N	*TP0471*	Hypothetical protein with TPR domain	Unknown. SNV also present in TpN_Rabbit, *in vitro* cultures but not present in other *T*. *pallidum* genome sequences
523669	SNV	A	CL2, CL5, CL8: C	A**A**A →A**C**A	K404T	*TP0488*	Methyl-accepting chemotaxis protein 2 (Mcp2)	Potential effect on chemotaxis
534222	SNV	C	No clones; Tp171103 d538 only	**C**GT→**T**GT	R250C	*TP0497*	Rod shape determining protein MreB	Cell shape determinant; no obvious morphologic differences observed
536766	SNV	G	CL1: A	**G**CC→**A**CC	A296T	*TP0500*	Penicillin-binding protein 2 (MrdA)	D-alanyl-D-alanyl transpeptidase; possible effect on peptidoglycan assembly
635418	SNV	T	CL1: G	T→G (promoter region)	--	*TP0548*	HP (promoter region)	Possible effect on promoter activity
671436	polyG/C	9 Cs	CL1, CL2, CL5, CL8: 10 Cs	5’ extension of *TP0617* ORF	N-terminus elongation	IGR *TP0617*, *TP0618*	DUF2715 domain-containing protein	Annotated as pseudogene in clones with 10 Cs, but encodes elongated *TP0617*
700635	SNV	C	CL2: T	ACC→ATC	T429I	*TP0639*	Methyl-accepting chemotaxis protein 3 (Mcp3)	Potential effect on chemotaxis
810772	SNV	G	CL2: A	**G**CA→**A**CA	A869T	*TP0746*	Pyruvate, phosphate dikinase Ppdk	Potential effect on glycolytic pathway
811414	SNV	G	CL1: A	**G**GG→**A**GG	P655S	*TP0746*	Pyruvate, phosphate dikinase Ppdk	Potential effect on glycolytic pathway
874548	SNV	A	CL1: G	G**A**G→G**G**G	E160G	*TP0804*	sn-glycerol-3-phosphate ABC transporter UgpC	Potential effect on glycerol-3-phosphate transport
884280	SNV	C	CL3: T	C→T	--	*TP0814*	Thioredoxin-disulfide reductase TxrB (promoter region)	Potential effect on transcription of *txrB* gene
974054	Indel (duplication)	CGTG	CL1, CL3, CL4: CGTGCGTG CL2, CL5, CL8: CGTG	CGTG**CGTG**→CGTG	M1M (No change)	*TP0893*	Ribosome maturation factor RimP	4 nucleotide relative insertion. Start codon is maintained (GUG), but spacing to promoter, RBS affected
975954	SNV	G	CL1: T	TGG→TGT	W496C^**b**^	*TP0897*	Antigenic variation protein TprK	SNV located outside of *tprK* variable regions, effect not known
1055553	polyG/C	9 Gs	CL2, CL5, CL8: 8 Gs	5’ end frameshift	N-terminal frameshift	*TP0969*	Potential outer membrane efflux protein	Truncated protein product; possible outer membrane permeability effect
1057089	SNV	C	CL2, CL5, CL8: T	AT**G**→ATT	M244I	*TP0972*	High-affinity Fe2+/Pb2+ permease Ftr1	Conservative mutation: effect not known
1057375	SNV	C	CL3: T	G**C**G → G**T**G	V165V	*TP0972*	High-affinity Fe2+/Pb2+ permease Ftr1	Synonymous mutation; no effect

a Reference location and reference genotype refers to the *T*. *pallidum* Nichols genome sequence (GenBank No. NC_021490.2) as previously determined by Pětrošová et al. [36].

b HP = hypothetical protein.

An interesting example is the heterogeneity that exists within *TP0006*, which encodes a Domain of Unknown Function (DUF) 3798 domain-containing protein with a length of 415 aa. In the Nichols strain, the nucleotide T at the beginning of codon 56 results in the stop codon (TAG) and a truncated, 55 aa predicted product; a C at this location encodes Gln and yields a full-length product. The TpNRabbit preparation and most of the *in vitro* culture specimens contained a mixture of these two genotypes, with the rabbit propagated *T*. *pallidum* having 68% T and 32% C; in contrast, the TpN-IVB_d538 culture had 99% T and 1% C. In the TpN clonal populations, clones 1, 3, and 4 had T at this position, whereas clones 2, 5, and 8 had C. The finding that strains and clones with either genotype are fully infectious in rabbits (**Table 1**) and multiply at similar rates *in vitro* (**Fig 2**) indicates that the full-length protein is not essential for pathogenesis or *in vitro* growth. However, full-length TP0006 is encoded in the genomes of over 200 *T*. *pallidum* strains, including strains in the *pertenue* and *endemicum* subspecies. Homologs are also present in many spirochetes and other bacteria, indicating that this conserved protein family has an important (as yet undefined) function. A paralog (TP0013; 48% identical, 65% similar) is also present in the *T*. *pallidum* genome and may have a function that overlaps with that of TP0006.

Three of the mutations are within the genes of the methyl-accepting chemotaxis proteins Mcp1, Mcp2 and Mcp3 (**Table 3**). Mcps are chemoreceptors that sense the presence of chemoattractants or chemorepellents and begin the cascade leading to chemotactic motility; four are encoded in the *T*. *pallidum* genome. A polyG/C expansion from 10 Gs to 11 Gs in *mcp1* of all but one of the TpN clones is predicted to result in a change of the C-terminus of the resulting protein. The Mcp2 mutation (K404T) in TpN clones 2, 5, and 8 is part of the predicted second sensor domain (cd12912; aa 325–426). TpN-CL2 has an additional mutation in the gene encoding Mcp3 (T429I) is located in between the sensor domain and the Tar region (COG0840; aa 431–845) involved in CheW interactions and signal transduction. Further studies would be needed to determine whether these mutations have any effect on Mcp activity.

The predicted structure of TP0470 (as determined by the program Robetta (https://robetta.bakerlab.org) indicates that the TPR domain forms a tight cluster of short alpha helices near the N terminus, while the 8 aa repeats result in a single long alpha helix at the C-terminal portion of the protein (**Fig 4D**). As mentioned previously, *TP0746* encodes pyruvate, phosphate dikinase, Ppdk. In *T*. *pallidum*, this enzyme most likely catalyzes the conversion of phosphoenol pyruvate, AMP, and diphosphate to pyruvate and ATP. The C→T substitution in TpN-CL4 (**Table 3**) results in an Ala→Thr transition at aa 869 in the 885-aa predicted protein. This polymorphism was present in a small proportion (1%) of the *T*. *pallidum* population in our rabbit-propagated preparation, but at a high frequency in some of the *in vitro* cultures (77% in IGA_d368 and 83% in IGA_d473). This genotype was not found in any of the other *T*. *pallidum* genome sequences in the NCBI database. Therefore, it appears to be a point mutation specific to Nichols strain subpopulations, as represented by TpN-CL4. Another unique *ppdk* mutation was present in TpN-CL1 with a single nucleotide replacement resulting in a P655S substitution. Both of these substitutions are located in the predicted Ppdk PEP-binding TIM barrel domain (PF02896, aa 521–874) and thus potentially could have an effect on this activity.

TpN-CL1 contained another mutation in *TP0500* encoding a D-alanyl-D-alanyl transpeptidase (MrdA) involved in cell elongation. The A296T substitution is located in the transpeptidase domain 26 aa from the predicted active site (_322_PASTFK_327_, with serine being the catalytic residue). The same clone had a E160G substitution in *TP0804*, encoding UgpC, annotated as a glycerol-6-phosphate ABC transporter ATP-binding protein; the mutation is in a well-conserved region in which most bacteria have an aspartate at that position.

Finally, *TP0972*, encoding a putative high-affinity Fe2+/Pb2+ permease (Ftr1) has a SNV in TpN-CL2, TpN-CL5, and TpN-CL8 that would cause a M244I change at the protein level. While this is a conservative amino acid substitution, it may affect the transport function of this predicted protein.

## Discussion

In this study, we demonstrated that clones of *T*. *pallidum* strains can be isolated using a limiting dilution approach. Positive cultures were obtained with inocula as low as 2 or 0.5 organisms per well, with on average 12% of these wells being positive. These results suggest that the cloning efficiency for this system is considerably less than 100%, so that wells containing more than one *T*. *pallidum* may also yield clones. Prolonged incubation times of ~21 days were required to detect organisms by either microscopy or qPCR, consistent with the long doubling time (typically 30 to 44 hours) for this organism. Genomic sequence analysis confirmed that the clones were indeed isogenic and exhibited heterogeneities from one another. The genomic sequences from rabbit-propagated and *in vitro* cultured *T*. *pallidum* clearly contained the heterogeneities present in the clone sequences; it is likely that many more genetically distinct subpopulations are present in rabbit- and *in vitro* culture-derived strains. The comparison of these sequences indicates that the Nichols strain originated from a single *T*. *pallidum* isolate and then diverged through micro-evolution during the century of propagation following its isolation, as discussed more thoroughly later. Finally, *T*. *pallidum* clones such as those described in this article will likely be useful as starting material in future genetic analyses, including those employing random or targeted mutagenesis.

Detailed study of six clones from the Nichols strain revealed that they had indistinguishable morphology and *in vitro* growth characteristics. It was also found that the TpN clones examined for infectivity had very similar properties during intradermal infection of rabbits, similar to those observed with the uncloned Nichols population [27,30]. The only meaningful differences occurred at the lowest inoculation dosage tested, 10 *T*. *pallidum* per site. In this case, TpN-CL1 and TpN-CL4 resulted in infection at all four sites, whereas TpN-CL2 and TpN-CL8 had 2/4 and 1/4 sites infected, respectively. Further investigation would be needed to determine if these results indicate a reproducible difference in infectivity. However, the consistent infection at higher dosages indicate that the clones were all highly infectious, despite the presence of genetic heterogeneities. We did not examine the ability of the pathogen to disseminate from the site of inoculation in this study.

Intrastrain heterogeneity has been reported previously in the Nichols strain and other *T*. *pallidum* strains [9,23,36,42]. Pětrošová et al. [36] re-sequenced the genomes of the Nichols and SS14 strains of *T*. *pallidum* subsp. *pallidum* using Illumina and other procedures and then analyzed the sequences for intrastrain heterogeneity. They found that the resulting SS14 genome sequence had heterogeneity at 46 single nucleotide variation sites. However, no intrastrain heterogeneity was detected in the re-sequenced Nichols strain sequence using their criteria. In a later study by Čejková et al. [23], five of the SNVs (at nt 7179, 59894, 228259, 500905, and 635418; Table 2) found in our analysis were reported. The previously reported 1204 nt region [9] absent in the original Nichols genome sequence [7] was present in the re-sequenced genome [36]. This region corresponds precisely with the 1204 nt sequence that exhibited intrastrain heterogeneity in our sequencing of the rabbit-propagated *T*. *pallidum*; it was also absent from TpN-CL1 but present in all the other clones. (It should be noted that the Fraser et al. [7] and Pětrošová et al. [36] Nichols strain genomic sequences and the studies reported here utilized the strain propagated in our laboratory [commonly called “Nichols Houston”] as the source of DNA for sequencing.)

The heterogeneity observed within the Nichols strain (and between the resulting clones) was potentially due to two possible mechanisms. One possibility is that the patient from whom the Nichols strain was isolated was actually infected with multiple different *T*. *pallidum* lineages. However, comparison to all available *T*. *pallidum* genomic sequences revealed that the SNVs observed in the Nichols uncloned and cloned populations (**Table 2**) were found only in Nichols-derived sequences. None of the same point mutations were detected in the >280 other *T*. *pallidum* genomic sequences currently available in the NCBI database; a few examples of concordance were found in the case of repeat sequence indels. Thus the most plausible mechanism is that the Nichols strain was isogenic upon isolation from the patient in 1912 [43] and has accumulated additional point mutations, indels, and other rearrangements during its 110-year period of passage in rabbits and *in vitro* cultures. This latter explanation is consistent with numerous prior studies showing the genetic drift that occurs in clones of bacteria during propagation, as exemplified by two recent studies with *Helicobacter pylori* [44,45]. In our study, it is not possible to estimate a meaningful mutation rate in *T*. *pallidum* using the number of mutations, genome size and length of time, given the unknown number of variables (e.g. use of long-term latent infection of rabbits and ‘suspended animation” of the strain during freezing) that would affect the number of generations during this prolonged period.

The simple cladogram shown in **Fig 8** indicates that clones TpN-CL2, TpN-CL5, and TpN-CL8 are most closely related, i.e., likely share a common lineage. TpN-CL3 and TpN-CL4 are similarly clustered, with TpN-CL1 being the most different from the others. Most of the indel differences and SNVs were shared by more than one clone. However, some of the SNVs were unique to one clone (**Table 2**). TpN-CL1 had the most unique SNVs [5], present at the reference nt locations 53766, 635418, 811414, 874548, and 975954. TpN-CL3 had two unique SNVs, as does TpN-CL4; TpN-CL2 had a single unique SNV. Most of these SNVs were detected in the TpN_Rabbit or *in vitro* culture samples, indicating that they were ‘pre-existing’ in these populations. The observed heterogeneity in the number of 24 nt repeats in TpN-CL4 at nt 498847 (**Table 2**) likely occurred after clone isolation, since the other indel and SNV sites are homogeneous (indicating clonality).

To examine the degree of heterogeneity in uncloned Nichols strain populations, we also performed genomic sequencing of the rabbit propagated strain as well as four *in vitro* cultures isolated from two long-term lineages at different time points. We found that each of these preparations had different patterns of heterogeneity at the SNV sites identified in the TpN clones (**Table 2**). The availability of the homogeneous clone sequences in our study permitted a targeted determination of heterogeneity at frequencies as low as 1% in the SNVs identified among the clones. We found such heterogeneities in the rabbit propagated population at 9 of the 20 SNVs identified in the clones (**Table 2**). In each case, there were two alternative nucleotides that matched those present in the clones, with the proportion of the less frequent nucleotide ranging from 1% to 48% at the nine sites with detectable heterogeneity (see TpN_Rabbit column in **Table 2**). Similar results were obtained with the four indels for which quantitative data could be obtained with the rabbit propagated organisms. One could imagine that the SNV heterogeneities in the rabbit derived population could simply represent a certain mixture of the six clones sequenced (e.g. 40% TpN-CL4, etc.). However, our analysis indicated that these six clonotypes represent only a small part of the diversity present in the original (rabbit propagated) population. The *in vitro* culture genomic sequences had different genotype proportions than the rabbit derived population from which they were initiated (**Table 2**), indicating that ‘genetic drift’ has occurred during the period of *in vitro* culture. In general, the IVA culture lineage has become more like TpN-CL4 in its genotype, whereas the IVB lineage has become more similar to the group represented by TpN clones 2, 5, and 8. Given that the IVA and IVB culture conditions were the same, it is likely that these changes represent evolutionary drift rather than a selective process. This divergence may have been fueled by the occurrence of culture crises during which the number of *T*. *pallidum* decreased [27,30]; these crises could have represented bottlenecks leading to either random or directed selection of subpopulations.

Many prior studies had noted the existence of *T*. *pallidum* heterogeneity with regard to indels and the varied length of repeat sequence regions (reviewed in [15,46]. In **Fig 4A and 4B**, we propose models for how the observed 1204-nt and 67-nt deletions result from crossover events between the flanking direct repeat sequences at each site; flanking repeats for the 1204-nt deletion had been reported previously [14]. The expansion and contraction of the number of repeat elements (**Fig 4C–4E**) most likely arises from slipped strand mispairing, which has long been known to be an important genetic variation mechanism in bacteria as well as eukaryotes [47–49].

Availability of clonal populations also permitted a more detailed examination of aspects of *tprK* recombinations. The variable regions of the clones had far fewer variants than did the rabbit-propagated or *in vitro* culture populations (**Figs 5 and 6**). No *tprK* variants were detected in clones TpN-CL2 and TpN-CL8, whereas TpN-CL1, TpN-CL3, and TpN-CL5 had very limited variation. Although TpN-CL4 had a higher proportion of variants in VR7, the number of variants observed in each VR ranged from 2 to 4. In comparison, the number of VR variants in the rabbit and *in vitro* culture populations generally ranged from 2 to 20 (median = 11); this result excludes TpN_IVB_d538, which appeared to be more clonal, based on the limited heterogeneity in both *tprK* and other loci (**Table 2**). The full-length *tprK* sequences derived from PacBio CCS data indicate a high degree of relatedness among the *tprK* variants detected within each clone (**Fig 7**). In some cases, there were rabbit or *in vitro* culture *tprK* sequences that clustered with the sequences from an individual clone. The results were similar to other recent full-length *tprK* analyses [12,13], with the additional information provided by the availability of clonal populations. Overall, the findings are consistent with the occurrence of *tprK* variation at low rates during *in vitro* propagation of the clones, in agreement with the recent report by Lin et al. [10]. The near homogeneity of both the *tprK* sequences and other genetic loci among TpN-CL2, TpN-CL5, and TpN-CL8 indicate that these clones were all derived from a common clonotype. Comparison of *tprK* sequences in a clone at a series of time points during *in vitro* culture would be needed to determine a rate of *tprK* recombination at each VR under these conditions.

The genetic polymorphisms within the six Nichols strain clones characterized in this study are likely to have some biologic effects. No obvious variations in morphology and growth patterns and only minor potential differences in rabbit infectivity (**Table 1**) were noted among the clones. However, it is possible that the effects are subtle but could have selective advantages or disadvantages over a number of generations; these could be examined by more sensitive methods such as competition studies either *in vivo* (during infection) or *in vitro*. Most SNVs within genes were nonsynonymous, a trend that was noted in prior studies of *T*. *pallidum* heterogeneity [14,23,36]. It was surprising that genetic variations were identified in the three genes encoding Mcps, proteins important in detecting chemotactic signals and thus directed motility (**Table 3**). The mutation in *mcp2* was an SNV found in TpN-CL2, TpN-CL5, and TpN-CL8, and another SNV in *mcp2* was present only in TpN-CL2. The *mcp1*-associated polyG/C expansion located in 5 of the clones causes a change in the C-terminal region of the protein, which may affect interactions with the signal transduction protein CheW. This observation is similar to the prior description by Pinto et al. [11] of *mcp* heterogeneity among a large number of patient isolates. A SNV in the gene encoding Ppdk (which catalyzes the conversion of pyruvate enol phosphate to pyruvate) may affect cellular metabolic activities, while those in other genes may impact cell structure (*mreB*, *mrdA*) and transport (*ompA-ompF* porin homolog, *ugpC*) (**Table 3**). These scattered mutations among closely related clones exemplify the random nature of microevolution: genetic changes in *T*. *pallidum* would be expected to occur continuously with only a small proportion eventually becoming ‘fixed’ in the population through positive selection.

This study demonstrates the feasibility of generating clones in *T*. *pallidum* Nichols and other strains. The procedure is likely applicable to all strains that can be propagated in the *in vitro* culture system, which currently includes Nichols-like and SS14-like *T*. *pallidum* subsp. *pallidum* isolates and *T*. *pallidum* subsp. *endemicum* strains. The availability of isogenic populations through cloning will be useful in future genetic studies of these organisms. The feasibility of performing targeted mutagenesis has already been demonstrated by Romeis et al. [34] through the replacement of the *tprA* pseudogene in *T*. *pallidum* subsp. *pallidum* SS14 with a kanamycin resistance cassette. It is likely that random mutagenesis will also be possible using *mariner*-mediated transposition or similar approaches, as has been performed previously with the spirochetes *Borrelia burgdorferi* [50] and *Leptospira interrogans* [51]. Moreover, the limiting dilution method can be utilized to derive clonal isolates of mutants (and complemented mutants), which will be important in ascertaining genotype-phenotype correlations. If needed, two rounds of limiting dilution could be performed to assure the isogenic nature of the clone, similar to the approach taken previously in the derivation of multiple *B*. *burgdorferi* B31 clones [52]. Finally, the ability to isolate *T*. *pallidum* from small inocula supports the possibility that new strains can be isolated directly from patient (or infected animal) specimens using the *in vitro* culture system.

## Materials and methods

### Ethics statement

All procedures involving rabbits were reviewed and approved by the Animal Welfare Committee of the University of Texas Health Science Center at Houston under protocol AWC-20-0150.

### Tissue culture

All reagents were purchased from Sigma-Aldrich unless otherwise indicated. Sf1Ep cottontail rabbit epithelial cells (NBL-11; ATCC^®^ CCL-68^TM^) were obtained from the American Type Culture Collection, Rockville, MD. Sf1Ep cells are supplied as a secondary cell culture with limited growth potential. Stocks between passage 30 and 70 were used. Cells were maintained in Eagle’s MEM with non-essential amino acids, L-glutamine, sodium pyruvate, and 10% heat-inactivated FBS [53] at 37°C in air with 5% CO_2_ [27,31].

### Bacteria

*Treponema pallidum* subspecies *pallidum* Nichols, initially isolated from the cerebrospinal fluid of a neurosyphilis patient in Baltimore, Maryland, U.S.A. in 1912 [43], was obtained from J. N. Miller at the UCLA Geffen School of Medicine. The UW249B strain was isolated in 2004 from the blood of an untreated syphilis patient in Seattle, Washington, U.S.A. and was the kind gift of L. C. Tantalo, S. K. Sahi, and C. M. Marra (University of Washington School of Medicine). The Mexico A strain was isolated in 1953 from a male patient with primary syphilis and was provided by David Cox and David Šmajs. *T*. *pallidum* strains were maintained by intratesticular passage in male New Zealand white rabbits (3–4 kg) as previously described [25,27,31]. Rabbit cells were removed by centrifugation at 100 x g for 7 minutes. The *T*. *pallidum* suspension was supplemented with 15% (v/v) sterile glycerol and stored in 1 ml aliquots at -80˚C. *In vitro* cultured *T*. *pallidum* were frozen in TpCM-2 medium supplemented with 15% glycerol.

### *In vitro* cultivation of *T*. *pallidum*

*In vitro* cultivation of *T*. *pallidum* has been described previously in detail [27,31]. Briefly, the *T*. *pallidum* were grown in co-culture with Sf1Ep cells in *T*. *pallidum* culture medium-2 (TpCM-2) at 34°C in an atmosphere of 1.5% O_2_:5% CO_2_:93.5% N_2_. Pre-equilibration conditions, *T*. *pallidum* inoculation, incubation, passage by trypsinization, and quantitation of *T*. *pallidum* were performed as described [27,31]. Standard cultures were carried out with 6-well tissue culture plates with 1 x 10^5^ Sf1Ep cells and 4 ml TpCM-2 per well and incubation for 7 days prior to subculture. Data for the ongoing long-term in vitro lineages IVA and IVB (initiated October 20, 2017 and November 3, 2017, respectively) were tabulated on July 30, 2022.

### Growth of *T*. *pallidum* in 96-well plates

The procedure for cloning *T*. *pallidum* by limiting dilution was as described [31]. In brief, low-evaporation 96-well tissue culture plates (Corning Falcon®, 353072) were seeded with 1000–3000 Sf1Ep cells per well one day prior to the start of each experiment. On the day the experiment began, the plates with rinsed with TpCM-2, refilled with fresh TpCM-2, and pre-equilibrated in the low oxygen incubator as described above. Following equilibration, the plates were inoculated with the indicated numbers of *T*. *pallidum* in a final volume of 200 to 250 μl TpCM-2. The plates were removed once a week from the low oxygen incubator and “fed” by removing 100 μl of medium and replacing it with 100 μl of fresh TpCM-2. If significant evaporation was observed, extra TpCM-2 was added to bring the volume back to 200–250 μl. For plates seeded at low density (<8 *T*. *pallidum* per well), 50–100 μl supernatants from each well were removed and “passed” to a freshly prepared 96-well plate after two weeks. Presence of *T*. *pallidum* in the culture supernatants was assessed by darkfield microscopy or by polA qPCR.

Potential clones were expanded by trypsinizing the positive wells and transferring the trypsinized culture to progressively larger culture dishes. The culture dishes were prepared the day prior to passage as described above. Culture wells were seeded with 2 x 10^4^ (12-well), 1 x 10^4^ (24-well) or 6 x 10^3^ (48-well) Sf1Ep cells. After one week of incubation, a sample of the culture supernatant was examined by darkfield microscopy to determine if the density of *T*. *pallidum* was sufficient for passage to a larger culture vessel. Culture with low numbers of *T*. *pallidum* were fed by replacing half the medium with fresh TpCM-2 after one week. Following two weeks in culture, the Sf1Ep monolayer would begin to fail, so potential clones were typically transferred to a fresh Sf1Ep monolayer after two weeks regardless of *T*. *pallidum* density.

### DNA preparation

*T*. *pallidum* DNA was isolated using the Qiagen DNeasy Blood and Tissue kit per manufacturer’s instructions for gram negative bacteria. Trypsinized cultures (4–50 ml.) of *in vitro* grown *T*. *pallidum* were centrifuged at 700 x G for 7 minutes to remove Sf1Ep cells. *T*. *pallidum* were then pelleted by centrifugation at 12,000 x G for 10 minutes at room temperature; the resulting pellets were used for DNA isolation. DNA from *T*. *pallidum* propagated in rabbits was isolated from frozen extracts used for inoculating *in vitro* cultures using the same method.

### PCR

Short regions of *TP0222* and *TP0488* were generated by PCR using primers that flank the areas of sequence heterogeneity. *TP0222* was amplified using the primers 6217sense (5’-GCACTATTGTGGGGTATGGTG-3’) and 6218antisense (5’-CAATGCGTTCACACGCTGCC-3’) while *TP0488* was amplified using primers 6219sense (5’-CACGTACGCGTCCGTCGCAG-3’) and 6220antisense (5’-CGCAAGCAGGTAGTTCTGC-3’). DNA isolated as above was used as template. PCR fragments were purified with a Qiagen Qiaquick PCR purification Kit and sequenced by the Sanger method (GeneWiz/Azenta Life Sciences, South Plainfield, NJ) using the same primers used for amplification.

### Quantitative PCR

qPCR was performed using iQ^TM^ SYBR green supermix on a C1000 Touch Thermal Cycler (Bio-Rad). Reaction volumes of 20 μl were used and 2 μl of supernatant from *T*. *pallidum* cultures were used as template. The primers used were targeted to the *T*. *pallidum* DNA Polymerase I gene (*polA*, *TP0105*): 6037TP1 (5’-CAGGATCCGGCATATGTCC-3’ AND 6037TP2 (5’-AAGTGTGAGCGTCTCATCATTCC-3’). The program consisted of 95°C for 2 min, followed 39 cycles of 95°C for 5 s and 60°C for 30 s. All samples were examined in triplicate technical replicates, and standard curves using purified *T*. *pallidum* DNA were performed for each plate and had linear regression coefficients of determination (R^2^) of ≥0.98.

### Infectivity studies

The infectivity of cultured *T*. *pallidum* was determined by injecting serial dilutions of the sample intradermally into the shaved backs of two rabbits. Dilutions were performed in TpCM-2 and each dilution was inoculated at duplicate sites on each rabbit. The inoculation sites were shaved and examined daily for 45 days for the occurrence of erythema and induration, which together constitute lesion development. Needle aspirates of representative lesions were examined by darkfield microscopy for the presence of motile treponemes indicating active treponemal infection. Rabbits were provided antibiotic-free food and water, housed at 16–18˚C and shaved daily throughout the course of the experiment. Median infectious dose (ID50) values were determined by the method of Reed and Münsch [54].

### Genome sequencing

Extracted DNA was prepared for sequencing using the Illumina Nextera XT DNA Library Prep Kit and sequenced on an Illumina MiSeq with the v3 kit using 2x300 paired end reads. All sequencing reads were trimmed to remove adapter contamination, low quality regions, and low complexity regions using Trimmomatic v0.36 [55] and single nucleotide polymorphisms, insertions, and deletions were identified using Snippy v4.3.6 (https://github.com/tseemann/snippy). The trimmed reads were aligned to the *T*. *pallidum* subsp. *pallidum* Nichols genome (Genbank: NC_021490.2) using Bowtie2 v2.3.4.1 [56] and genetic variants were confirmed by visual inspection. The reference sequence NC_021490.2 contained regions of uncertain sequence (Ns) in *tprK* as well as additional regions of heterogeneity specific to each of the new genome sequences. Therefore, the genomic sequences were refined through an iterative process by replacing the reference template sequence with ones containing variant regions corresponding to each of the new sequences and then realigning the trimmed reads to this modified template using Minimap2 [57].

PacBio circular consensus sequencing (CCS) was performed (Azenta) on DNA samples from the same preparations of rabbit-propagated *T*. *pallidum* and TpN clones 1, 2, 3, 4, 5 and 8 as utilized in the Illumina sequencing. Insufficient DNA was available for PacBio sequencing of the *T*. *pallidum in vitro* cultures examined by Illumina sequencing. PacBio CCS analysis was performed on two additional samples from *in vitro* cultures (Tp171020_d1288 and Tp171103_d1274). The PacBio data was utilized to resolve regions of ambiguity (e.g. repeat regions and long indels) and to examine full-length *tprK* regions.

The coverage was variable within each sequence, due in part to differences in the amount of *T*. *pallidum* and contaminating rabbit DNA in each preparation. The complete genome sequences for the clones were established with a minimum combined coverage of three Illumina or PacBio reads. The *tprK* sequences in the clone GenBank entries represent the most common sequence, in the clones where heterogeneity existed. The TpNRabbit, IVA, and IVB sequences were not posted, because of the high number of intrastrain heterogeneities present. Instead, the reader is referred to Table 2, which has a full listing of these heterogeneities and the proportion of the genotypes at each location. Some indels and repeat regions were not resolved in the uncloned rabbit- and *in vitro* culture- derived preparations because of the lack of long-read PacBio data.

A more detailed examination of the *tprK* region, homopolymeric tracts (> 8 nt), indels, and repeat regions was performed. These analyses included areas encompassing the individual *tprK* variable regions, corresponding to the following regions of GenBank NC_021490.2: 9777190–977225 (VR1), 796707–976772 (VR2), 976566–976625 (VR3), 976485–976532 (VR4), 976359–976442 (VR5), 976224–976277 (VR6), and 976070–976148 (VR7). For each genomic dataset, the samtools view function [58] was utilized to extract Illumina reads in the regions of each of these features. The reads were then aligned using MAFFT [59] (https://mafft.cbrc.jp/alignment/server/) and trimmed to the region of interest using BioEdit (Tom Hall, developer); truncated sequences were discarded. The number of reads corresponding to each heterogeneous variant in a given region was then tabulated using Microsoft Excel. The same process was employed to capture and align reads containing the full-length sequences of the intact *tprK* ORF in the PacBio datasets. In that case, alignments with the corresponding Illumina reads were used to correct the single nucleotide indel sequencing errors that sometimes occur in PacBio CCS data. For **Fig 7**, the corresponding sequences were realigned using MAFFT, and the corresponding Newick file was generated using the UPGMA parameters available on the MAFFT webserver. The resulting Newick data were converted into tree format using the iTol server (https://itol.embl.de/).

## Supporting information

S1 Fig Alignment of the full-length *tprK* nucleotide sequences obtained by PacBio sequencing. (PDF)

S2 Fig Alignment of the full-length *tprK* amino acid sequences obtained by PacBio sequencing. (PDF)

S1 Table Genomic sequencing information for rabbit-derived samples, in vitro culture-derived samples, and in vitro culture Treponema pallidum subsp. pallidum Nichols. (PDF)

S2 Table Polynucleotide length differences in T. pallidum Nichols clones. (XLSX)
