# Supplementary material for: Clonal isolates of Treponema pallidum subsp. pallidum Nichols provide evidence for the occurrence of microevolution during experimental rabbit infection and in vitro culture
Source: PLoS One. 2023 Mar 14;18(3):e0281187. doi: 10.1371/journal.pone.0281187 (PMC10013896; doi:10.1371/journal.pone.0281187)
Supplement: S1 Fig — (PDF) [file pone.0281187.s001.pdf]

Fig. S1. Alignment of the full-length *tprK* nucleotide sequences obtained by PacBio sequencing. Each read is labeled with the sample name (e.g. TpNRabbit) and the read identification number; these correspond to the identifiers in Fig. 7. The reference sequence (CP004010.2) is included for comparison. The locations of the variable regions (VR1 through VR7) are indicated in blue.

```
NC_021490.2_tprK           1 ATGATTGACCCATCTGCCACTTCCCGGTATGGTTCCCCACGTTTAGTTAGTAATGGTTTTCGGCATCGGAGAAAAGTGGTGTATCAGCGGGTAGGGCACA
TpNRabbit/11797378         1 ATGATTGACCCATCTGCCACTTCCCGGTATGGTTCCCCACGTTTAGTTAGTAATGGTTTTCGGCATCGGAGAAAAGTGGTGTATCAGCGGGTAGGGCACA
TpNRabbit/49086983         1 ATGATTGACCCATCTGCCACTTCCCGGTATGGTTCCCCACGTTTAGTTAGTAATGGTTTTCGGCATCGGAGAAAAGTGGTGTATCAGCGGGTAGGGCACA
TpNCL1/74908282            1 ATGATTGACCCATCTGCCACTTCCCGGTATGGTTCCCCACGTTTAGTTAGTAATGGTTTTCGGCATCGGAGAAAAGTGGTGTATCAGCGGGTAGGGCACA
TpNRabbit/74646114         1 ATGATTGACCCATCTGCCACTTCCCGGTATGGTTCCCCACGTTTAGTTAGTAATGGTTTTCGGCATCGGAGAAAAGTGGTGTATCAGCGGGTAGGGCACA
TpNIVA_d1288/58000085      1 ATGATTGACCCATCTGCCACTTCCCGGTATGGTTCCCCACGTTTAGTTAGTAATGGTTTTCGGCATCGGAGAAAAGTGGTGTATCAGCGGGTAGGGCACA
TpNCL1/67830753            1 ATGATTGACCCATCTGCCACTTCCCGGTATGGTTCCCCACGTTTAGTTAGTAATGGTTTTCGGCATCGGAGAAAAGTGGTGTATCAGCGGGTAGGGCACA
TpNRabbit/37356380         1 ATGATTGACCCATCTGCCACTTCCCGGTATGGTTCCCCACGTTTAGTTAGTAATGGTTTTCGGCATCGGAGAAAAGTGGTGTATCAGCGGGTAGGGCACA
TpNIVA_d1288/62194580      1 ATGATTGACCCATCTGCCACTTCCCGGTATGGTTCCCCACGTTTAGTTAGTAATGGTTTTCGGCATCGGAGAAAAGTGGTGTATCAGCGGGTAGGGCACA
TpNIVB_d1274/56361402      1 ATGATTGACCCATCTGCCACTTCCCGGTATGGTTCCCCACGTTTAGTTAGTAATGGTTTTCGGCATCGGAGAAAAGTGGTGTATCAGCGGGTAGGGCACA
TpNIVB_d1274/57606550      1 ATGATTGACCCATCTGCCACTTCCCGGTATGGTTCCCCACGTTTAGTTAGTAATGGTTTTCGGCATCGGAGAAAAGTGGTGTATCAGCGGGTAGGGCACA
TpNCL2/71434737            1 ATGATTGACCCATCTGCCACTTCCCGGTATGGTTCCCCACGTTTAGTTAGTAATGGTTTTCGGCATCGGAGAAAAGTGGTGTATCAGCGGGTAGGGCACA
TpNCL5/23396777            1 ATGATTGACCCATCTGCCACTTCCCGGTATGGTTCCCCACGTTTAGTTAGTAATGGTTTTCGGCATCGGAGAAAAGTGGTGTATCAGCGGGTAGGGCACA
TpNCL8/37093850            1 ATGATTGACCCATCTGCCACTTCCCGGTATGGTTCCCCACGTTTAGTTAGTAATGGTTTTCGGCATCGGAGAAAAGTGGTGTATCAGCGGGTAGGGCACA
TpNIVB_d1274/39452938      1 ATGATTGACCCATCTGCCACTTCCCGGTATGGTTCCCCACGTTTAGTTAGTAATGGTTTTCGGCATCGGAGAAAAGTGGTGTATCAGCGGGTAGGGCACA
TpNIVA_d1288/9765540       1 ATGATTGACCCATCTGCCACTTCCCGGTATGGTTCCCCACGTTTAGTTAGTAATGGTTTTCGGCATCGGAGAAAAGTGGTGTATCAGCGGGTAGGGCACA
TpNCL3/13042505            1 ATGATTGACCCATCTGCCACTTCCCGGTATGGTTCCCCACGTTTAGTTAGTAATGGTTTTCGGCATCGGAGAAAAGTGGTGTATCAGCGGGTAGGGCACA
TpNCL3/50332092            1 ATGATTGACCCATCTGCCACTTCCCGGTATGGTTCCCCACGTTTAGTTAGTAATGGTTTTCGGCATCGGAGAAAAGTGGTGTATCAGCGGGTAGGGCACA
TpNCL3/24642061            1 ATGATTGACCCATCTGCCACTTCCCGGTATGGTTCCCCACGTTTAGTTAGTAATGGTTTTCGGCATCGGAGAAAAGTGGTGTATCAGCGGGTAGGGCACA
TpNCL3/25952592            1 ATGATTGACCCATCTGCCACTTCCCGGTATGGTTCCCCACGTTTAGTTAGTAATGGTTTTCGGCATCGGAGAAAAGTGGTGTATCAGCGGGTAGGGCACA
TpNCL3/61080088            1 ATGATTGACCCATCTGCCACTTCCCGGTATGGTTCCCCACGTTTAGTTAGTAATGGTTTTCGGCATCGGAGAAAAGTGGTGTATCAGCGGGTAGGGCACA
TpNCL3/39322209            1 ATGATTGACCCATCTGCCACTTCCCGGTATGGTTCCCCACGTTTAGTTAGTAATGGTTTTCGGCATCGGAGAAAAGTGGTGTATCAGCGGGTAGGGCACA
TpNCL3/73007774            1 ATGATTGACCCATCTGCCACTTCCCGGTATGGTTCCCCACGTTTAGTTAGTAATGGTTTTCGGCATCGGAGAAAAGTGGTGTATCAGCGGGTAGGGCACA
TpNCL3/46924011            1 ATGATTGACCCATCTGCCACTTCCCGGTATGGTTCCCCACGTTTAGTTAGTAATGGTTTTCGGCATCGGAGAAAAGTGGTGTATCAGCGGGTAGGGCACA
TpNCL3/34013629            1 ATGATTGACCCATCTGCCACTTCCCGGTATGGTTCCCCACGTTTAGTTAGTAATGGTTTTCGGCATCGGAGAAAAGTGGTGTATCAGCGGGTAGGGCACA
TpNCL4/16253724            1 ATGATTGACCCATCTGCCACTTCCCGGTATGGTTCCCCACGTTTAGTTAGTAATGGTTTTCGGCATCGGAGAAAAGTGGTGTATCAGCGGGTAGGGCACA
TpNCL4/67830320            1 ATGATTGACCCATCTGCCACTTCCCGGTATGGTTCCCCACGTTTAGTTAGTAATGGTTTTCGGCATCGGAGAAAAGTGGTGTATCAGCGGGTAGGGCACA
TpNCL4/49087362            1 ATGATTGACCCATCTGCCACTTCCCGGTATGGTTCCCCACGTTTAGTTAGTAATGGTTTTCGGCATCGGAGAAAAGTGGTGTATCAGCGGGTAGGGCACA
TpNCL4/56558172            1 ATGATTGACCCATCTGCCACTTCCCGGTATGGTTCCCCACGTTTAGTTAGTAATGGTTTTCGGCATCGGAGAAAAGTGGTGTATCAGCGGGTAGGGCACA
TpNCL4/24117649            1 ATGATTGACCCATCTGCCACTTCCCGGTATGGTTCCCCACGTTTAGTTAGTAATGGTTTTCGGCATCGGAGAAAAGTGGTGTATCAGCGGGTAGGGCACA
TpNCL4/74580654            1 ATGATTGACCCATCTGCCACTTCCCGGTATGGTTCCCCACGTTTAGTTAGTAATGGTTTTCGGCATCGGAGAAAAGTGGTGTATCAGCGGGTAGGGCACA
TpNCL4/29164148            1 ATGATTGACCCATCTGCCACTTCCCGGTATGGTTCCCCACGTTTAGTTAGTAATGGTTTTCGGCATCGGAGAAAAGTGGTGTATCAGCGGGTAGGGCACA
TpNIVA_d1288/44237532      1 ATGATTGACCCATCTGCCACTTCCCGGTATGGTTCCCCACGTTTAGTTAGTAATGGTTTTCGGCATCGGAGAAAAGTGGTGTATCAGCGGGTAGGGCACA
TpNIVA_d1288/52757423      1 ATGATTGACCCATCTGCCACTTCCCGGTATGGTTCCCCACGTTTAGTTAGTAATGGTTTTCGGCATCGGAGAAAAGTGGTGTATCAGCGGGTAGGGCACA
TpNRabbit/68420554         1 ATGATTGACCCATCTGCCACTTCCCGGTATGGTTCCCCACGTTTAGTTAGTAATGGTTTTCGGCATCGGAGAAAAGTGGTGTATCAGCGGGTAGGGCACA
TpNIVA_d1288/72286947      1 ATGATTGACCCATCTGCCACTTCCCGGTATGGTTCCCCACGTTTAGTTAGTAATGGTTTTCGGCATCGGAGAAAAGTGGTGTATCAGCGGGTAGGGCACA
TpNIVA_d1288/15598261      1 ATGATTGACCCATCTGCCACTTCCCGGTATGGTTCCCCACGTTTAGTTAGTAATGGTTTTCGGCATCGGAGAAAAGTGGTGTATCAGCGGGTAGGGCACA
```

```
NC_021490.2_tprK        101 GGCGATTTTCTCTCATTTTCTTTTTCGTTGTGGTTCTGGGGCGGTCCCCGCGGCTGTGGGCTCAGGTTTCGTTCACCCCGGATATTGAAGGCTATGCGGA
TpNRabbit/11797378      101 GGCGATTTTCTCTCATTTTCTTTTTCGTTGTGGTTCTGGGGCGGTCCCCGCGGCTGTGGGCTCAGGTTTCGTTCACCCCGGATATTGAAGGCTATGCGGA
TpNRabbit/49086983      101 GGCGATTTTCTCTCATTTTCTTTTTCGTTGTGGTTCTGGGGCGGTCCCCGCGGCTGTGGGCTCAGGTTTCGTTCACCCCGGATATTGAAGGCTATGCGGA
TpNCL1/74908282         101 GGCGATTTTCTCTCATTTTCTTTTTCGTTGTGGTTCTGGGGCGGTCCCCGCGGCTGTGGGCTCAGGTTTCGTTCACCCCGGATATTGAAGGCTATGCGGA
TpNRabbit/74646114      101 GGCGATTTTCTCTCATTTTCTTTTTCGTTGTGGTTCTGGGGCGGTCCCCGCGGCTGTGGGCTCAGGTTTCGTTCACCCCGGATATTGAAGGCTATGCGGA
TpNIVA_d1288/58000085   101 GGCGATTTTCTCTCATTTTCTTTTTCGTTGTGGTTCTGGGGCGGTCCCCGCGGCTGTGGGCTCAGGTTTCGTTCACCCCGGATATTGAAGGCTATGCGGA
TpNCL1/67830753         101 GGCGATTTTCTCTCATTTTCTTTTTCGTTGTGGTTCTGGGGCGGTCCCCGCGGCTGTGGGCTCAGGTTTCGTTCACCCCGGATATTGAAGGCTATGCGGA
TpNRabbit/37356380      101 GGCGATTTTCTCTCATTTTCTTTTTCGTTGTGGTTCTGGGGCGGTCCCCGCGGCTGTGGGCTCAGGTTTCGTTCACCCCGGATATTGAAGGCTATGCGGA
TpNIVA_d1288/62194580   101 GGCGATTTTCTCTCATTTTCTTTTTCGTTGTGGTTCTGGGGCGGTCCCCGCGGCTGTGGGCTCAGGTTTCGTTCACCCCGGATATTGAAGGCTATGCGGA
TpNIVB_d1274/56361402   101 GGCGATTTTCTCTCATTTTCTTTTTCGTTGTGGTTCTGGGGCGGTCCCCGCGGCTGTGGGCTCAGGTTTCGTTCACCCCGGATATTGAAGGCTATGCGGA
TpNIVB_d1274/57606550   101 GGCGATTTTCTCTCATTTTCTTTTTCGTTGTGGTTCTGGGGCGGTCCCCGCGGCTGTGGGCTCAGGTTTCGTTCACCCCGGATATTGAAGGCTATGCGGA
TpNCL2/71434737         101 GGCGATTTTCTCTCATTTTCTTTTTCGTTGTGGTTCTGGGGCGGTCCCCGCGGCTGTGGGCTCAGGTTTCGTTCACCCCGGATATTGAAGGCTATGCGGA
TpNCL5/23396777         101 GGCGATTTTCTCTCATTTTCTTTTTCGTTGTGGTTCTGGGGCGGTCCCCGCGGCTGTGGGCTCAGGTTTCGTTCACCCCGGATATTGAAGGCTATGCGGA
TpNCL8/37093850         101 GGCGATTTTCTCTCATTTTCTTTTTCGTTGTGGTTCTGGGGCGGTCCCCGCGGCTGTGGGCTCAGGTTTCGTTCACCCCGGATATTGAAGGCTATGCGGA
TpNIVB_d1274/39452938   101 GGCGATTTTCTCTCATTTTCTTTTTCGTTGTGGTTCTGGGGCGGTCCCCGCGGCTGTGGGCTCAGGTTTCGTTCACCCCGGATATTGAAGGCTATGCGGA
TpNIVA_d1288/9765540    101 GGCGATTTTCTCTCATTTTCTTTTTCGTTGTGGTTCTGGGGCGGTCCCCGCGGCTGTGGGCTCAGGTTTCGTTCACCCCGGATATTGAAGGCTATGCGGA
TpNCL3/13042505         101 GGCGATTTTCTCTCATTTTCTTTTTCGTTGTGGTTCTGGGGCGGTCCCCGCGGCTGTGGGCTCAGGTTTCGTTCACCCCGGATATTGAAGGCTATGCGGA
TpNCL3/50332092         101 GGCGATTTTCTCTCATTTTCTTTTTCGTTGTGGTTCTGGGGCGGTCCCCGCGGCTGTGGGCTCAGGTTTCGTTCACCCCGGATATTGAAGGCTATGCGGA
TpNCL3/24642061         101 GGCGATTTTCTCTCATTTTCTTTTTCGTTGTGGTTCTGGGGCGGTCCCCGCGGCTGTGGGCTCAGGTTTCGTTCACCCCGGATATTGAAGGCTATGCGGA
TpNCL3/25952592         101 GGCGATTTTCTCTCATTTTCTTTTTCGTTGTGGTTCTGGGGCGGTCCCCGCGGCTGTGGGCTCAGGTTTCGTTCACCCCGGATATTGAAGGCTATGCGGA
TpNCL3/61080088         101 GGCGATTTTCTCTCATTTTCTTTTTCGTTGTGGTTCTGGGGCGGTCCCCGCGGCTGTGGGCTCAGGTTTCGTTCACCCCGGATATTGAAGGCTATGCGGA
TpNCL3/39322209         101 GGCGATTTTCTCTCATTTTCTTTTTCGTTGTGGTTCTGGGGCGGTCCCCGCGGCTGTGGGCTCAGGTTTCGTTCACCCCGGATATTGAAGGCTATGCGGA
TpNCL3/73007774         101 GGCGATTTTCTCTCATTTTCTTTTTCGTTGTGGTTCTGGGGCGGTCCCCGCGGCTGTGGGCTCAGGTTTCGTTCACCCCGGATATTGAAGGCTATGCGGA
TpNCL3/46924011         101 GGCGATTTTCTCTCATTTTCTTTTTCGTTGTGGTTCTGGGGCGGTCCCCGCGGCTGTGGGCTCAGGTTTCGTTCACCCCGGATATTGAAGGCTATGCGGA
TpNCL3/34013629         101 GGCGATTTTCTCTCATTTTCTTTTTCGTTGTGGTTCTGGGGCGGTCCCCGCGGCTGTGGGCTCAGGTTTCGTTCACCCCGGATATTGAAGGCTATGCGGA
TpNCL4/16253724         101 GGCGATTTTCTCTCATTTTCTTTTTCGTTGTGGTTCTGGGGCGGTCCCCGCGGCTGTGGGCTCAGGTTTCGTTCACCCCGGATATTGAAGGCTATGCGGA
TpNCL4/67830320         101 GGCGATTTTCTCTCATTTTCTTTTTCGTTGTGGTTCTGGGGCGGTCCCCGCGGCTGTGGGCTCAGGTTTCGTTCACCCCGGATATTGAAGGCTATGCGGA
TpNCL4/49087362         101 GGCGATTTTCTCTCATTTTCTTTTTCGTTGTGGTTCTGGGGCGGTCCCCGCGGCTGTGGGCTCAGGTTTCGTTCACCCCGGATATTGAAGGCTATGCGGA
TpNCL4/56558172         101 GGCGATTTTCTCTCATTTTCTTTTTCGTTGTGGTTCTGGGGCGGTCCCCGCGGCTGTGGGCTCAGGTTTCGTTCACCCCGGATATTGAAGGCTATGCGGA
TpNCL4/24117649         101 GGCGATTTTCTCTCATTTTCTTTTTCGTTGTGGTTCTGGGGCGGTCCCCGCGGCTGTGGGCTCAGGTTTCGTTCACCCCGGATATTGAAGGCTATGCGGA
TpNCL4/74580654         101 GGCGATTTTCTCTCATTTTCTTTTTCGTTGTGGTTCTGGGGCGGTCCCCGCGGCTGTGGGCTCAGGTTTCGTTCACCCCGGATATTGAAGGCTATGCGGA
TpNCL4/29164148         101 GGCGATTTTCTCTCATTTTCTTTTTCGTTGTGGTTCTGGGGCGGTCCCCGCGGCTGTGGGCTCAGGTTTCGTTCACCCCGGATATTGAAGGCTATGCGGA
TpNIVA_d1288/44237532   101 GGCGATTTTCTCTCATTTTCTTTTTCGTTGTGGTTCTGGGGCGGTCCCCGCGGCTGTGGGCTCAGGTTTCGTTCACCCCGGATATTGAAGGCTATGCGGA
TpNIVA_d1288/52757423   101 GGCGATTTTCTCTCATTTTCTTTTTCGTTGTGGTTCTGGGGCGGTCCCCGCGGCTGTGGGCTCAGGTTTCGTTCACCCCGGATATTGAAGGCTATGCGGA
TpNRabbit/68420554      101 GGCGATTTTCTCTCATTTTCTTTTTCGTTGTGGTTCTGGGGCGGTCCCCGCGGCTGTGGGCTCAGGTTTCGTTCACCCCGGATATTGAAGGCTATGCGGA
TpNIVA_d1288/72286947   101 GGCGATTTTCTCTCATTTTCTTTTTCGTTGTGGTTCTGGGGCGGTCCCCGCGGCTGTGGGCTCAGGTTTCGTTCACCCCGGATATTGAAGGCTATGCGGA
TpNIVA_d1288/15598261   101 GGCGATTTTCTCTCATTTTCTTTTTCGTTGTGGTTCTGGGGCGGTCCCCGCGGCTGTGGGCTCAGGTTTCGTTCACCCCGGATATTGAAGGCTATGCGGA
```

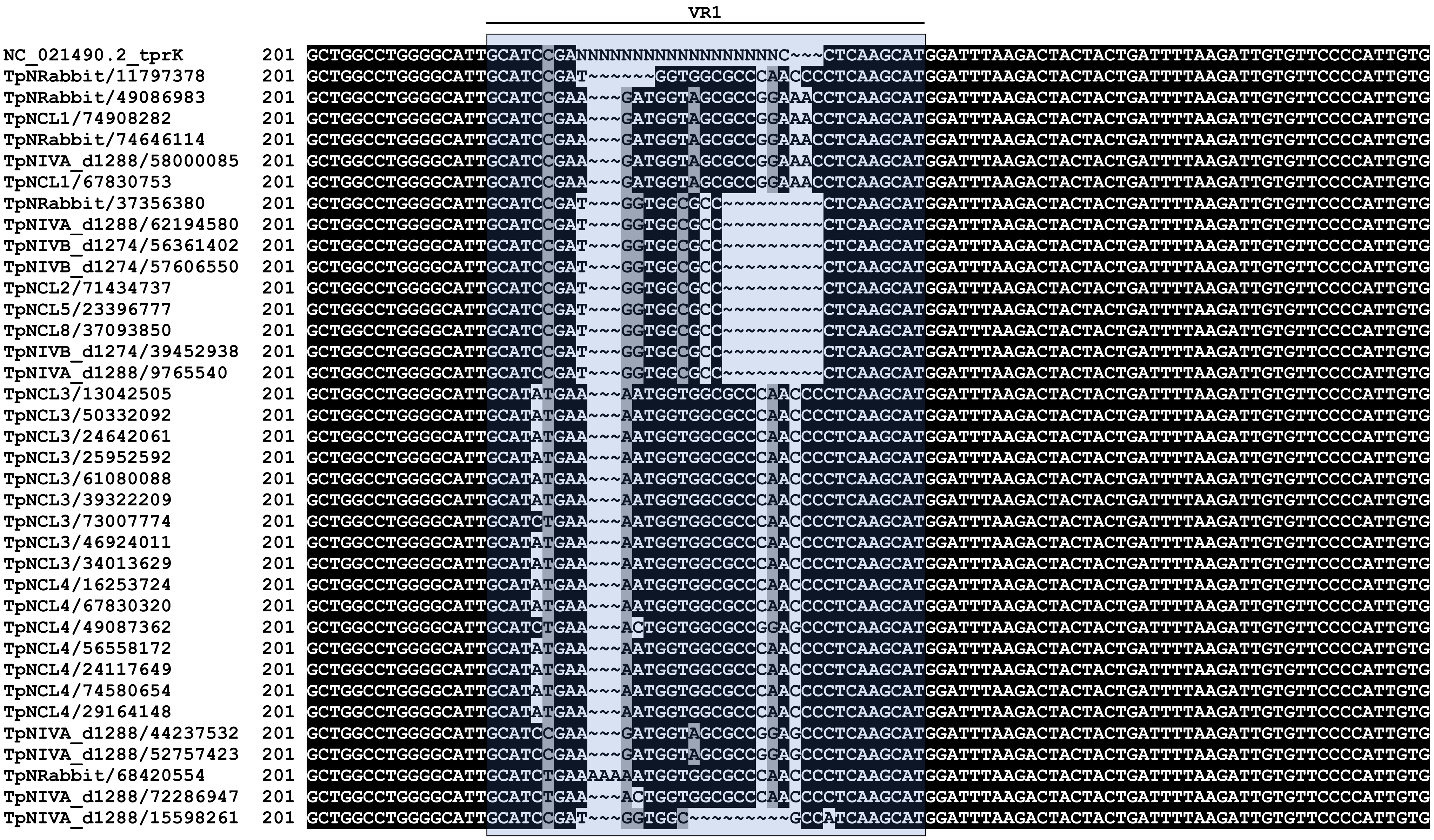
VR1
NC_021490.2_tprK 201 GCTGGCCTGGGGCATTGCATCCGANNNNNNNNNNNNNNNNNNC~~~CTCAAGCATGGATTTAAGACTACTACTGATTTTAAGATTGTGTTCCCCATTGTG
TpNRabbit/11797378 201 GCTGGCCTGGGGCATTGCATCCGAT~~~~~~GGTGGCGCCCAACCCCTCAAGCATGGATTTAAGACTACTACTGATTTTAAGATTGTGTTCCCCATTGTG
TpNRabbit/49086983 201 GCTGGCCTGGGGCATTGCATCCGAA~~~GATGGTAGCGCCGGAAACCTCAAGCATGGATTTAAGACTACTACTGATTTTAAGATTGTGTTCCCCATTGTG
TpNCL1/74908282 201 GCTGGCCTGGGGCATTGCATCCGAA~~~GATGGTAGCGCCGGAAACCTCAAGCATGGATTTAAGACTACTACTGATTTTAAGATTGTGTTCCCCATTGTG
TpNRabbit/74646114 201 GCTGGCCTGGGGCATTGCATCCGAA~~~GATGGTAGCGCCGGAAACCTCAAGCATGGATTTAAGACTACTACTGATTTTAAGATTGTGTTCCCCATTGTG
TpNIVA_d1288/58000085 201 GCTGGCCTGGGGCATTGCATCCGAA~~~GATGGTAGCGCCGGAAACCTCAAGCATGGATTTAAGACTACTACTGATTTTAAGATTGTGTTCCCCATTGTG
TpNCL1/67830753 201 GCTGGCCTGGGGCATTGCATCCGAA~~~GATGGTAGCGCCGGAAACCTCAAGCATGGATTTAAGACTACTACTGATTTTAAGATTGTGTTCCCCATTGTG
TpNRabbit/37356380 201 GCTGGCCTGGGGCATTGCATCCGAT~~~GGTGGCGCC~~~~~~~~~CTCAAGCATGGATTTAAGACTACTACTGATTTTAAGATTGTGTTCCCCATTGTG
TpNIVA_d1288/62194580 201 GCTGGCCTGGGGCATTGCATCCGAT~~~GGTGGCGCC~~~~~~~~~CTCAAGCATGGATTTAAGACTACTACTGATTTTAAGATTGTGTTCCCCATTGTG
TpNIVB_d1274/56361402 201 GCTGGCCTGGGGCATTGCATCCGAT~~~GGTGGCGCC~~~~~~~~~CTCAAGCATGGATTTAAGACTACTACTGATTTTAAGATTGTGTTCCCCATTGTG
TpNIVB_d1274/57606550 201 GCTGGCCTGGGGCATTGCATCCGAT~~~GGTGGCGCC~~~~~~~~~CTCAAGCATGGATTTAAGACTACTACTGATTTTAAGATTGTGTTCCCCATTGTG
TpNCL2/71434737 201 GCTGGCCTGGGGCATTGCATCCGAT~~~GGTGGCGCC~~~~~~~~~CTCAAGCATGGATTTAAGACTACTACTGATTTTAAGATTGTGTTCCCCATTGTG
TpNCL5/23396777 201 GCTGGCCTGGGGCATTGCATCCGAT~~~GGTGGCGCC~~~~~~~~~CTCAAGCATGGATTTAAGACTACTACTGATTTTAAGATTGTGTTCCCCATTGTG
TpNCL8/37093850 201 GCTGGCCTGGGGCATTGCATCCGAT~~~GGTGGCGCC~~~~~~~~~CTCAAGCATGGATTTAAGACTACTACTGATTTTAAGATTGTGTTCCCCATTGTG
TpNIVB_d1274/39452938 201 GCTGGCCTGGGGCATTGCATCCGAT~~~GGTGGCGCC~~~~~~~~~CTCAAGCATGGATTTAAGACTACTACTGATTTTAAGATTGTGTTCCCCATTGTG
TpNIVA_d1288/9765540 201 GCTGGCCTGGGGCATTGCATCCGAT~~~GGTGGCGCC~~~~~~~~~CTCAAGCATGGATTTAAGACTACTACTGATTTTAAGATTGTGTTCCCCATTGTG
TpNCL3/13042505 201 GCTGGCCTGGGGCATTGCATATGAA~~~AATGGTGGCGCCCAACCCCTCAAGCATGGATTTAAGACTACTACTGATTTTAAGATTGTGTTCCCCATTGTG
TpNCL3/50332092 201 GCTGGCCTGGGGCATTGCATATGAA~~~AATGGTGGCGCCCAACCCCTCAAGCATGGATTTAAGACTACTACTGATTTTAAGATTGTGTTCCCCATTGTG
TpNCL3/24642061 201 GCTGGCCTGGGGCATTGCATATGAA~~~AATGGTGGCGCCCAACCCCTCAAGCATGGATTTAAGACTACTACTGATTTTAAGATTGTGTTCCCCATTGTG
TpNCL3/25952592 201 GCTGGCCTGGGGCATTGCATATGAA~~~AATGGTGGCGCCCAACCCCTCAAGCATGGATTTAAGACTACTACTGATTTTAAGATTGTGTTCCCCATTGTG
TpNCL3/61080088 201 GCTGGCCTGGGGCATTGCATATGAA~~~AATGGTGGCGCCCAACCCCTCAAGCATGGATTTAAGACTACTACTGATTTTAAGATTGTGTTCCCCATTGTG
TpNCL3/39322209 201 GCTGGCCTGGGGCATTGCATATGAA~~~AATGGTGGCGCCCAACCCCTCAAGCATGGATTTAAGACTACTACTGATTTTAAGATTGTGTTCCCCATTGTG
TpNCL3/73007774 201 GCTGGCCTGGGGCATTGCATCTGAA~~~AATGGTGGCGCCCAACCCCTCAAGCATGGATTTAAGACTACTACTGATTTTAAGATTGTGTTCCCCATTGTG
TpNCL3/46924011 201 GCTGGCCTGGGGCATTGCATATGAA~~~AATGGTGGCGCCCAACCCCTCAAGCATGGATTTAAGACTACTACTGATTTTAAGATTGTGTTCCCCATTGTG
TpNCL3/34013629 201 GCTGGCCTGGGGCATTGCATATGAA~~~AATGGTGGCGCCCAACCCCTCAAGCATGGATTTAAGACTACTACTGATTTTAAGATTGTGTTCCCCATTGTG
TpNCL4/16253724 201 GCTGGCCTGGGGCATTGCATATGAA~~~AATGGTGGCGCCCAACCCCTCAAGCATGGATTTAAGACTACTACTGATTTTAAGATTGTGTTCCCCATTGTG
TpNCL4/67830320 201 GCTGGCCTGGGGCATTGCATATGAA~~~AATGGTGGCGCCCAACCCCTCAAGCATGGATTTAAGACTACTACTGATTTTAAGATTGTGTTCCCCATTGTG
TpNCL4/49087362 201 GCTGGCCTGGGGCATTGCATCTGAA~~~ACTGGTGGCGCCGGAGCCCTCAAGCATGGATTTAAGACTACTACTGATTTTAAGATTGTGTTCCCCATTGTG
TpNCL4/56558172 201 GCTGGCCTGGGGCATTGCATATGAA~~~AATGGTGGCGCCCAACCCCTCAAGCATGGATTTAAGACTACTACTGATTTTAAGATTGTGTTCCCCATTGTG
TpNCL4/24117649 201 GCTGGCCTGGGGCATTGCATATGAA~~~AATGGTGGCGCCCAACCCCTCAAGCATGGATTTAAGACTACTACTGATTTTAAGATTGTGTTCCCCATTGTG
TpNCL4/74580654 201 GCTGGCCTGGGGCATTGCATATGAA~~~AATGGTGGCGCCCAACCCCTCAAGCATGGATTTAAGACTACTACTGATTTTAAGATTGTGTTCCCCATTGTG
TpNCL4/29164148 201 GCTGGCCTGGGGCATTGCATATGAA~~~AATGGTGGCGCCCAACCCCTCAAGCATGGATTTAAGACTACTACTGATTTTAAGATTGTGTTCCCCATTGTG
TpNIVA_d1288/44237532 201 GCTGGCCTGGGGCATTGCATCCGAA~~~GATGGTAGCGCCGGAGCCCTCAAGCATGGATTTAAGACTACTACTGATTTTAAGATTGTGTTCCCCATTGTG
TpNIVA_d1288/52757423 201 GCTGGCCTGGGGCATTGCATCCGAA~~~GATGGTAGCGCCGGAGCCCTCAAGCATGGATTTAAGACTACTACTGATTTTAAGATTGTGTTCCCCATTGTG
TpNRabbit/68420554 201 GCTGGCCTGGGGCATTGCATCTGAAAAAAATGGTGGCGCCCAACCCCTCAAGCATGGATTTAAGACTACTACTGATTTTAAGATTGTGTTCCCCATTGTG
TpNIVA_d1288/72286947 201 GCTGGCCTGGGGCATTGCATCTGAA~~~ACTGGTGGCGCCCAACCCCTCAAGCATGGATTTAAGACTACTACTGATTTTAAGATTGTGTTCCCCATTGTG
TpNIVA_d1288/15598261 201 GCTGGCCTGGGGCATTGCATCCGAT~~~GGTGGC~~~~~~~~~GCCATCAAGCATGGATTTAAGACTACTACTGATTTTAAGATTGTGTTCCCCATTGTG

NC_021490.2_tprK	298	GCAAAGAAGGATTTCAAGTACCGCGGTGAGGGGAATGTCTATGCGGAAATTAATGTTAAAGCGTTGAAGTTGAGTTTAGAGTCAAATGGTGGAGCAAAGT
TpNRabbit/11797378	295	GCAAAGAAGGATTTCAAGTACCGCGGTGAGGGGAATGTCTATGCGGAAATTAATGTTAAAGCGTTGAAGTTGAGTTTAGAGTCAAATGGTGGAGCAAAGT
TpNRabbit/49086983	298	GCAAAGAAGGATTTCAAGTACCGCGGTGAGGGGAATGTCTATGCGGAAATTAATGTTAAAGCGTTGAAGTTGAGTTTAGAGTCAAATGGTGGAGCAAAGT
TpNCL1/74908282	298	GCAAAGAAGGATTTCAAGTACCGCGGTGAGGGGAATGTCTATGCGGAAATTAATGTTAAAGCGTTGAAGTTGAGTTTAGAGTCAAATGGTGGAGCAAAGT
TpNRabbit/74646114	298	GCAAAGAAGGATTTCAAGTACCGCGGTGAGGGGAATGTCTATGCGGAAATTAATGTTAAAGCGTTGAAGTTGAGTTTAGAGTCAAATGGTGGAGCAAAGT
TpNIVA_d1288/58000085	298	GCAAAGAAGGATTTCAAGTACCGCGGTGAGGGGAATGTCTATGCGGAAATTAATGTTAAAGCGTTGAAGTTGAGTTTAGAGTCAAATGGTGGAGCAAAGT
TpNCL1/67830753	298	GCAAAGAAGGATTTCAAGTACCGCGGTGAGGGGAATGTCTATGCGGAAATTAATGTTAAAGCGTTGAAGTTGAGTTTAGAGTCAAATGGTGGAGCAAAGT
TpNRabbit/37356380	289	GCAAAGAAGGATTTCAAGTACCGCGGTGAGGGGAATGTCTATGCGGAAATTAATGTTAAAGCGTTGAAGTTGAGTTTAGAGTCAAATGGTGGAGCAAAGT
TpNIVA_d1288/62194580	289	GCAAAGAAGGATTTCAAGTACCGCGGTGAGGGGAATGTCTATGCGGAAATTAATGTTAAAGCGTTGAAGTTGAGTTTAGAGTCAAATGGTGGAGCAAAGT
TpNIVB_d1274/56361402	289	GCAAAGAAGGATTTCAAGTACCGCGGTGAGGGGAATGTCTATGCGGAAATTAATGTTAAAGCGTTGAAGTTGAGTTTAGAGTCAAATGGTGGAGCAAAGT
TpNIVB_d1274/57606550	289	GCAAAGAAGGATTTCAAGTACCGCGGTGAGGGGAATGTCTATGCGGAAATTAATGTTAAAGCGTTGAAGTTGAGTTTAGAGTCAAATGGTGGAGCAAAGT
TpNCL2/71434737	289	GCAAAGAAGGATTTCAAGTACCGCGGTGAGGGGAATGTCTATGCGGAAATTAATGTTAAAGCGTTGAAGTTGAGTTTAGAGTCAAATGGTGGAGCAAAGT
TpNCL5/23396777	289	GCAAAGAAGGATTTCAAGTACCGCGGTGAGGGGAATGTCTATGCGGAAATTAATGTTAAAGCGTTGAAGTTGAGTTTAGAGTCAAATGGTGGAGCAAAGT
TpNCL8/37093850	289	GCAAAGAAGGATTTCAAGTACCGCGGTGAGGGGAATGTCTATGCGGAAATTAATGTTAAAGCGTTGAAGTTGAGTTTAGAGTCAAATGGTGGAGCAAAGT
TpNIVB_d1274/39452938	289	GCAAAGAAGGATTTCAAGTACCGCGGTGAGGGGAATGTCTATGCGGAAATTAATGTTAAAGCGTTGAAGTTGAGTTTAGAGTCAAATGGTGGAGCAAAGT
TpNIVA_d1288/9765540	289	GCAAAGAAGGATTTCAAGTACCGCGGTGAGGGGAATGTCTATGCGGAAATTAATGTTAAAGCGTTGAAGTTGAGTTTAGAGTCAAATGGTGGAGCAAAGT
TpNCL3/13042505	298	GCAAAGAAGGATTTCAAGTACCGCGGTGAGGGGAATGTCTATGCGGAAATTAATGTTAAAGCGTTGAAGTTGAGTTTAGAGTCAAATGGTGGAGCAAAGT
TpNCL3/50332092	298	GCAAAGAAGGATTTCAAGTACCGCGGTGAGGGGAATGTCTATGCGGAAATTAATGTTAAAGCGTTGAAGTTGAGTTTAGAGTCAAATGGTGGAGCAAAGT
TpNCL3/24642061	298	GCAAAGAAGGATTTCAAGTACCGCGGTGAGGGGAATGTCTATGCGGAAATTAATGTTAAAGCGTTGAAGTTGAGTTTAGAGTCAAATGGTGGAGCAAAGT
TpNCL3/25952592	298	GCAAAGAAGGATTTCAAGTACCGCGGTGAGGGGAATGTCTATGCGGAAATTAATGTTAAAGCGTTGAAGTTGAGTTTAGAGTCAAATGGTGGAGCAAAGT
TpNCL3/61080088	298	GCAAAGAAGGATTTCAAGTACCGCGGTGAGGGGAATGTCTATGCGGAAATTAATGTTAAAGCGTTGAAGTTGAGTTTAGAGTCAAATGGTGGAGCAAAGT
TpNCL3/39322209	298	GCAAAGAAGGATTTCAAGTACCGCGGTGAGGGGAATGTCTATGCGGAAATTAATGTTAAAGCGTTGAAGTTGAGTTTAGAGTCAAATGGTGGAGCAAAGT
TpNCL3/73007774	298	GCAAAGAAGGATTTCAAGTACCGCGGTGAGGGGAATGTCTATGCGGAAATTAATGTTAAAGCGTTGAAGTTGAGTTTAGAGTCAAATGGTGGAGCAAAGT
TpNCL3/46924011	298	GCAAAGAAGGATTTCAAGTACCGCGGTGAGGGGAATGTCTATGCGGAAATTAATGTTAAAGCGTTGAAGTTGAGTTTAGAGTCAAATGGTGGAGCAAAGT
TpNCL3/34013629	298	GCAAAGAAGGATTTCAAGTACCGCGGTGAGGGGAATGTCTATGCGGAAATTAATGTTAAAGCGTTGAAGTTGAGTTTAGAGTCAAATGGTGGAGCAAAGT
TpNCL4/16253724	298	GCAAAGAAGGATTTCAAGTACCGCGGTGAGGGGAATGTCTATGCGGAAATTAATGTTAAAGCGTTGAAGTTGAGTTTAGAGTCAAATGGTGGAGCAAAGT
TpNCL4/67830320	298	GCAAAGAAGGATTTCAAGTACCGCGGTGAGGGGAATGTCTATGCGGAAATTAATGTTAAAGCGTTGAAGTTGAGTTTAGAGTCAAATGGTGGAGCAAAGT
TpNCL4/49087362	298	GCAAAGAAGGATTTCAAGTACCGCGGTGAGGGGAATGTCTATGCGGAAATTAATGTTAAAGCGTTGAAGTTGAGTTTAGAGTCAAATGGTGGAGCAAAGT
TpNCL4/56558172	298	GCAAAGAAGGATTTCAAGTACCGCGGTGAGGGGAATGTCTATGCGGAAATTAATGTTAAAGCGTTGAAGTTGAGTTTAGAGTCAAATGGTGGAGCAAAGT
TpNCL4/24117649	298	GCAAAGAAGGATTTCAAGTACCGCGGTGAGGGGAATGTCTATGCGGAAATTAATGTTAAAGCGTTGAAGTTGAGTTTAGAGTCAAATGGTGGAGCAAAGT
TpNCL4/74580654	298	GCAAAGAAGGATTTCAAGTACCGCGGTGAGGGGAATGTCTATGCGGAAATTAATGTTAAAGCGTTGAAGTTGAGTTTAGAGTCAAATGGTGGAGCAAAGT
TpNCL4/29164148	298	GCAAAGAAGGATTTCAAGTACCGCGGTGAGGGGAATGTCTATGCGGAAATTAATGTTAAAGCGTTGAAGTTGAGTTTAGAGTCAAATGGTGGAGCAAAGT
TpNIVA_d1288/44237532	298	GCAAAGAAGGATTTCAAGTACCGCGGTGAGGGGAATGTCTATGCGGAAATTAATGTTAAAGCGTTGAAGTTGAGTTTAGAGTCAAATGGTGGAGCAAAGT
TpNIVA_d1288/52757423	298	GCAAAGAAGGATTTCAAGTACCGCGGTGAGGGGAATGTCTATGCGGAAATTAATGTTAAAGCGTTGAAGTTGAGTTTAGAGTCAAATGGTGGAGCAAAGT
TpNRabbit/68420554	301	GCAAAGAAGGATTTCAAGTACCGCGGTGAGGGGAATGTCTATGCGGAAATTAATGTTAAAGCGTTGAAGTTGAGTTTAGAGTCAAATGGTGGAGCAAAGT
TpNIVA_d1288/72286947	298	GCAAAGAAGGATTTCAAGTACCGCGGTGAGGGGAATGTCTATGCGGAAATTAATGTTAAAGCGTTGAAGTTGAGTTTAGAGTCAAATGGTGGAGCAAAGT
TpNIVA_d1288/15598261	289	GCAAAGAAGGATTTCAAGTACCGCGGTGAGGGGAATGTCTATGCGGAAATTAATGTTAAAGCGTTGAAGTTGAGTTTAGAGTCAAATGGTGGAGCAAAGT

```
NC_021490.2_tprK        398 TTGACACGAAGGGTTCTGCAAAGACGATAGAGGCAACCCTGCACTGTTAT~~~GGGGCCTACCTGACCATTGGGAAGAATCCTGATTTTAAGTCAACGTT
TpNRabbit/11797378      395 TTGACACGAAGGGTTCTGCAAAGACGATAGAGGCAACCCTGCACTGTTAT~~~GGGGCCTACCTGACCATTGGGAAGAATCCTGATTTTAAGTCAACGTT
TpNRabbit/49086983      398 TTGACACGAAGGGTTCTGCAAAGACGATAGAGGCAACCCTGCACTGTTAT~~~GGGGCCTACCTGACCATTGGGAAGAATCCTGATTTTAAGTCAACGTT
TpNCL1/74908282         398 TTGACACGAAGGGTTCTGCAAAGACGATAGAGGCAACCCTGCACTGTTAT~~~GGGGCCTACCTGACCATTGGGAAGAATCCTGATTTTAAGTCAACGTT
TpNRabbit/74646114      398 TTGACACGAAGGGTTCTGCAAAGACGATAGAGGCAACCCTGCACTGTTAT~~~GGGGCCTACCTGACCATTGGGAAGAATCCTGATTTTAAGTCAACGTT
TpNIVA_d1288/58000085   398 TTGACACGAAGGGTTCTGCAAAGACGATAGAGGCAACCCTGCACTGTTAT~~~GGGGCCTACCTGACCATTGGGAAGAATCCTGATTTTAAGTCAACGTT
TpNCL1/67830753         398 TTGACACGAAGGGTTCTGCAAAGACGATAGAGGCAACCCTGCACTGTTAT~~~GGGGCCTACCTGACCATTGGGAAGAATCCTGATTTTAAGTCAACGTT
TpNRabbit/37356380      389 TTGACACGAAGGGTTCTGCAAAGACGATAGAGGCAACCCTGCACTGTTAT~~~GGGGCCTACCTGACCATTGGGAAGAATCCTGATTTTAAGTCAACGTT
TpNIVA_d1288/62194580   389 TTGACACGAAGGGTTCTGCAAAGACGATAGAGGCAACCCTGCACTGTTAT~~~GGGGCCTACCTGACCATTGGGAAGAATCCTGATTTTAAGTCAACGTT
TpNIVB_d1274/56361402   389 TTGACACGAAGGGTTCTGCAAAGACGATAGAGGCAACCCTGCACTGTTAT~~~GGGGCCTACCTGACCATTGGGAAGAATCCTGATTTTAAGTCAACGTT
TpNIVB_d1274/57606550   389 TTGACACGAAGGGTTCTGCAAAGACGATAGAGGCAACCCTGCACTGTTAT~~~GGGGCCTACCTGACCATTGGGAAGAATCCTGATTTTAAGTCAACGTT
TpNCL2/71434737         389 TTGACACGAAGGGTTCTGCAAAGACGATAGAGGCAACCCTGCACTGTTAT~~~GGGGCCTACCTGACCATTGGGAAGAATCCTGATTTTAAGTCAACGTT
TpNCL5/23396777         389 TTGACACGAAGGGTTCTGCAAAGACGATAGAGGCAACCCTGCACTGTTAT~~~GGGGCCTACCTGACCATTGGGAAGAATCCTGATTTTAAGTCAACGTT
TpNCL8/37093850         389 TTGACACGAAGGGTTCTGCAAAGACGATAGAGGCAACCCTGCACTGTTAT~~~GGGGCCTACCTGACCATTGGGAAGAATCCTGATTTTAAGTCAACGTT
TpNIVB_d1274/39452938   389 TTGACACGAAGGGTTCTGCAAAGACGATAGAGGCAACCCTGCACTGTTAT~~~GGGGCCTACCTGACCATTGGGAAGAATCCTGATTTTAAGTCAACGTT
TpNIVA_d1288/9765540    389 TTGACACGAAGGGTTCTGCAAAGACGATAGAGGCAACCCTGCACTGTTAT~~~GGGGCCTACCTGACCATTGGGAAGAATCCTGATTTTAAGTCAACGTT
TpNCL3/13042505         398 TTGACACGAAGGGTTCTGCAAAGACGATAGAGGCAACCCTGCACTGTTAT~~~GGGGCCTACCTGACCATTGGGAAGAATCCTGATTTTAAGTCAACGTT
TpNCL3/50332092         398 TTGACACGAAGGGTTCTGCAAAGACGATAGAGGCAACCCTGCACTGTTTA~~~GGGGCCTACCTGACCATTGGGAAGAATCCTGATTTTAAGTCAACGTT
TpNCL3/24642061         398 TTGACACGAAGGGTTCTGCAAAGACGATAGAGGCAACCCTGCACTGTTATGGGGGGGCCTACCTGACCATTGGGAAGAATCCTGATTTTAAGTCAACGTT
TpNCL3/25952592         398 TTGACACGAAGGGTTCTGCAAAGACGATAGAGGCAACCCTGCACTGTTAT~~~GGGGCCTACCTGACCATTGGGAAGAATCCTGATTTTAAGTCAACGTT
TpNCL3/61080088         398 TTGACACGAAGGGTTCTGCAAAGACGATAGAGGCAACCCTGCACTGTTAT~~~GGGGCCTACCTGACCATTGGGAAGAATCCTGATTTTAAGTCAACGTT
TpNCL3/39322209         398 TTGACACGAAGGGTTCTGCAAAGACGATAGAGGCAACCCTGCACTGTTAT~~~GGGGCCTACCTGACCATTGGGAAGAATCCTGATTTTAAGTCAACGTT
TpNCL3/73007774         398 TTGACACGAAGGGTTCTGCAAAGACGATAGAGGCAACCCTGCACTGTTAT~~~GGGGCCTACCTGACCATTGGGAAGAATCCTGATTTTAAGTCAACGTT
TpNCL3/46924011         398 TTGACACGAAGGGTTCTGCAAAGACGATAGAGGCAACCCTGCACTGTTAT~~~GGGGCCTACCTGACCATTGGGAAGAATCCTGATTTTAAGTCAACGTT
TpNCL3/34013629         398 TTGACACGAAGGGTTCTGCAAAGACGATAGAGGCAACCCTGCACTGTTAT~~~GGGGCCTACCTGACCATTGGGAAGAATCCTGATTTTAAGTCAACGTT
TpNCL4/16253724         398 TTGACACGAAGGGTTCTGCAAAGACGATAGAGGCAACCCTGCACTGTTAT~~~GGGGCCTACCTGACCATTGGGAAGAATCCTGATTTTAAGTCAACGTT
TpNCL4/67830320         398 TTGACACGAAGGGTTCTGCAAAGACGATAGAGGCAACCCTGCACTGTTAT~~~GGGGCCTACCTGACCATTGGGAAGAATCCTGATTTTAAGTCAACGTT
TpNCL4/49087362         398 TTGACACGAAGGGTTCTGCAAAGACGATAGAGGCAACCCTGCACTGTTAT~~~GGGGCCTACCTGACCATTGGGAAGAATCCTGATTTTAAGTCAACGTT
TpNCL4/56558172         398 TTGACACGAAGGGTTCTGCAAAGACGATAGAGGCAACCCTGCACTGTTAT~~~GGGGCCTACCTGACCATTGGGAAGAATCCTGATTTTAAGTCAACGTT
TpNCL4/24117649         398 TTGACACGAAGGGTTCTGCAAAGACGATAGAGGCAACCCTGCACTGTTAT~~~GGGGCCTACCTGACCATTGGGAAGAATCCTGATTTTAAGTCAACGTT
TpNCL4/74580654         398 TTGACACGAAGGGTTCTGCAAAGACGATAGAGGCAACCCTGCACTGTTAT~~~GGGGCCTACCTGACCATTGGGAAGAATCCTGATTTTAAGTCAACGTT
TpNCL4/29164148         398 TTGACACGAAGGGTTCTGCAAAGACGATAGAGGCAACCCTGCACTGTTAT~~~GGGGCCTACCTGACCATTGGGAAGAATCCTGATTTTAAGTCAACGTT
TpNIVA_d1288/44237532   398 TTGACACGAAGGGTTCTGCAAAGACGATAGAGGCAACCCTGCACTGTTAT~~~GGGGCCTACCTGACCATTGGGAAGAATCCTGATTTTAAGTCAACGTT
TpNIVA_d1288/52757423   398 TTGACACGAAGGGTTCTGCAAAGACGATAGAGGCAACCCTGCACTGTTAT~~~GGGGCCTACCTGACCATTGGGAAGAATCCTGATTTTAAGTCAACGTT
TpNRabbit/68420554      401 TTGACACGAAGGGTTCTGCAAAGACGATAGAGGCAACCCTGCACTGTTAT~~~GGGGCCTACCTGACCATTGGGAAGAATCCTGATTTTAAGTCAACGTT
TpNIVA_d1288/72286947   398 TTGACACGAAGGGTTCTGCAAAGACGATAGAGGCAACCCTGCACTGTTAT~~~GGGGCCTACCTGACCATTGGGAAGAATCCTGATTTTAAGTCAACGTT
TpNIVA_d1288/15598261   389 TTGACACGAAGGGTTCTGCAAAGACGATAGAGGCAACCCTGCACTGTTAT~~~GGGGCCTACCTGACCATTGGGAAGAATCCTGATTTTAAGTCAACGTT
```

```
NC_021490.2_tprK       495 TGCTGTTTTGTGGGAGCCGTGGACCGCGAATGGGGATTATAAGTCTAAGGGAGATAAGCCGGTGTATGAGCCGGGGTTTGAGGGAGCCGGGGGAAAGTTA
TpNRabbit/11797378     492 TGCTGTTTTGTGGGAGCCGTGGACCGCGAATGGGGATTATAAGTCTAAGGGAGATAAGCCGGTGTATGAGCCGGGGTTTGAGGGAGCCGGGGGAAAGTTA
TpNRabbit/49086983     495 TGCTGTTTTGTGGGAGCCGTGGACCGCGAATGGGGATTATAAGTCTAAGGGAGATAAGCCGGTGTATGAGCCGGGGTTTGAGGGAGCCGGGGGAAAGTTA
TpNCL1/74908282        495 TGCTGTTTTGTGGGAGCCGTGGACCGCGAATGGGGATTATAAGTCTAAGGGAGATAAGCCGGTGTATGAGCCGGGGTTTGAGGGAGCCGGGGGAAAGTTA
TpNRabbit/74646114     495 TGCTGTTTTGTGGGAGCCGTGGACCGCGAATGGGGATTATAAGTCTAAGGGAGATAAGCCGGTGTATGAGCCGGGGTTTGAGGGAGCCGGGGGAAAGTTA
TpNIVA_d1288/58000085  495 TGCTGTTTTGTGGGAGCCGTGGACCGCGAATGGGGATTATAAGTCTAAGGGAGATAAGCCGGTGTATGAGCCGGGGTTTGAGGGAGCCGGGGGAAAGTTA
TpNCL1/67830753        495 TGCTGTTTTGTGGGAGCCGTGGACCGCGAATGGGGATTATAAGTCTAAGGGAGATAAGCCGGTGTATGAGCCGGGGTTTGAGGGAGCCGGGGGAAAGTTA
TpNRabbit/37356380     486 TGCTGTTTTGTGGGAGCCGTGGACCGCGAATGGGGATTATAAGTCTAAGGGAGATAAGCCGGTGTATGAGCCGGGGTTTGAGGGAGCCGGGGGAAAGTTA
TpNIVA_d1288/62194580  486 TGCTGTTTTGTGGGAGCCGTGGACCGCGAATGGGGATTATAAGTCTAAGGGAGATAAGCCGGTGTATGAGCCGGGGTTTGAGGGAGCCGGGGGAAAGTTA
TpNIVB_d1274/56361402  486 TGCTGTTTTGTGGGAGCCGTGGACCGCGAATGGGGATTATAAGTCTAAGGGAGATAAGCCGGTGTATGAGCCGGGGTTTGAGGGAGCCGGGGGAAAGTTA
TpNIVB_d1274/57606550  486 TGCTGTTTTGTGGGAGCCGTGGACCGCGAATGGGGATTATAAGTCTAAGGGAGATAAGCCGGTGTATGAGCCGGGGTTTGAGGGAGCCGGGGGAAAGTTA
TpNCL2/71434737        486 TGCTGTTTTGTGGGAGCCGTGGACCGCGAATGGGGATTATAAGTCTAAGGGAGATAAGCCGGTGTATGAGCCGGGGTTTGAGGGAGCCGGGGGAAAGTTA
TpNCL5/23396777        486 TGCTGTTTTGTGGGAGCCGTGGACCGCGAATGGGGATTATAAGTCTAAGGGAGATAAGCCGGTGTATGAGCCGGGGTTTGAGGGAGCCGGGGGAAAGTTA
TpNCL8/37093850        486 TGCTGTTTTGTGGGAGCCGTGGACCGCGAATGGGGATTATAAGTCTAAGGGAGATAAGCCGGTGTATGAGCCGGGGTTTGAGGGAGCCGGGGGAAAGTTA
TpNIVB_d1274/39452938  486 TGCTGTTTTGTGGGAGCCGTGGACCGCGAATGGGGATTATAAGTCTAAGGGAGATAAGCCGGTGTATGAGCCGGGGTTTGAGGGAGCCGGGGGAAAGTTA
TpNIVA_d1288/9765540   486 GGCTGTTTTGTGGGAGCCGTGGACCGCGAATGGGGATTATAAGTCTAAGGGAGATAAGCCGGTGTATGAGCCGGGGTTTGAGGGAGCCGGGGGAAAGTTA
TpNCL3/13042505        495 TGCTGTTTTGTGGGAGCCGTGGACCGCGAATGGGGATTATAAGTCTAAGGGAGATAAGCCGGTGTATGAGCCGGGGTTTGAGGGAGCCGGGGGAAAGTTA
TpNCL3/50332092        495 TGCTGTTTTGTGGGAGCCGTGGACCGCGAATGGGGATTATAAGTCTAAGGGAGATAAGCCGGTGTATGAGCCGGGGTTTGAGGGAGCCGGGGGAAAGTTA
TpNCL3/24642061        498 TGCTGTTTTGTGGGAGCCGTGGACCGCGAATGGGGATTATAAGTCTAAGGGAGATAAGCCGGTGTATGAGCCGGGGTTTGAGGGAGCCGGGGGAAAGTTA
TpNCL3/25952592        495 TGCTGTTTTGTGGGAGCCGTGGACCGCGAATGGGGATTATAAGTCTAAGGGAGATAAGCCGGTGTATGAGCCGGGGTTTGAGGGAGCCGGGGGAAAGTTA
TpNCL3/61080088        495 TGCTGTTTTGTGGGAGCCGTGGACCGCGAATGGGGATTATAAGTCTAAGGGAGATAAGCCGGTGTATGAGCCGGGGTTTGAGGGAGCCGGGGGAAAGTTA
TpNCL3/39322209        495 TGCTGTTTTGTGGGAGCCGTGGACCGCGAATGGGGATTATAAGTCTAAGGGAGATAAGCCGGTGTATGAGCCGGGGTTTGAGGGAGCCGGGGGAAAGTTA
TpNCL3/73007774        495 TGCTGTTTTGTGGGAGCCGTGGACCGCGAATGGGGATTATAAGTCTAAGGGAGATAAGCCGGTGTATGAGCCGGGGTTTGAGGGAGCCGGGGGAAAGTTA
TpNCL3/46924011        495 TGCTGTTTTGTGGGAGCCGTGGACCGCGAATGGGGATTATAAGTCTAAGGGAGATAAGCCGGTGTATGAGCCGGGGTTTGAGGGAGCCGGGGGAAAGTTA
TpNCL3/34013629        495 TGCTGTTTTGTGGGAGCCGTGGACCGCGAATGGGGATTATAAGTCTAAGGGAGATAAGCCGGTGTATGAGCCGGGGTTTGAGGGAGCCGGGGGAAAGTTA
TpNCL4/16253724        495 TGCTGTTTTGTGGGAGCCGTGGACCGCGAATGGGGATTATAAGTCTAAGGGAGATAAGCCGGTGTATGAGCCGGGGTTTGAGGGAGCCGGGGGAAAGTTA
TpNCL4/67830320        495 TGCTGTTTTGTGGGAGCCGTGGACCGCGAATGGGGATTATAAGTCTAAGGGAGATAAGCCGGTGTATGAGCCGGGGTTTGAGGGAGCCGGGGGAAAGTTA
TpNCL4/49087362        495 TGCTGTTTTGTGGGAGCCGTGGACCGCGAATGGGGATTATAAGTCTAAGGGAGATAAGCCGGTGTATGAGCCGGGGTTTGAGGGAGCCGGGGGAAAGTTA
TpNCL4/56558172        495 TGCTGTTTTGTGGGAGCCGTGGACCGCGAATGGGGATTATAAGTCTAAGGGAGATAAGCCGGTGTATGAGCCGGGGTTTGAGGGAGCCGGGGGAAAGTTA
TpNCL4/24117649        495 TGCTGTTTTGTGGGAGCCGTGGACCGCGAATGGGGATTATAAGTCTAAGGGAGATAAGCCGGTGTATGAGCCGGGGTTTGAGGGAGCCGGGGGAAAGTTA
TpNCL4/74580654        495 TGCTGTTTTGTGGGAGCCGTGGACCGCGAATGGGGATTATAAGTCTAAGGGAGATAAGCCGGTGTATGAGCCGGGGTTTGAGGGAGCCGGGGGAAAGTTA
TpNCL4/29164148        495 TGCTGTTTTGTGGGAGCCGTGGACCGCGAATGGGGATTATAAGTCTAAGGGAGATAAGCCGGTGTATGAGCCGGGGTTTGAGGGAGCCGGGGGAAAGTTA
TpNIVA_d1288/44237532  495 TGCTGTTTTGTGGGAGCCGTGGACCGCGAATGGGGATTATAAGTCTAAGGGAGATAAGCCGGTGTATGAGCCGGGGTTTGAGGGAGCCGTGGGAAAGTTA
TpNIVA_d1288/52757423  495 TGCTGTTTTGTGGGAGCCGTGGACCGCGAATGGGGATTATAAGTCTAAGGGAGATAAGCCGGTGTATGAGCCGGGGTTTGAGGGAGCCGGGGGAAAGTTA
TpNRabbit/68420554     498 TGCTGTTTTGTGGGAGCCGTGGACCGCGAATGGGGATTATAAGTCTAAGGGAGATAAGCCGGTGTATGAGCCGGGGTTTGAGGGAGCCGGGGGAAAGTTA
TpNIVA_d1288/72286947  495 TGCTGTTTTGTGGGAGCCGTGGACCGCGAATGGGGATTATAAGTCTAAGGGAGATAAGCCGGTGTATGAGCCGGGGTTTGAGGGAGCCGGGGGAAAGTTA
TpNIVA_d1288/15598261  486 TGCTGTTTTGTGGGAGCCGTGGACCGCGAATGGGGATTATAAGTCTAAGGGAGATAAGCCGGTGTATGAGCCGGGGTTTGAGGGAGCCGGGGGAAAGTTA
```

VR2

```
NC_021490.2_tprK          595 GGGTATAAACAGACTGACATCGCCGGCACGGGGCTCACGTTTGATATTGCGTTTAAGTTTGCGTCTAACACCGACTGGGAGGGCAAAGACAGCAAGGGCA
TpNRabbit/11797378        592 GGGTATAAACAGACTGACATCGCCGGCACGGGGCTCACGTTTGATATTGCGTTTAAGTTTGCGTCTAACACCGACTGGGAGGGCAAAGACAGCAAGGGCA
TpNRabbit/49086983        595 GGGTATAAACAGACTGACATCGCCGGCACGGGGCTCACGTTTGATATTGCGTTTAAGTTTGCGTCTAACACCGACTGGGAGGGCAAAGACAGCAAGGGCA
TpNCL1/74908282           595 GGGTATAAACAGACTGACATCGCCGGCACGGGGCTCACGTTTGATATTGCGTTTAAGTTTGCGTCTAACACCGACTGGGAGGGCAAAGACAGCAAGGGCA
TpNRabbit/74646114        595 GGGTATAAACAGACTGACATCGCCGGCACGGGGCTCACGTTTGATATTGCGTTTAAGTTTGCGTCTAACACCGACTGGGAGGGCAAAGACAGCAAGGGCA
TpNIVA_d1288/58000085     595 GGGTATAAACAGACTGACATCGCCGGCACGGGGCTCACGTTTGATATTGCGTTTAAGTTTGCGTCTAACACCGACTGGGAGGGCAAAGACAGCAAGGGCA
TpNCL1/67830753           595 GGGTATAAACAGACTGACATCGCCGGCACGGGGCTCACGTTTGATATTGCGTTTAAGTTTGCGTCTAACACCGACTGGGAGGGCAAAGACAGCAAGGGCA
TpNRabbit/37356380        586 GGGTATAAACAGACTGACATCGCCGGCACGGGGCTCACGTTTGATATTGCGTTTAAGTTTGCGTCTAACACCGACTGGGAGGGCAAAGACAGCAAGGGCA
TpNIVA_d1288/62194580     586 GGGTATAAACAGACTGACATCGCCGGCACGGGGCTCACGTTTGATATTGCGTTTAAGTTTGCGTCTAACACCGACTGGGAGGGCAAAGACAGCAAGGGCA
TpNIVB_d1274/56361402     586 GGGTATAAACAGACTGACATCGCCGGCACGGGGCTCACGTTTGATATTGCGTTTAAGTTTGCGTCTAACACCGACTGGGAGGGCAAAGACAGCAAGGGCA
TpNIVB_d1274/57606550     586 GGGTATAAACAGACTGACATCGCCGGCACGGGGCTCACGTTTGATATTGCGTTTAAGTTTGCGTCTAACACCGACTGGGAGGGCAAAGACAGCAAGGGCA
TpNCL2/71434737           586 GGGTATAAACAGACTGACATCGCCGGCACGGGGCTCACGTTTGATATTGCGTTTAAGTTTGCGTCTAACACCGACTGGGAGGGCAAAGACAGCAAGGGCA
TpNCL5/23396777           586 GGGTATAAACAGACTGACATCGCCGGCACGGGGCTCACGTTTGATATTGCGTTTAAGTTTGCGTCTAACACCGACTGGGAGGGCAAAGACAGCAAGGGCA
TpNCL8/37093850           586 GGGTATAAACAGACTGACATCGCCGGCACGGGGCTCACGTTTGATATTGCGTTTAAGTTTGCGTCTAACACCGACTGGGAGGGCAAAGACAGCAAGGGCA
TpNIVB_d1274/39452938     586 GGGTATAAACAGACTGACATCGCCGGCACGGGGCTCACGTTTGATATTGCGTTTAAGTTTGCGTCTAACACCGACTGGGAGGGCAAAGACAGCAAGGGCA
TpNIVA_d1288/9765540      586 GGGTATAAACAGACTGACATCGCCGGCACGGGGCTCACGTTTGATATTGCGTTTAAGTTTGCGTCTAACACCGACTGGGAGGGCAAAGACAGCAAGGGCA
TpNCL3/13042505           595 GGGTATAAACAGACTGACATCGCCGGCACGGGGCTCACGTTTGATATTGCGTTTAAGTTTGCGTCTAACACCGACTGGGAGGGCAAAGACAGCAAGGGCA
TpNCL3/50332092           595 GGGTATAAACAGACTGACATCGCCGGCACGGGGCTCACGTTTGATATTGCGTTTAAGTTTGCGTCTAACACCGACTGGGAGGGCAAAGACAGCAAGGGCA
TpNCL3/24642061           598 GGGTATAAACAGACTGACATCGCCGGCACGGGGCTCACGTTTGATATTGCGTTTAAGTTTGCGTCTAACACCGACTGGGAGGGCAAAGACAGCAAGGGCA
TpNCL3/25952592           595 GGGTATAAACAGACTGACATCGCCGGCACGGGGCTCACGTTTGATATTGCGTTTAAGTTTGCGTCTAACACCGACTGGGAGGGCAAAGACAGCAAGGGCA
TpNCL3/61080088           595 GGGTATAAACAGACTGACATCGCCGGCACGGGGCTCACGTTTGATATTGCGTTTAAGTTTGCGTCTAACACCGACTGGGAGGGCAAAGACAGCAAGGGCA
TpNCL3/39322209           595 GGGTATAAACAGACTGACATCGCCGGCACGGGGCTCACGTTTGATATTGCGTTTAAGTTTGCGTCTAACACCGACTGGGAGGGCAAGTCAAACACCGGGCG
TpNCL3/73007774           595 GGGTATAAACAGACTGACATCGCCGGCACGGGGCTCACGTTTGATATTGCGTTTAAGTTTGCGTCTAACACCGACTGGGAGGGCAAAGACAGCAAGGGCA
TpNCL3/46924011           595 GGGTATAAACAGACTGACATCGCCGGCACGGGGCTCACGTTTGATATTGCGTTTAAGTTTGCGTCTAACACCGACTGGGAGGGCAAAGACAGCAAGGGCA
TpNCL3/34013629           595 GGGTATAAACAGACTGACATCGCCGGCACGGGGCTCACGTTTGATATTGCGTTTAAGTTTGCGTCTAACACCGACTGGGAGGGCAAACCAAAC~~~GGCA
TpNCL4/16253724           595 GGGTATAAACAGACTGACATCGCCGGCACGGGGCTCACGTTTGATATTGCGTTTAAGTTTGCGTCTAACACCGACTGGGAGGGCAAAGACAGCAAGGGCA
TpNCL4/67830320           595 GGGTATAAACAGACTGACATCGCCGGCACGGGGCTCACGTTTGATATTGCGTTTAAGTTTGCGTCTAACACCGACTGGGAGGGCAAAGACAGCAAGGGCA
TpNCL4/49087362           595 GGGTATAAACAGACTGACATCGCCGGCACGGGGCTCACGTTTGATATTGCGTTTAAGTTTGCGTCTAACACCGACTGGGAGGGCAAAGACAGCAAGGGCA
TpNCL4/56558172           595 GGGTATAAACAGACTGACATCGCCGGCACGGGGCTCACGTTTGATATTGCGTTTAAGTTTGCGTCTAACACCGACTGGGAGGGCAAAGACAGCAAGGGCA
TpNCL4/24117649           595 GGGTATAAACAGACTGACATCGCCGGCACGGGGCTCACGTTTGATATTGCGTTTAAGTTTGCGTCTAACACCGACTGGGAGGGCAAAGACAGCAAGGGCA
TpNCL4/74580654           595 GGGTATAAACAGACTGACATCGCCGGCACGGGGCTCACGTTTGATATTGCGTTTAAGTTTGCGTCTAACACCGACTGGGAGGGCAAAGACAGCAAGGGCA
TpNCL4/29164148           595 GGGTATAAACAGACTGACATCGCCGGCACGGGGCTCACGTTTGATATTGCGTTTAAGTTTGCGTCTAACACCGACTGGGAGGGCAAAGACAGCAAGGGCG
TpNIVA_d1288/44237532     595 GGGTATAAACAGACTGACATCGCCGGCACGGGGCTCACGTTTGATATTGCGTTTAAGTTTGCGTCTAACACCGACTGGGAGGGCAAAGACAGTCAGGGCA
TpNIVA_d1288/52757423     595 GGGTATAAACAGACTGACATCGCCGGCACGGGGCTCACGTTTGATATTGCGTTTAAGTTTGCGTCTAACACCGACTGGGAGGGCAAAGACAGTCAGGGCA
TpNRabbit/68420554        598 GGGTATAAACAGACTGACATCGCCGGCACGGGGCTCACGTTTGATATTGCGTTTAAGTTTGCGTCTAACACCGACTGGGAGGGCAAAGACAGCAAGGGCA
TpNIVA_d1288/72286947     595 GGGTATAAACAGACTGACATCGCCGGCACGGGGCTCACGTTTGATATTGCGTTTAAGTTTGCGTCTAACACCGACTGGGAGGGCAAAGACAGCAAGGGCG
TpNIVA_d1288/15598261     586 GGGTATAAACAGACTGACATCGCCGGCACGGGGCTCACGTTTGATATTGCGTTTAAGTTTGCGTCTAACACCGACTGGGAGGGCAAAGACAGCAAGGGCA
```

```
                                  VR2
NC_021490.2_tprK          695 ACGTCCCAGCAGGAGTAACCCCCAGCAAGTATGGATTGGGGGGAGATATTTTGTTCGGCTGGGAGCGTACGCGTGAAGATGGCGTGCAGGAATACATTAA
TpNRabbit/11797378        692 ACGTCCCAGCAGGAGTAACCCCCAGCAAGTATGGATTGGGGGGAGATATTTTGTTCGGCTGGGAGCGTACGCGTGAAGATGGCGTGCAGGAATACATTAA
TpNRabbit/49086983        695 ACGTCCCAGCAGGAGTAACCCCCAGCAAGTATGGATTGGGGGGAGATATTTTGTTCGGCTGGGAGCGTACGCGTGAAGATGGCGTGCAGGAATACATTAA
TpNCL1/74908282           695 ACGTCCCAGCAGGAGTAACCCCCAGCAAGTATGGATTGGGGGGAGATATTTTGTTCGGCTGGGAGCGTACGCGTGAAGATGGCGTGCAGGAATACATTAA
TpNRabbit/74646114        695 ACGTCCCAGCAGGAGTAACCCCCAGCAAGTATGGATTGGGGGGAGATATTTTGTTCGGCTGGGAGCGTACGCGTGAAGATGGCGTGCAGGAATACATTAA
TpNIVA_d1288/58000085     695 ACGTCCCAGCAGGAGTAACCCCCAGCAAGTATGGATTGGGGGGAGATATTTTGTTCGGCTGGGAGCGTACGCGTGAAGATGGCGTGCAGGAATACATTAA
TpNCL1/67830753           695 ACGTCCCAGCAGGAGTAACCCCCAGCAAGTATGGATTGGGGGGAGATATTTTGTTCGGCTGGGAGCGTACGCGTGAAGATGGCGTGCAGGAATACATTAA
TpNRabbit/37356380        686 ACGTCCCAGCAGGAGTAACCCCCAGCAAGTATGGATTGGGGGGAGATATTTTGTTCGGCTGGGAGCGTACGCGTGAAGATGGCGTGCAGGAATACATTAA
TpNIVA_d1288/62194580     686 ACGTCCCAGCAGGAGTAACCCCCAGCAAGTATGGATTGGGGGGAGATATTTTGTTCGGCTGGGAGCGTACGCGTGAAGATGGCGTGCAGGAATACATTAA
TpNIVB_d1274/56361402     686 ACGTCCCAGCAGGAGTAACCCCCAGCAAGTATGGATTGGGGGGAGATATTTTGTTCGGCTGGGAGCGTACGCGTGAAGATGGCGTGCAGGAATACATTAA
TpNIVB_d1274/57606550     686 ACGTCCCAGCAGGAGTAACCCCCAGCAAGTATGGATTGGGGGGAGATATTTTGTTCGGCTGGGAGCGTACGCGTGAAGATGGCGTGCAGGAATACATTAA
TpNCL2/71434737           686 ACGTCCCAGCAGGAGTAACCCCCAGCAAGTATGGATTGGGGGGAGATATTTTGTTCGGCTGGGAGCGTACGCGTGAAGATGGCGTGCAGGAATACATTAA
TpNCL5/23396777           686 ACGTCCCAGCAGGAGTAACCCCCAGCAAGTATGGATTGGGGGGAGATATTTTGTTCGGCTGGGAGCGTACGCGTGAAGATGGCGTGCAGGAATACATTAA
TpNCL8/37093850           686 ACGTCCCAGCAGGAGTAACCCCCAGCAAGTATGGATTGGGGGGAGATATTTTGTTCGGCTGGGAGCGTACGCGTGAAGATGGCGTGCAGGAATACATTAA
TpNIVB_d1274/39452938     686 ACGTCCCAGCAGGAGTAACCCCCAGCAAGTATGGATTGGGGGGAGATATTTTGTTCGGCTGGGAGCGTACGCGTGAAGATGGCGTGCAGGAATACATTAA
TpNIVA_d1288/9765540      686 ACGTCCCAGCAGGAGTAACCCCCAGCAAGTATGGATTGGGGGGAGATATTTTGTTCGGCTGGGAGCGTACGCGTGAAGATGGCGTGCAGGAATACATTAA
TpNCL3/13042505           695 ACGTCCCAGCAGGAGTAACCCCCAGCAAGTATGGATTGGGGGGAGATATTTTGTTCGGCTGGGAGCGTACGCGTGAAGATGGCGTGCAGGAATACATTAA
TpNCL3/50332092           695 ACGTCCCAGCAGGAGTAACCCCCAGCAAGTATGGATTGGGGGGAGATATTTTGTTCGGCTGGGAGCGTACGCGTGAAGATGGCGTGCAGGAATACATTAA
TpNCL3/24642061           698 ACGTCCCAGCAGGAGTAACCCCCAGCAAGTATGGATTGGGGGGAGATATTTTGTTCGGCTGGGAGCGTACGCGTGAAGATGGCGTGCAGGAATACATTAA
TpNCL3/25952592           695 ACGTCCCAGCAGGAGTAACCCCCAGCAAGTATGGATTGGGGGGAGATATTTTGTTCGGCTGGGAGCGTACGCGTGAAGATGGCGTGCAGGAATACATTAA
TpNCL3/61080088           695 AGGCCCCAGCAGGA~~~ACTCCCAGCAAGTATGGATTGGGGGGAGATATTTTGTTCGGCTGGGAGCGTACGCGTGAAGATGGCGTGCAGGAATACATTAA
TpNCL3/39322209           695 CCGCCCGAGCAGGAGTAACCCCCAGCAAGTATGGATTGGGGGGAGATATTTTGTTCGGCTGGGAGCGTACGCGTGAAGATGGCGTGCAGGAATACATTAA
TpNCL3/73007774           695 ACGTCCCAGCAGGAGTAACCCCCAGCAAGTATGGATTGGGGGGAGATATTTTGTTCGGCTGGGAGCGTACGCGTGAAGATGGCGTGCAGGAATACATTAA
TpNCL3/46924011           695 ACGTCCCAGCAGGAGTAACCCCCAGCAAGTATGGATTGGGGGGAGATATTTTGTTCGGCTGGGAGCGTACGCGTGAAGATGGCGTGCAGGAATACATTAA
TpNCL3/34013629           692 ACGTCCCAGCAGGAGTAACCCCCAGCAAGTATGGATTGGGGGGAGATATTTTGTTCGGCTGGGAGCGTACGCGTGAAGATGGCGTGCAGGAATACATTAA
TpNCL4/16253724           695 ACGTCCCAGCAGGAGTAACCCCCAGCAAGTATGGATTGGGGGGAGATATTTTGTTCGGCTGGGAGCGTACGCGTGAAGATGGCGTGCAGGAATACATTAA
TpNCL4/67830320           695 ACGTCCCAGCAGGAGTAACCCCCAGCAAGTATGGATTGGGGGGAGATATTTTGTTCGGCTGGGAGCGTACGCGTGAAGATGGCGTGCAGGAATACATTAA
TpNCL4/49087362           695 ACGTCCCAGCAGGAGTAACCCCCAGCAAGTATGGATTGGGGGGAGATATTTTGTTCGGCTGGGAGCGTACGCGTGAAGATGGCGTGCAGGAATACATTAA
TpNCL4/56558172           695 ACGTCCCAGCAGGAGTAACCCCCAGCAAGTATGGATTGGGGGGAGATATTTTGTTCGGCTGGGAGCGTACGCGTGAAGATGGCGTGCAGGAATACATTAA
TpNCL4/24117649           695 ACGTCCCAGCAGGAGTAACCCCCAGCAAGTATGGATTGGGGGGAGATATTTTGTTCGGCTGGGAGCGTACGCGTGAAGATGGCGTGCAGGAATACATTAA
TpNCL4/74580654           695 ACGTCCCAGCAGGAGTAACCCCCAGCAAGTATGGATTGGGGGGAGATATTTTGTTCGGCTGGGAGCGTACGCGTGAAGATGGCGTGCAGGAATACATTAA
TpNCL4/29164148           695 TCGTCCAAGCAGGAGTAACCCCCAGCAAGTATGGATTGGGGGGAGATATTTTGTTCGGCTGGGAGCGTACGCGTGAAGATGGCGTGCAGGAATACATTAA
TpNIVA_d1288/44237532     695 AGGCCCCAGCAGGAGTAACCCCCAGCAAGTATGGATTGGGGGGAGATATTTTGTTCGGCTGGGAGCGTACGCGTGAAGATGGCGTGCAGGAATACATTAA
TpNIVA_d1288/52757423     695 AGGCCCCAGCAGGAGTAACCCCCAGCAAGTATGGATTGGGGGGAGATATTTTGTTCGGCTGGGAGCGTACGCGTGAAGATGGCGTGCAGGAATACATTAA
TpNRabbit/68420554        698 ACGTCCACGCAGGAGTAACCCCCAGCAAGTATGGATTGGGGGGAGATATTTTGTTCGGCTGGGAGCGTACGCGTGAAGATGGCGTGCAGGAATACATTAA
TpNIVA_d1288/72286947     695 TCGTCCAAGCAGGAGTAACCCCCAGCAAGTATGGATTGGGGGGAGATATTTTGTTCGGCTGGGAGCGTACGCGTGAAGATGGCGTGCAGGAATACATTAA
TpNIVA_d1288/15598261     686 ACGTCCCAGCAGGAGTAACCCCCAGCAAGTATGGATTGGGGGGAGATATTTTGTTCGGCTGGGAGCGTACGCGTGAAGATGGCGTGCAGGAATACATTAA
```

```
                                                                         VR3
NC_021490.2_tprK           795 AGTGGAGCTCACCGGCAACTCCACACTGTCTAGCGACTAT~~~GCCCAAGCC~~~CGAGCCCTGGCAGCCGGGGCTAAGGTGAGTATGAAGCTTTGGGGT
TpNRabbit/11797378         792 AGTGGAGCTCACCGGCAACTCCACACTGTCTAGCGACTAT~~~GCCCAAGCC~~~CGAGCCCTGGCAGCCGGGGCTAAGGTGAGTATGAAGCTTTGGGGT
TpNRabbit/49086983         795 AGTGGAGCTCACCGGCAACTCCACACTGTCTAGCGACTAT~~~GCCCAAGCC~~~CGAGCCCTGGCAGCCGGGGCTAAGGTGAGTATGAAGCTTTGGGGT
TpNCL1/74908282            795 AGTGGAGCTCACCGGCAACTCCACACTGTCTAGCGACTAT~~~GCCCAAGCC~~~CGAGCCCTGGCAGCCGGGGCTAAGGTGAGTATGAAGCTTTGGGGT
TpNRabbit/74646114         795 AGTGGAGCTCACCGGCAACTCCACACTGTCTAGCGACTAT~~~GCCCAAGCC~~~CGAGCCCTGGCAGCCGGGGCTAAGGTGAGTATGAAGCTTTGGGGT
TpNIVA_d1288/58000085      795 AGTGGAGCTCACCGGCAACTCCACACTGTCTAGCGACTAT~~~GCCCAAGCC~~~CGAGCCCTGGCAGCCGGGGCTAAGGTGAGTATGAAGCTTTGGGGT
TpNCL1/67830753            795 AGTGGAGCTCACCGGCAACTCCACACTGTCTAGCGACTAT~~~GCCCAAGCC~~~CGAGCCCTGGCAGCCGGGGCTAAGGTGAGTATGAAGCTTTGGGGT
TpNRabbit/37356380         786 AGTGGAGCTCACCGGCAACTCCACACTGTCTAGCGACTAT~~~GCCCAAGCC~~~CGAGCCCTGGCAGCCGGGGCTAAGGTGAGTATGAAGCTTTGGGGT
TpNIVA_d1288/62194580      786 AGTGGAGCTCACCGGCAACTCCACACTGTCTAGCGACTAT~~~GCCCAAGCC~~~CGAGCCCTGGCAGCCGGGGCTAAGGTGAGTATGAAGCTTTGGGGT
TpNIVB_d1274/56361402      786 AGTGGAGCTCACCGGCAACTCCACACTGTCTAGCGACTAT~~~GCCCAAGCC~~~CGAGCCCTGGCAGCCGGGGCTAAGGTGAGTATGAAGCTTTGGGGT
TpNIVB_d1274/57606550      786 AGTGGAGCTCACCGGCAACTCCACACTGTCTAGCGACTAT~~~GCCCAAGCC~~~CGAGCCCTGGCAGCCGGGGCTAAGGTGAGTATGAAGCTTTGGGGT
TpNCL2/71434737            786 AGTGGAGCTCACCGGCAACTCCACACTGTCTAGCGACTAT~~~GCCCAAGCC~~~CGAGCCCTGGCAGCCGGGGCTAAGGTGAGTATGAAGCTTTGGGGT
TpNCL5/23396777            786 AGTGGAGCTCACCGGCAACTCCACACTGTCTAGCGACTAT~~~GCCCAAGCC~~~CGAGCCCTGGCAGCCGGGGCTAAGGTGAGTATGAAGCTTTGGGGT
TpNCL8/37093850            786 AGTGGAGCTCACCGGCAACTCCACACTGTCTAGCGACTAT~~~GCCCAAGCC~~~CGAGCCCTGGCAGCCGGGGCTAAGGTGAGTATGAAGCTTTGGGGT
TpNIVB_d1274/39452938      786 AGTGGAGCTCACCGGCAACTCCCACCTGTCTAGCGACTAT~~~GCCCAAGCC~~~CGAGCCCTGGCAGCCGGGGCTAAGGTGAGTATGAAGCTTTGGGGT
TpNIVA_d1288/9765540       786 AGTGGAGCTCACCGGCAACTCCACACTGTCTAGCGACTAT~~~GCCCAAGCC~~~CGAGCCCTGGCAGCCGGGGCTAAGGTGAGTATGAAGCTTTGGGGT
TpNCL3/13042505            795 AGTGGAGCTCACCGGCAACTCCACACTGTCTAGCGGCTAT~~~GCCCAAGCAGCCCGAGCCCTGGCAGCCGGGGCTAAGGTGAGTATGAAGCTTTGGGGT
TpNCL3/50332092            795 AGTGGAGCTCACCGGCAACTCCACACTGTCTAGCGGCTAT~~~GCCCAAGCAGCCCGAGCCCTGGCAGCCGGGGCTAAGGTGAGTATGAAGCTTTGGGGT
TpNCL3/24642061            798 AGTGGAGCTCACCGGCAACTCCACACTGTCTAGCGGCTAT~~~GCCCAAGCAGCCCGAGCCCTGGCAGCCGGGGCTAAGGTGAGTATGAAGCTTTGGGGT
TpNCL3/25952592            795 AGTGGAGCTCACCGGCAACTCCACACTGTCTAGCGGCTAT~~~GCCCAAGCAGCCCCAGCC~~~GCAGCCGGGGCTAAGGTGAGTATGAAGCTTTGGGGT
TpNCL3/61080088            792 AGTGGAGCTCACCGGCAACTCCACACTGTCTAGCGGCTAT~~~GCCCAAGCAGCCCGAGCCCTGGCAGCCGGGGCTAAGGTGAGTATGAAGCTTTGGGGT
TpNCL3/39322209            795 AGTGGAGCTCACCGGCAACTCCACACTGTCTAGCGGCTAT~~~GCCCAAGCAGCCCGAGCCCTGGCAGCCGGGGCTAAGGTGAGTATGAAGCTTTGGGGT
TpNCL3/73007774            795 AGTGGAGCTCACCGGCAACTCCACACTGTCTAGCGGCTAT~~~GCCCAAGCAGCCCGAGCCCTGGCAGCCGGGGCTAAGGTGAGTATGAAGCTTTGGGGT
TpNCL3/46924011            795 AGTGGAGCTCACCGGCAACTCCACACTGTCTAGCGGCTAT~~~GCCCAAGCAGCCCGAGCCCTGGCAGCCGGGGCTAAGGTGAGTATGAAGCTTTGGGGT
TpNCL3/34013629            792 AGTGGAGCTCACCGGCAACTCCACACTGTCTAGCGGCTAT~~~GCCCAAGCAGCCCGAGCCCTGGCAGCCGGGGCTAAGGTGAGTATGAAGCTTTGGGGT
TpNCL4/16253724            795 AGTGGAGCTCACCGGCAACTCCACACTGTCTAGCGGCTAT~~~GCCCAAGCAGCCCGAGCCCTGGCAGCCGGGGCTAAGGTGAGTATGAAGCTTTGGGGT
TpNCL4/67830320            795 AGTGGAGCTCACCGGCAACTCCACACTGTCTAGCGGCTAT~~~GCCCAAGCAGCCCGAGCCCTGGCAGCCGGGGCTAAGGTGAGTATGAAGCTTTGGGGT
TpNCL4/49087362            795 AGTGGAGCTCACCGGCAACTCCACACTGTCTAGCGGCTAT~~~GCCCAAGCAGCCCGAGCCCTGGCAGCCGGGGCTAAGGTGAGTATGAAGCTTTGGGGT
TpNCL4/56558172            795 AGTGGAGCTCACCGGCAACTCCACACTGTCTAGCGGCTAT~~~GCCCAAGCAGCCCGAGCCCTGGCAGCCGGGGCTAAGGTGAGTATGAAGCTTTGGGGT
TpNCL4/24117649            795 AGTGGAGCTCACCGGCAACTCCACACTGTCTAGCGGCTAT~~~GCCCAAGCAGCCCGAGCCCTGGCAGCCGGGGCTAAGGTGAGTATGAAGCTTTGGGGT
TpNCL4/74580654            795 AGTGGAGCTCACCGGCAACTCCACACTGTCTAGCGGCTAT~~~GCCCAAGCAGCCCGAGCCCTGGCAGCCGGGGCTAAGGTGAGTATGAAGCTTTGGGGT
TpNCL4/29164148            795 AGTGGAGCTCACCGGCAACTCCACACTGTCTAGCGGCTAT~~~GCCCAAGCAGCCCGAGCCCTGGCAGCCGGGGCTAAGGTGAGTATGAAGCTTTGGGGT
TpNIVA_d1288/44237532      795 AGTGGAGCTCACCGGCAACTCCACACTGTCTAGCGGCTAT~~~GCCCAAGCAGCCCGAGCCCTGGCAGCCGGGGCTAAGGTGAGTATGAAGCTTTGGGGT
TpNIVA_d1288/52757423      795 AGTGGAGCTCACCGGCAACTCCACACTGTCTAGCGGCTAT~~~GCCCAAGCAGCCCGAGCCCTGGCAGCCGGGGCTAAGGTGAGTATGAAGCTTTGGGGT
TpNRabbit/68420554         798 AGTGGAGCTCACCGGCAACTCCACACTGTCTAGCGGCTATNATGCCCAAGCAGCCCGAGCCCTGGCAGCCGGGGCTAAGGTGAGTATGAAGCTTTGGGGT
TpNIVA_d1288/72286947      795 AGTGGAGCTCACCGGCAACTCCACACTGTCTAGCGACTAT~~~~~~GCCCAAGCCCGAGCCCTGGCAGCCGGGGCTAAGGTGAGTATGAAGCTTTGGGGT
TpNIVA_d1288/15598261      786 AGTGGAGCTCACCGGCAACTCCACACTGTCTAGCGGCTAT~~~~~~GCCCAAGCAGCCGGAGCCGCAGCCGGGGCTAAGGTGAGTATGAAGCTTTGGGGT
```

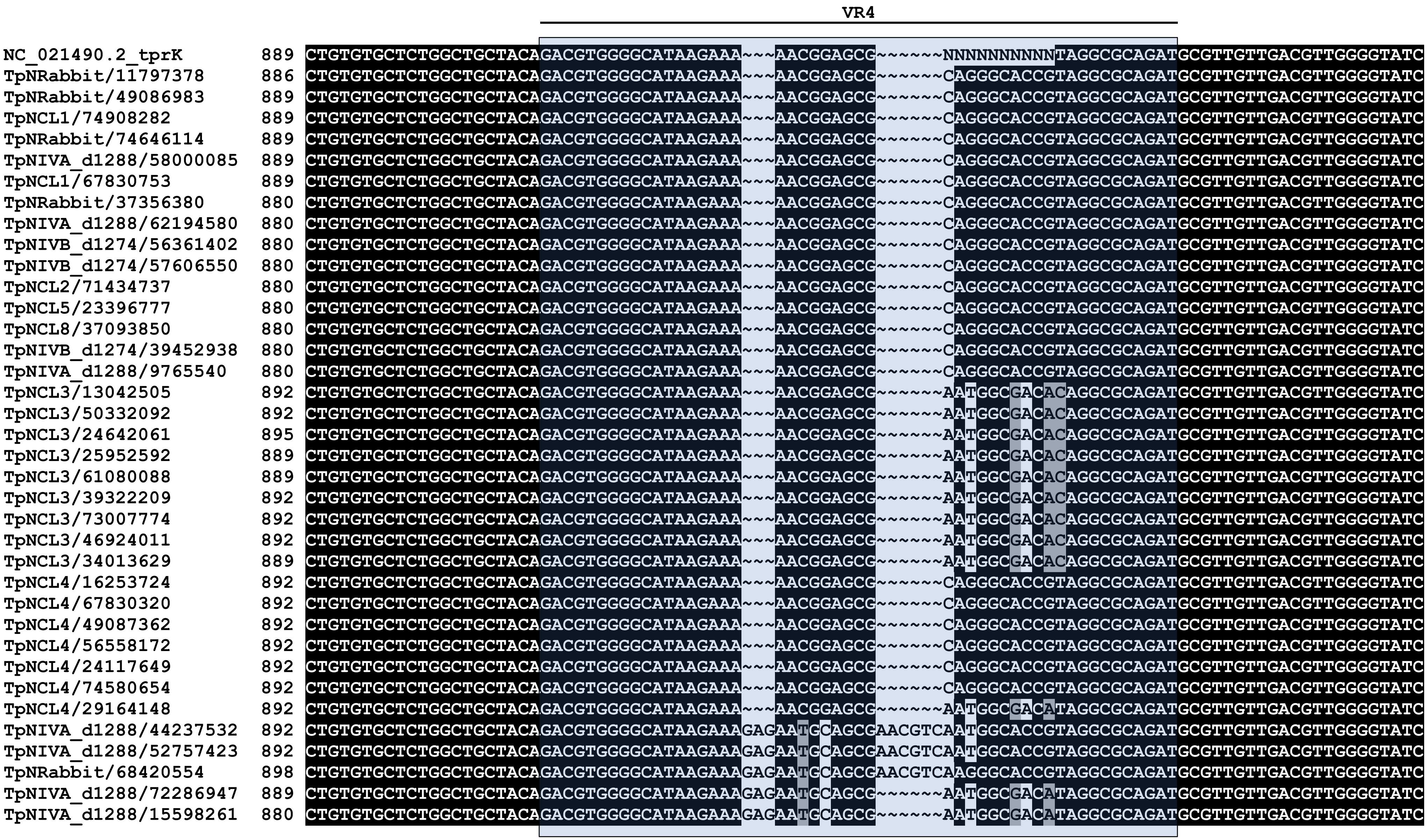

VR4
NC_021490.2_tprK 889 CTGTGTGCTCTGGCTGCTACAGACGTGGGGCATAAGAAA~~~AACGGAGCG~~~~~~NNNNNNNNNNTAGGCGCAGATGCGTTGTTGACGTTGGGGTATC
TpNRabbit/11797378 886 CTGTGTGCTCTGGCTGCTACAGACGTGGGGCATAAGAAA~~~AACGGAGCG~~~~~~CAGGGCACCGTAGGCGCAGATGCGTTGTTGACGTTGGGGTATC
TpNRabbit/49086983 889 CTGTGTGCTCTGGCTGCTACAGACGTGGGGCATAAGAAA~~~AACGGAGCG~~~~~~CAGGGCACCGTAGGCGCAGATGCGTTGTTGACGTTGGGGTATC
TpNCL1/74908282 889 CTGTGTGCTCTGGCTGCTACAGACGTGGGGCATAAGAAA~~~AACGGAGCG~~~~~~CAGGGCACCGTAGGCGCAGATGCGTTGTTGACGTTGGGGTATC
TpNRabbit/74646114 889 CTGTGTGCTCTGGCTGCTACAGACGTGGGGCATAAGAAA~~~AACGGAGCG~~~~~~CAGGGCACCGTAGGCGCAGATGCGTTGTTGACGTTGGGGTATC
TpNIVA_d1288/58000085 889 CTGTGTGCTCTGGCTGCTACAGACGTGGGGCATAAGAAA~~~AACGGAGCG~~~~~~CAGGGCACCGTAGGCGCAGATGCGTTGTTGACGTTGGGGTATC
TpNCL1/67830753 889 CTGTGTGCTCTGGCTGCTACAGACGTGGGGCATAAGAAA~~~AACGGAGCG~~~~~~CAGGGCACCGTAGGCGCAGATGCGTTGTTGACGTTGGGGTATC
TpNRabbit/37356380 880 CTGTGTGCTCTGGCTGCTACAGACGTGGGGCATAAGAAA~~~AACGGAGCG~~~~~~CAGGGCACCGTAGGCGCAGATGCGTTGTTGACGTTGGGGTATC
TpNIVA_d1288/62194580 880 CTGTGTGCTCTGGCTGCTACAGACGTGGGGCATAAGAAA~~~AACGGAGCG~~~~~~CAGGGCACCGTAGGCGCAGATGCGTTGTTGACGTTGGGGTATC
TpNIVB_d1274/56361402 880 CTGTGTGCTCTGGCTGCTACAGACGTGGGGCATAAGAAA~~~AACGGAGCG~~~~~~CAGGGCACCGTAGGCGCAGATGCGTTGTTGACGTTGGGGTATC
TpNIVB_d1274/57606550 880 CTGTGTGCTCTGGCTGCTACAGACGTGGGGCATAAGAAA~~~AACGGAGCG~~~~~~CAGGGCACCGTAGGCGCAGATGCGTTGTTGACGTTGGGGTATC
TpNCL2/71434737 880 CTGTGTGCTCTGGCTGCTACAGACGTGGGGCATAAGAAA~~~AACGGAGCG~~~~~~CAGGGCACCGTAGGCGCAGATGCGTTGTTGACGTTGGGGTATC
TpNCL5/23396777 880 CTGTGTGCTCTGGCTGCTACAGACGTGGGGCATAAGAAA~~~AACGGAGCG~~~~~~CAGGGCACCGTAGGCGCAGATGCGTTGTTGACGTTGGGGTATC
TpNCL8/37093850 880 CTGTGTGCTCTGGCTGCTACAGACGTGGGGCATAAGAAA~~~AACGGAGCG~~~~~~CAGGGCACCGTAGGCGCAGATGCGTTGTTGACGTTGGGGTATC
TpNIVB_d1274/39452938 880 CTGTGTGCTCTGGCTGCTACAGACGTGGGGCATAAGAAA~~~AACGGAGCG~~~~~~CAGGGCACCGTAGGCGCAGATGCGTTGTTGACGTTGGGGTATC
TpNIVA_d1288/9765540 880 CTGTGTGCTCTGGCTGCTACAGACGTGGGGCATAAGAAA~~~AACGGAGCG~~~~~~CAGGGCACCGTAGGCGCAGATGCGTTGTTGACGTTGGGGTATC
TpNCL3/13042505 892 CTGTGTGCTCTGGCTGCTACAGACGTGGGGCATAAGAAA~~~AACGGAGCG~~~~~~AATGGCGACACAGGCGCAGATGCGTTGTTGACGTTGGGGTATC
TpNCL3/50332092 892 CTGTGTGCTCTGGCTGCTACAGACGTGGGGCATAAGAAA~~~AACGGAGCG~~~~~~AATGGCGACACAGGCGCAGATGCGTTGTTGACGTTGGGGTATC
TpNCL3/24642061 895 CTGTGTGCTCTGGCTGCTACAGACGTGGGGCATAAGAAA~~~AACGGAGCG~~~~~~AATGGCGACACAGGCGCAGATGCGTTGTTGACGTTGGGGTATC
TpNCL3/25952592 889 CTGTGTGCTCTGGCTGCTACAGACGTGGGGCATAAGAAA~~~AACGGAGCG~~~~~~AATGGCGACACAGGCGCAGATGCGTTGTTGACGTTGGGGTATC
TpNCL3/61080088 889 CTGTGTGCTCTGGCTGCTACAGACGTGGGGCATAAGAAA~~~AACGGAGCG~~~~~~AATGGCGACACAGGCGCAGATGCGTTGTTGACGTTGGGGTATC
TpNCL3/39322209 892 CTGTGTGCTCTGGCTGCTACAGACGTGGGGCATAAGAAA~~~AACGGAGCG~~~~~~AATGGCGACACAGGCGCAGATGCGTTGTTGACGTTGGGGTATC
TpNCL3/73007774 892 CTGTGTGCTCTGGCTGCTACAGACGTGGGGCATAAGAAA~~~AACGGAGCG~~~~~~AATGGCGACACAGGCGCAGATGCGTTGTTGACGTTGGGGTATC
TpNCL3/46924011 892 CTGTGTGCTCTGGCTGCTACAGACGTGGGGCATAAGAAA~~~AACGGAGCG~~~~~~AATGGCGACACAGGCGCAGATGCGTTGTTGACGTTGGGGTATC
TpNCL3/34013629 889 CTGTGTGCTCTGGCTGCTACAGACGTGGGGCATAAGAAA~~~AACGGAGCG~~~~~~AATGGCGACACAGGCGCAGATGCGTTGTTGACGTTGGGGTATC
TpNCL4/16253724 892 CTGTGTGCTCTGGCTGCTACAGACGTGGGGCATAAGAAA~~~AACGGAGCG~~~~~~CAGGGCACCGTAGGCGCAGATGCGTTGTTGACGTTGGGGTATC
TpNCL4/67830320 892 CTGTGTGCTCTGGCTGCTACAGACGTGGGGCATAAGAAA~~~AACGGAGCG~~~~~~CAGGGCACCGTAGGCGCAGATGCGTTGTTGACGTTGGGGTATC
TpNCL4/49087362 892 CTGTGTGCTCTGGCTGCTACAGACGTGGGGCATAAGAAA~~~AACGGAGCG~~~~~~CAGGGCACCGTAGGCGCAGATGCGTTGTTGACGTTGGGGTATC
TpNCL4/56558172 892 CTGTGTGCTCTGGCTGCTACAGACGTGGGGCATAAGAAA~~~AACGGAGCG~~~~~~CAGGGCACCGTAGGCGCAGATGCGTTGTTGACGTTGGGGTATC
TpNCL4/24117649 892 CTGTGTGCTCTGGCTGCTACAGACGTGGGGCATAAGAAA~~~AACGGAGCG~~~~~~CAGGGCACCGTAGGCGCAGATGCGTTGTTGACGTTGGGGTATC
TpNCL4/74580654 892 CTGTGTGCTCTGGCTGCTACAGACGTGGGGCATAAGAAA~~~AACGGAGCG~~~~~~CAGGGCACCGTAGGCGCAGATGCGTTGTTGACGTTGGGGTATC
TpNCL4/29164148 892 CTGTGTGCTCTGGCTGCTACAGACGTGGGGCATAAGAAA~~~AACGGAGCG~~~~~~AATGGCGACATAGGCGCAGATGCGTTGTTGACGTTGGGGTATC
TpNIVA_d1288/44237532 892 CTGTGTGCTCTGGCTGCTACAGACGTGGGGCATAAGAAAGAGAATGCAGCGAACGTCAATGGCACCGTAGGCGCAGATGCGTTGTTGACGTTGGGGTATC
TpNIVA_d1288/52757423 892 CTGTGTGCTCTGGCTGCTACAGACGTGGGGCATAAGAAAGAGAATGCAGCGAACGTCAATGGCACCGTAGGCGCAGATGCGTTGTTGACGTTGGGGTATC
TpNRabbit/68420554 898 CTGTGTGCTCTGGCTGCTACAGACGTGGGGCATAAGAAAGAGAATGCAGCGAACGTCAAGGGCACCGTAGGCGCAGATGCGTTGTTGACGTTGGGGTATC
TpNIVA_d1288/72286947 889 CTGTGTGCTCTGGCTGCTACAGACGTGGGGCATAAGAAAGAGAATGCAGCG~~~~~~AATGGCGACATAGGCGCAGATGCGTTGTTGACGTTGGGGTATC
TpNIVA_d1288/15598261 880 CTGTGTGCTCTGGCTGCTACAGACGTGGGGCATAAGAAAGAGAATGCAGCG~~~~~~AATGGCGACATAGGCGCAGATGCGTTGTTGACGTTGGGGTATC

VR5

```
NC_021490.2_tprK            980 GTTGGTTCTCGGCGGGAGGATATTTCGCATCGNAGGCCAGCAATGTATTCGGGGGAGTATTTCTCAACATG~~~~~~~~~~~~~~~GCCATGCGAGAGCA
TpNRabbit/11797378          977 GTTGGTTCTCGGCGGGAGGATATTTCGCATCGAAGGCCAGCAATGTATTCGGGGGAGTATTTCTCAACATG~~~~~~~~~~~~~~~GCCATGCGAGAGCA
TpNRabbit/49086983          980 GTTGGTTCTCGGCGGGAGGATATTTCGCATCGAAGGCCAGCAATGTATTCGGGGGAGTATTTCTCAACATG~~~~~~~~~~~~~~~GCCATGCGAGAGCA
TpNCL1/74908282             980 GTTGGTTCTCGGCGGGAGGATATTTCGCATCGAAGGCCAGCAATGTATTCGGGGGAGTATTTCTCAACATG~~~~~~~~~~~~~~~GCCATGCGAGAGCA
TpNRabbit/74646114          980 GTTGGTTCTCGGCGGGAGGATATTTCGCATCGAAGGCCAGCAATGTATTCGGGGGAGTATTTCTCAACATG~~~~~~~~~~~~~~~GCCATGCGAGAGCA
TpNIVA_d1288/58000085       980 GTTGGTTCTCGGCGGGAGGATATTTCGCATCGAAGGCCAGCAATGTATTCGGGGGAGTATTTCTCAACATG~~~~~~~~~~~~~~~GCCATGCGAGAGCA
TpNCL1/67830753             980 GTTGGTTCTCGGCGGGAGGATATTTCGCATCGAAGGCCAGCAATGTATTCGGGGGAGTATTTCTCAACATG~~~~~~~~~~~~~~~GCCATGCGAGAGCA
TpNRabbit/37356380          971 GTTGGTTCTCGGCGGGAGGATATTTCGCATCGCAGGCCAGCAATGTATTCGGGGGAGTATTTCTCAACATG~~~~~~~~~~~~~~~GCCATGCGAGAGCA
TpNIVA_d1288/62194580       971 GTTGGTTCTCGGCGGGAGGATATTTCGCATCGCAGGCCAGCAATGTATTCGGGGGAGTATTTCTCAACATG~~~~~~~~~~~~~~~GCCATGCGAGAGCA
TpNIVB_d1274/56361402       971 GTTGGTTCTCGGCGGGAGGATATTTCGCATCGCAGGCCAGCAATGTATTCGGGGGAGTATTTCTCAACATG~~~~~~~~~~~~~~~GCCATGCGAGAGCA
TpNIVB_d1274/57606550       971 GTTGGTTCTCGGCGGGAGGATATTTCGCATCGCAGGCCAGCAATGTATTCGGGGGAGTATTTCTCAACATGGGGGCTGGGGGGGGGGCCATGCGAGAGCA
TpNCL2/71434737             971 GTTGGTTCTCGGCGGGAGGATATTTCGCATCGCAGGCCAGCAATGTATTCGGGGGAGTATTTCTCAACATG~~~~~~~~~~~~~~~GCCATGCGAGAGCA
TpNCL5/23396777             971 GTTGGTTCTCGGCGGGAGGATATTTCGCATCGCAGGCCAGCAATGTATTCGGGGGAGTATTTCTCAACATG~~~~~~~~~~~~~~~GCCATGCGAGAGCA
TpNCL8/37093850             971 GTTGGTTCTCGGCGGGAGGATATTTCGCATCGCAGGCCAGCAATGTATTCGGGGGAGTATTTCTCAACATG~~~~~~~~~~~~~~~GCCATGCGAGAGCA
TpNIVB_d1274/39452938       971 GTTGGTTCTCGGCGGGAGGATATTTCGCATCGCAGGCCAGCAATGTATTCGGGGGAGTATTTCTCAACATG~~~~~~~~~~~~~~~GCCATGCGAGAGCA
TpNIVA_d1288/9765540        971 GTTGGTTCTCGGCGGGAGGATATTTCGCATCGCAGGCCAGCAATGTATTCGGGGGAGTATTTCTCACATTG~~~~~~~~~~~~~~~GCCATGCGAGAGCA
TpNCL3/13042505             983 GTTGGTTCTCGGCGGGAGGATATTTCGCATCGAAGGCCAGCAATGTATTCGGGGGAGTATTTCTCAACATG~~~~~~~~~~~~~~~GCCATGCGAGAGCA
TpNCL3/50332092             983 GTTGGTTCTCGGCGGGAGGATATTTCGCATCGAAGGCCAGCAATGTATTCGGGGGAGTATTTCTCAACATG~~~~~~~~~~~~~~~GCCATGCGAGAGCA
TpNCL3/24642061             986 GTTGGTTCTCGGCGGGAGGATATTTCGCATCGAAGGCCAGCAATGTATTCGGGGGAGTATTTCTCAACATG~~~~~~~~~~~~~~~GCCATGCGAGAGCA
TpNCL3/25952592             980 GTTGGTTCTCGGCGGGAGGATATTTCGCATCGAAGGCCAGCAATGTATTCGGGGGAGTATTTCTCAACATG~~~~~~~~~~~~~~~GCCATGCGAGAGCA
TpNCL3/61080088             980 GTTGGTTCTCGGCGGGAGGATATTTCGCATCGAAGGCCAGCAATGTATTCGGGGGAGTATTTCTCAACATG~~~~~~~~~~~~~~~GCCATGCGAGAGCA
TpNCL3/39322209             983 GTTGGTTCTCGGCGGGAGGATATTTCGCATCGAAGGCCAGCAATGTATTCGGGGGAGTATTTCTCAACATG~~~~~~~~~~~~~~~GCCATGCGAGAGCA
TpNCL3/73007774             983 GTTGGTTCTCGGCGGGAGGATATTTCGCATCGAAGGCCAGCAATGTATTCGGGGGAGTATTTCTCACCAAT~~~~~~~~~~~~~~~AACATGCTGCAGCA
TpNCL3/46924011             983 GTTGGTTCTCGGCGGGAGGATATTTCGCATCGAAGGCCAGCAATGTATTCGGGGGAGTATTTCTCAACATG~~~~~~~~~~~~~~~GCCATGCGAGAGCA
TpNCL3/34013629             980 GTTGGTTCTCGGCGGGAGGATATTTCGCATCGAAGGCCAGCAATGTGTTTAAAGACGTCTTTCTCACCAAT~~~~~~~~~~~~~~~AACATGCTGCAGCA
TpNCL4/16253724             983 GTTGGTTCTCGGCGGGAGGATATTTCGCATCGAAGGCCAGCAATGTATTTCAGGGTGTCTTTCTCAACATG~~~~~~~~~~~~~~~GCCATGCGAGAGCA
TpNCL4/67830320             983 GTTGGTTCTCGGCGGGAGGATATTTCGCATCGAAGGCCAGCAATGTATTTCAGGGTGTCTTTCTCAACATG~~~~~~~~~~~~~~~GCCATGCGAGAGCA
TpNCL4/49087362             983 GTTGGTTCTCGGCGGGAGGATATTTCGCATCGAAGGCCAGCAATGTATTTCAGGGTGTCTTTCTCAACATG~~~~~~~~~~~~~~~GCCATGCGAGAGCA
TpNCL4/56558172             983 GTTGGTTCTCGGCGGGAGGATATTTCGCATCGAAGGCCAGCAATGTATTTCAGGGTGTCTTTCTCACCACA~~~~~~~~~~~~~~~CCCATGCAGAAGCA
TpNCL4/24117649             983 GTTGGTTCTCGGCGGGAGGATATTTCGCATCGCAGGCCAGCAATGTATTTCAGGGTGTCTTTCTCAACATG~~~~~~~~~~~~~~~GCCATGCGAGAGCA
TpNCL4/74580654             983 GTTGGTTCTCGGCGGGAGGATATTTCGCATCGAAGGCCAGCAATGTATTTCAGGGTGTCTTTCTCAACATG~~~~~~~~~~~~~~~GCCATGCGAGAGCA
TpNCL4/29164148             983 GTTGGTTCTCGGCGGGAGGATATTTCGCATCGAAGGCCAGCAATGTATTTCAGGGTGTCTTTCTCAACATG~~~~~~~~~~~~~~~GCCATGCGAGAGCA
TpNIVA_d1288/44237532       992 GTTGGTTCTCGGCGGGAGGATATTTCGCATCGAAGGCCAGCAATGTATTCGAGGGAGTATTTCTCAACATG~~~~~~~~~~~~~~~GCCATGACCGCACA
TpNIVA_d1288/52757423       992 GTTGGTTCTCGGCGGGAGGATATTTCGCATCGAAGGCCAGCAATGTATTTCAGGGAGTATTTCTCAACATG~~~~~~~~~~~~~~~GCCATGACCGCACA
TpNRabbit/68420554          998 GTTGGTTCTCGGCGGGAGGATATTTCGCATCGAAGGCCAGCAATGTATTCGGGGGAGTATTTCTCAACATG~~~~~~~~~~~~~~~GCCATGCGAGAGCA
TpNIVA_d1288/72286947       983 GTTGGTTCTCGGCGGGAGGATATTTCGCATCGAAGGCCAGCAATGTGTTTAAAGACGTCTTTCTCACCAAT~~~~~~~~~GCCATGGACATGCAGACGCA
TpNIVA_d1288/15598261       974 GTTGGTTCTCGGCGGGAGGATATTTCGCATCGAAGGCCAGCAATGTATTTCAGGGAGTATTTCTCAACATG~~~~~~~~~~~~~~~GCCATGACCGCACA
```

```
                             VR5
                             ____________________________
CP004010.2_tprK           1065 CGACTGTGCTGCCTATATTAAGCTCGAAACCAAGGGGTCTGATCCTGATACTTCTTTCCTTGAGGGTCTTGATTTGGGTGTTGATGTGCGTACGTACATG
TpNRabbit/11797378        1062 CGACTGTGCTGCCTATATTAAGCTCGAAACCAAGGGGTCTGATCCTGATACTTCTTTCCTTGAGGGTCTTGATTTGGGTGTTGATGTGCGTACGTACATG
TpNRabbit/49086983        1065 CGACTGTGCTGCCTATATTAAGCTCGAAACCAAGGGGTCTGATCCTGATACTTCTTTCCTTGAGGGTCTTGATTTGGGTGTTGATGTGCGTACGTACATG
TpNCL1/74908282           1065 CGACTGTGCTGCCTATATTAAGCTCGAAACCAAGGGGTCTGATCCTGATACTTCTTTCCTTGAGGGTCTTGATTTGGGTGTTGATGTGCGTACGTACATG
TpNRabbit/74646114        1065 CGACTGTGCTGCCTATATTAAGCTCGAAACCAAGGGGTCTGATCCTGATACTTCTTTCCTTGAGGGTCTTGATTTGGGTGTTGATGTGCGTACGTACATG
TpNIVA_d1288/58000085     1065 CGACTGTGCTGCCTATATTAAGCTCGAAACCAAGGGGTCTGATCCTGATACTTCTTTCCTTGAGGGTCTTGATTTGGGTGTTGATGTGCGTACGTACATG
TpNCL1/67830753           1065 CGACTGTGCTGCCTATATTAAGCTCGAAACCAAGGGGTCTGATCCTGATACTTCTTTCCTTGAGGGTCTTGATTTGGGTGTTGATGTGCGTACGTACATG
TpNRabbit/37356380        1056 CGACTGTGCTGCCTATATTAAGCTCGAAACCAAGGGGTCTGATCCTGATACTTCTTTCCTTGAGGGTCTTGATTTGGGTGTTGATGTGCGTACGTACATG
TpNIVA_d1288/62194580     1056 CGACTGTGCTGCCTATATTAAGCTCGAAACCAAGGGGTCTGATCCTGATACTTCTTTCCTTGAGGGTCTTGATTTGGGTGTTGATGTGCGTACGTACATG
TpNIVB_d1274/56361402     1056 CGACTGTGCTGCCTATATTAAGCTCGAAACCAAGGGGTCTGATCCTGATACTTCTTTCCTTGAGGGTCTTGATTTGGGTGTTGATGTGCGTACGTACATG
TpNIVB_d1274/57606550     1071 CGACTGTGCTGCCTATATTAAGCTCGAAACCAAGGGGTCTGATCCTGATACTTCTTTCCTTGAGGGTCTTGATTTGGGTGTTGATGTGCGTACGTACATG
TpNCL2/71434737           1056 CGACTGTGCTGCCTATATTAAGCTCGAAACCAAGGGGTCTGATCCTGATACTTCTTTCCTTGAGGGTCTTGATTTGGGTGTTGATGTGCGTACGTACATG
TpNCL5/23396777           1056 CGACTGTGCTGCCTATATTAAGCTCGAAACCAAGGGGTCTGATCCTGATACTTCTTTCCTTGAGGGTCTTGATTTGGGTGTTGATGTGCGTACGTACATG
TpNCL8/37093850           1056 CGACTGTGCTGCCTATATTAAGCTCGAAACCAAGGGGTCTGATCCTGATACTTCTTTCCTTGAGGGTCTTGATTTGGGTGTTGATGTGCGTACGTACATG
TpNIVB_d1274/39452938     1056 CGACTGTGCTGCCTATATTAAGCTCGAAACCAAGGGGTCTGATCCTGATACTTCTTTCCTTGAGGGTCTTGATTTGGGTGTTGATGTGCGTACGTACATG
TpNIVA_d1288/9765540      1056 CGACTGTGCTGCCTATATTAAGCTCGAAACCAAGGGGTCTGATCCTGATACTTCTTTCCTTGAGGGTCTTGATTTGGGTGTTGATGTGCGTACGTACATG
TpNCL3/13042505           1068 CGACTGTGCTGCCTATATTAAGCTCGAAACCAAGGGGTCTGATCCTGATACTTCTTTCCTTGAGGGTCTTGATTTGGGTGTTGATGTGCGTACGTACATG
TpNCL3/50332092           1068 CGACTGTGCTGCCTATATTAAGCTCGAAACCAAGGGGTCTGATCCTGATACTTCTTTCCTTGAGGGTCTTGATTTGGGTGTTGATGTGCGTACGTACATG
TpNCL3/24642061           1071 CGACTGTGCTGCCTATATTAAGCTCGAAACCAAGGGGTCTGATCCTGATACTTCTTTCCTTGAGGGTCTTGATTTGGGTGTTGATGTGCGTACGTACATG
TpNCL3/25952592           1065 CGACTGTGCTGCCTATATTAAGCTCGAAACCAAGGGGTCTGATCCTGATACTTCTTTCCTTGAGGGTCTTGATTTGGGTGTTGATGTGCGTACGTACATG
TpNCL3/61080088           1065 CGACTGTGCTGCCTATATTAAGCTCGAAACCAAGGGGTCTGATCCTGATACTTCTTTCCTTGAGGGTCTTGATTTGGGTGTTGATGTGCGTACGTACATG
TpNCL3/39322209           1068 CGACTGTGCTGCCTATATTAAGCTCGAAACCAAGGGGTCTGATCCTGATACTTCTTTCCTTGAGGGTCTTGATTTGGGTGTTGATGTGCGTACGTACATG
TpNCL3/73007774           1068 CGACTGTGCTGCCTATATTAAGCTCGAAACCAAGGGGTCTGATCCTGATACTTCTTTCCTTGAGGGTCTTGATTTGGGTGTTGATGTGCGTACGTACATG
TpNCL3/46924011           1068 CGACTGTGCTGCCTATATTAAGCTCGAAACCAAGGGGTCTGATCCTGATACTTCTTTCCTTGAGGGTCTTGATTTGGGTGTTGATGTGCGTACGTACATG
TpNCL3/34013629           1065 CGACTGTGCTGCCTATATTAAGCTCGAAACCAAGGGGTCTGATCCTGATACTTCTTTCCTTGAGGGTCTTGATTTGGGTGTTGATGTGCGTACGTACATG
TpNCL4/16253724           1068 CGACTGTGCTGCCTATATTAAGCTCGAAACCAAGGGGTCTGATCCTGATACTTCTTTCCTTGAGGGTCTTGATTTGGGTGTTGATGTGCGTACGTACATG
TpNCL4/67830320           1068 CGACTGTGCTGCCTATATTAAGCTCGAAACCAAGGGGTCTGATCCTGATACTTCTTTCCTTGAGGGTCTTGATTTGGGTGTTGATGTGCGTACGTACATG
TpNCL4/49087362           1068 CGACTGTGCTGCCTATATTAAGCTCGAAACCAAGGGGTCTGATCCTGATACTTCTTTCCTTGAGGGTCTTGATTTGGGTGTTGATGTGCGTACGTACATG
TpNCL4/56558172           1068 CGACTGTGCTGCCTATATTAAGCTCGAAACCAAGGGGTCTGATCCTGATACTTCTTTCCTTGAGGGTCTTGATTTGGGTGTTGATGTGCGTACGTACATG
TpNCL4/24117649           1068 CGACTGTGCTGCCTATATTAAGCTCGAAACCAAGGGGTCTGATCCTGATACTTCTTTCCTTGAGGGTCTTGATTTGGGTGTTGATGTGCGTACGTACATG
TpNCL4/74580654           1068 CGACTGTGCTGCCTATATTAAGCTCGAAACCAAGGGGTCTGATCCTGATACTTCTTTCCTTGAGGGTCTTGATTTGGGTGTTGATGTGCGTACGTACATG
TpNCL4/29164148           1068 CGACTGTGCTGCCTATATTAAGCTCGAAACCAAGGGGTCTGATCCTGATACTTCTTTCCTTGAGGGTCTTGATTTGGGTGTTGATGTGCGTACGTACATG
TpNIVA_d1288/44237532     1077 CGACTGTGCTGCCTATATTAAGCTCGAAACCAAGGGGTCTGATCCTGATACTTCTTTCCTTGAGGGTCTTGATTTGGGTGTTGATGTGCGTACGTACATG
TpNIVA_d1288/52757423     1077 CGACTGTGCTGCCTATATTAAGCTCGAAACCAAGGGGTCTGATCCTGATACTTCTTTCCTTGAGGGTCTTGATTTGGGTGTTGATGTGCGTACGTACATG
TpNRabbit/68420554        1083 CGACTGTGCTGCCTATATTAAGCTCGAAACCAAGGGGTCTGATCCTGATACTTCTTTCCTTGAGGGTCTTGATTTGGGTGTTGATGTGCGTACGTACATG
TpNIVA_d1288/72286947     1074 CGACTGTGCTGCCTATATTAAGCTCGAAACCAAGGGGTCTGATCCTGATACTTCTTTCCTTGAGGGTCTTGATTTGGGTGTTGATGTGCGTACGTACATG
TpNIVA_d1288/15598261     1059 CGACTGTGCTGCCTATATTAAGCTCGAAACCAAGGGGTCTGATCCTGATACTTCTTTCCTTGAGGGTCTTGATTTGGGTGTTGATGTGCGTACGTACATG
```

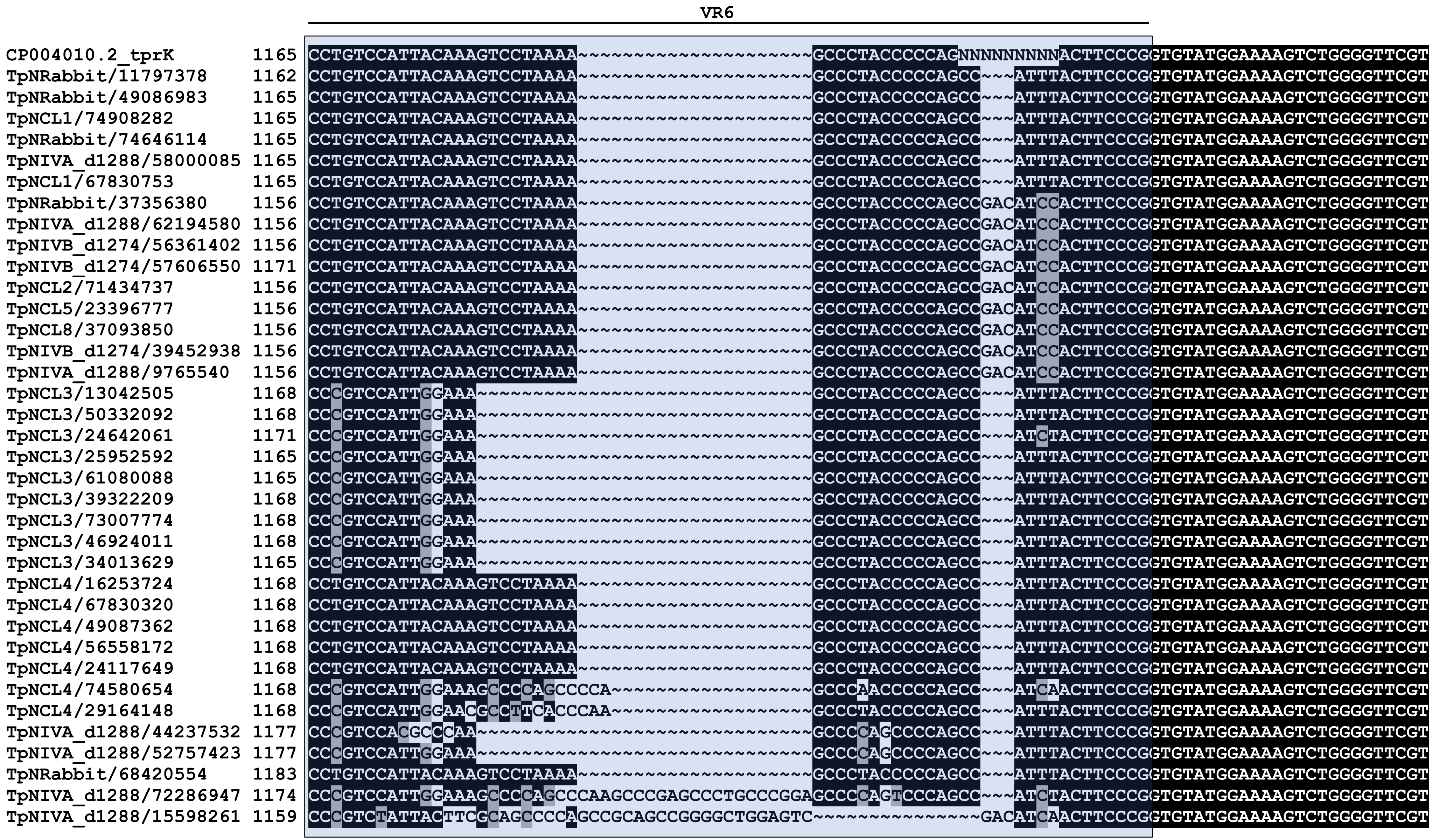
VR6
CP004010.2_tprK 1165 CCTGTCCATTACAAAGTCCTAAAA~~~~~~~~~~~~~~~~~~~~~GCCCTACCCCCAGNNNNNNNNNACTTCCCGGTGTATGGAAAAGTCTGGGGTTCGT
TpNRabbit/11797378 1162 CCTGTCCATTACAAAGTCCTAAAA~~~~~~~~~~~~~~~~~~~~~GCCCTACCCCCAGCC~~~ATTTACTTCCCGGTGTATGGAAAAGTCTGGGGTTCGT
TpNRabbit/49086983 1165 CCTGTCCATTACAAAGTCCTAAAA~~~~~~~~~~~~~~~~~~~~~GCCCTACCCCCAGCC~~~ATTTACTTCCCGGTGTATGGAAAAGTCTGGGGTTCGT
TpNCL1/74908282 1165 CCTGTCCATTACAAAGTCCTAAAA~~~~~~~~~~~~~~~~~~~~~GCCCTACCCCCAGCC~~~ATTTACTTCCCGGTGTATGGAAAAGTCTGGGGTTCGT
TpNRabbit/74646114 1165 CCTGTCCATTACAAAGTCCTAAAA~~~~~~~~~~~~~~~~~~~~~GCCCTACCCCCAGCC~~~ATTTACTTCCCGGTGTATGGAAAAGTCTGGGGTTCGT
TpNIVA_d1288/58000085 1165 CCTGTCCATTACAAAGTCCTAAAA~~~~~~~~~~~~~~~~~~~~~GCCCTACCCCCAGCC~~~ATTTACTTCCCGGTGTATGGAAAAGTCTGGGGTTCGT
TpNCL1/67830753 1165 CCTGTCCATTACAAAGTCCTAAAA~~~~~~~~~~~~~~~~~~~~~GCCCTACCCCCAGCC~~~ATTTACTTCCCGGTGTATGGAAAAGTCTGGGGTTCGT
TpNRabbit/37356380 1156 CCTGTCCATTACAAAGTCCTAAAA~~~~~~~~~~~~~~~~~~~~~GCCCTACCCCCAGCCGACATCCACTTCCCGGTGTATGGAAAAGTCTGGGGTTCGT
TpNIVA_d1288/62194580 1156 CCTGTCCATTACAAAGTCCTAAAA~~~~~~~~~~~~~~~~~~~~~GCCCTACCCCCAGCCGACATCCACTTCCCGGTGTATGGAAAAGTCTGGGGTTCGT
TpNIVB_d1274/56361402 1156 CCTGTCCATTACAAAGTCCTAAAA~~~~~~~~~~~~~~~~~~~~~GCCCTACCCCCAGCCGACATCCACTTCCCGGTGTATGGAAAAGTCTGGGGTTCGT
TpNIVB_d1274/57606550 1171 CCTGTCCATTACAAAGTCCTAAAA~~~~~~~~~~~~~~~~~~~~~GCCCTACCCCCAGCCGACATCCACTTCCCGGTGTATGGAAAAGTCTGGGGTTCGT
TpNCL2/71434737 1156 CCTGTCCATTACAAAGTCCTAAAA~~~~~~~~~~~~~~~~~~~~~GCCCTACCCCCAGCCGACATCCACTTCCCGGTGTATGGAAAAGTCTGGGGTTCGT
TpNCL5/23396777 1156 CCTGTCCATTACAAAGTCCTAAAA~~~~~~~~~~~~~~~~~~~~~GCCCTACCCCCAGCCGACATCCACTTCCCGGTGTATGGAAAAGTCTGGGGTTCGT
TpNCL8/37093850 1156 CCTGTCCATTACAAAGTCCTAAAA~~~~~~~~~~~~~~~~~~~~~GCCCTACCCCCAGCCGACATCCACTTCCCGGTGTATGGAAAAGTCTGGGGTTCGT
TpNIVB_d1274/39452938 1156 CCTGTCCATTACAAAGTCCTAAAA~~~~~~~~~~~~~~~~~~~~~GCCCTACCCCCAGCCGACATCCACTTCCCGGTGTATGGAAAAGTCTGGGGTTCGT
TpNIVA_d1288/9765540 1156 CCTGTCCATTACAAAGTCCTAAAA~~~~~~~~~~~~~~~~~~~~~GCCCTACCCCCAGCCGACATCCACTTCCCGGTGTATGGAAAAGTCTGGGGTTCGT
TpNCL3/13042505 1168 CCCGTCCATTGGAAA~~~~~~~~~~~~~~~~~~~~~~~~~~~~~~GCCCTACCCCCAGCC~~~ATTTACTTCCCGGTGTATGGAAAAGTCTGGGGTTCGT
TpNCL3/50332092 1168 CCCGTCCATTGGAAA~~~~~~~~~~~~~~~~~~~~~~~~~~~~~~GCCCTACCCCCAGCC~~~ATTTACTTCCCGGTGTATGGAAAAGTCTGGGGTTCGT
TpNCL3/24642061 1171 CCCGTCCATTGGAAA~~~~~~~~~~~~~~~~~~~~~~~~~~~~~~GCCCTACCCCCAGCC~~~ATCTACTTCCCGGTGTATGGAAAAGTCTGGGGTTCGT
TpNCL3/25952592 1165 CCCGTCCATTGGAAA~~~~~~~~~~~~~~~~~~~~~~~~~~~~~~GCCCTACCCCCAGCC~~~ATTTACTTCCCGGTGTATGGAAAAGTCTGGGGTTCGT
TpNCL3/61080088 1165 CCCGTCCATTGGAAA~~~~~~~~~~~~~~~~~~~~~~~~~~~~~~GCCCTACCCCCAGCC~~~ATTTACTTCCCGGTGTATGGAAAAGTCTGGGGTTCGT
TpNCL3/39322209 1168 CCCGTCCATTGGAAA~~~~~~~~~~~~~~~~~~~~~~~~~~~~~~GCCCTACCCCCAGCC~~~ATTTACTTCCCGGTGTATGGAAAAGTCTGGGGTTCGT
TpNCL3/73007774 1168 CCCGTCCATTGGAAA~~~~~~~~~~~~~~~~~~~~~~~~~~~~~~GCCCTACCCCCAGCC~~~ATTTACTTCCCGGTGTATGGAAAAGTCTGGGGTTCGT
TpNCL3/46924011 1168 CCCGTCCATTGGAAA~~~~~~~~~~~~~~~~~~~~~~~~~~~~~~GCCCTACCCCCAGCC~~~ATTTACTTCCCGGTGTATGGAAAAGTCTGGGGTTCGT
TpNCL3/34013629 1165 CCCGTCCATTGGAAA~~~~~~~~~~~~~~~~~~~~~~~~~~~~~~GCCCTACCCCCAGCC~~~ATTTACTTCCCGGTGTATGGAAAAGTCTGGGGTTCGT
TpNCL4/16253724 1168 CCTGTCCATTACAAAGTCCTAAAA~~~~~~~~~~~~~~~~~~~~~GCCCTACCCCCAGCC~~~ATTTACTTCCCGGTGTATGGAAAAGTCTGGGGTTCGT
TpNCL4/67830320 1168 CCTGTCCATTACAAAGTCCTAAAA~~~~~~~~~~~~~~~~~~~~~GCCCTACCCCCAGCC~~~ATTTACTTCCCGGTGTATGGAAAAGTCTGGGGTTCGT
TpNCL4/49087362 1168 CCTGTCCATTACAAAGTCCTAAAA~~~~~~~~~~~~~~~~~~~~~GCCCTACCCCCAGCC~~~ATTTACTTCCCGGTGTATGGAAAAGTCTGGGGTTCGT
TpNCL4/56558172 1168 CCTGTCCATTACAAAGTCCTAAAA~~~~~~~~~~~~~~~~~~~~~GCCCTACCCCCAGCC~~~ATTTACTTCCCGGTGTATGGAAAAGTCTGGGGTTCGT
TpNCL4/24117649 1168 CCTGTCCATTACAAAGTCCTAAAA~~~~~~~~~~~~~~~~~~~~~GCCCTACCCCCAGCC~~~ATTTACTTCCCGGTGTATGGAAAAGTCTGGGGTTCGT
TpNCL4/74580654 1168 CCCGTCCATTGGAAAGCCCAGCCCCA~~~~~~~~~~~~~~~~~~GCCCAACCCCCAGCC~~~ATCAACTTCCCGGTGTATGGAAAAGTCTGGGGTTCGT
TpNCL4/29164148 1168 CCCGTCCATTGGAACGCCTTCACCCAA~~~~~~~~~~~~~~~~~GCCCTACCCCCAGCC~~~ATTTACTTCCCGGTGTATGGAAAAGTCTGGGGTTCGT
TpNIVA_d1288/44237532 1177 CCCGTCCACGCCCAA~~~~~~~~~~~~~~~~~~~~~~~~~~~~~~GCCCCAGCCCCAGCC~~~ATTTACTTCCCGGTGTATGGAAAAGTCTGGGGTTCGT
TpNIVA_d1288/52757423 1177 CCCGTCCATTGGAAA~~~~~~~~~~~~~~~~~~~~~~~~~~~~~~GCCCCAGCCCCAGCC~~~ATTTACTTCCCGGTGTATGGAAAAGTCTGGGGTTCGT
TpNRabbit/68420554 1183 CCTGTCCATTACAAAGTCCTAAAA~~~~~~~~~~~~~~~~~~~~~GCCCTACCCCCAGCC~~~ATTTACTTCCCGGTGTATGGAAAAGTCTGGGGTTCGT
TpNIVA_d1288/72286947 1174 CCCGTCCATTGGAAAGCCCAGCCCAAGCCCGAGCCCTGCCCGGAGCCCCAGTCCCAGCC~~~ATCTACTTCCCGGTGTATGGAAAAGTCTGGGGTTCGT
TpNIVA_d1288/15598261 1159 CCCGTCTATTACTTCGCAGCCCCAGCCGCAGCCGGGGCTGGAGTC~~~~~~~~~~~~~~~GACATCAACTTCCCGGTGTATGGAAAAGTCTGGGGTTCGT

```
                                                                                                       VR7
CP004010.2_tprK            1244 ATCGTCATGATATGGGTGAGTATGGTTGGGTTAAAGTGTATGCAAACTTGTACGGCGGTACGAACAAAAAGGCC~~~~~~~~~~~~~~~ACGCCCCCTGC
TpNRabbit/11797378         1238 ATCGTCATGATATGGGTGAGTATGGTTGGGTTAAAGTGTATGCAAACTTGTACGGCGGTACGAACAAAAAGGCC~~~~~~~~~~~~~~~ACGCCCCCTGC
TpNRabbit/49086983         1241 ATCGTCATGATATGGGTGAGTATGGTTGGGTTAAAGTGTATGCAAACTTGTACGGCGGTACGAACAAAAAGGCC~~~~~~~~~~~~~~~ACGCCCCCTGC
TpNCL1/74908282            1241 ATCGTCATGATATGGGTGAGTATGGTTGGGTTAAAGTGTATGCAAACTTGTACGGCGGTACGAACAAAAAGGCC~~~~~~~~~~~~~~~ACGCCCCCTGC
TpNRabbit/74646114         1241 ATCGTCATGATATGGGTGAGTATGGTTGGGTTAAAGTGTATGCAAACTTGTACGGCGGTACGAACAAAAAGGCC~~~~~~~~~~~~~~~ACGCCCCCTGC
TpNIVA_d1288/58000085      1241 ATCGTCATGATATGGGTGAGTATGGTTGGGTTAAAGTGTATGCAAACTTGTACGGCGGTACGAACAAAAAGGCC~~~~~~~~~~~~~~~ACGCCCCCTGC
TpNCL1/67830753            1241 ATCGTCATGATATGGGTGAGTATGGTTGGGTTAAAGTGTATGCAAACTTGTACGGCGGTACGAACAAAAAGGCC~~~~~~~~~~~~~~~ACGCCCCCTGC
TpNRabbit/37356380         1235 ATCGTCATGATATGGGTGAGTATGGTTGGGTTAAAGTGTATGCAAACTTGTACGGCGGTACGAACAAAAAGGCC~~~~~~~~~~~~~~~ACGCCCCCTGC
TpNIVA_d1288/62194580      1235 ATCGTCATGATATGGGTGAGTATGGTTGGGTTAAAGTGTATGCAAACTTGTACGGCGGTACGAACAAAAAGGCC~~~~~~~~~~~~~~~ACGCCCCCTGC
TpNIVB_d1274/56361402      1235 ATCGTCATGATATGGGTGAGTATGGTTGGGTTAAAGTGTATGCAAACTTGTACGGCGGTACGAACAAAAAGGCC~~~~~~~~~~~~~~~ACGCCCCCTGC
TpNIVB_d1274/57606550      1250 ATCGTCATGATATGGGTGAGTATGGTTGGGTTAAAGTGTATGCAAACTTGTACGGCGGTACGAACAAAAAGGCC~~~~~~~~~~~~~~~ACGCCCCCTGC
TpNCL2/71434737            1235 ATCGTCATGATATGGGTGAGTATGGTTGGGTTAAAGTGTATGCAAACTTGTACGGCGGTACGAACAAAAAGGCC~~~~~~~~~~~~~~~ACGCCCCCTGC
TpNCL5/23396777            1235 ATCGTCATGATATGGGTGAGTATGGTTGGGTTAAAGTGTATGCAAACTTGTACGGCGGTACGAACAAAAAGGCC~~~~~~~~~~~~~~~ACGCCCCCTGC
TpNCL8/37093850            1235 ATCGTCATGATATGGGTGAGTATGGTTGGGTTAAAGTGTATGCAAACTTGTACGGCGGTACGAACAAAAAGGCC~~~~~~~~~~~~~~~ACGCCCCCTGC
TpNIVB_d1274/39452938      1235 ATCGTCATGATATGGGTGAGTATGGTTGGGTTAAAGTGTATGCAAACTTGTACGGCGGTACGAACAAAAAGGCC~~~~~~~~~~~~~~~ACGCCCCCTGC
TpNIVA_d1288/9765540       1235 ATCGTCATGATATGGGTGAGTATGGTTGGGTTAAAGTGTATGCAAACTTGTACGGCGGTACGAACAAAAAGGCC~~~~~~~~~~~~~~~ACGCCCCCTGC
TpNCL3/13042505            1235 ATCGTCATGATATGGGTGAGTATGGTTGGGTTAAAGTGTATGCAAACTTGTACGGCGGTACGAACAAAAAGGCC~~~~~~~~~~~~~~~ACGCCCCCTGC
TpNCL3/50332092            1235 ATCGTCATGATATGGGTGAGTATGGTTGGGTTAAAGTGTATGCAAACTTGTACGGCGGTACGAACAAAAAGGCC~~~~~~~~~~~~~~~ACGCCCCCTGC
TpNCL3/24642061            1238 ATCGTCATGATATGGGTGAGTATGGTTGGGTTAAAGTGTATGCAAACTTGTACGGCGGTACGAACAAAAAGGCC~~~~~~~~~~~~~~~ACGCCCCCTGC
TpNCL3/25952592            1232 ATCGTCATGATATGGGTGAGTATGGTTGGGTTAAAGTGTATGCAAACTTGTACGGCGGTACGAACAAAAAGGCC~~~~~~~~~~~~~~~ACGCCCCCTGC
TpNCL3/61080088            1232 ATCGTCATGATATGGGTGAGTATGGTTGGGTTAAAGTGTATGCAAACTTGTACGGCGGTACGAACAAAAAGGCC~~~~~~~~~~~~~~~ACGCCCCCTGC
TpNCL3/39322209            1235 ATCGTCATGATATGGGTGAGTATGGTTGGGTTAAAGTGTATGCAAACTTGTACGGCGGTACGAACAAAAAGGCC~~~~~~~~~~~~~~~ACGCCCCCTGC
TpNCL3/73007774            1235 ATCGTCATGATATGGGTGAGTATGGTTGGGTTAAAGTGTATGCAAACTTGTACGGCGGTACGAACAAAAAGGCC~~~~~~~~~~~~~~~ACGCCCCCTGC
TpNCL3/46924011            1235 ATCGTCATGATATGGGTGAGTATGGTTGGGTTAAAGTGTATGCAAACTTGTACGGCGGTACGAACAAGAAAAACGAT~~~~~~~~~~~~~~~~~~~~~GC
TpNCL3/34013629            1232 ATCGTCATGATATGGGTGAGTATGGTTGGGTTAAAGTGTATGCAAACTTGTACGGCGGTACGAACAAAAAGGCC~~~~~~~~~~~~~~~ACGCCCCCTGC
TpNCL4/16253724            1244 ATCGTCATGATATGGGTGAGTATGGTTGGGTTAAAGTGTATGCAAACTTGTACGGCGGTACGAACAAGCAAGCTGCTGCGGTCCCCGGCGTCGTCCCTGC
TpNCL4/67830320            1244 ATCGTCATGATATGGGTGAGTATGGTTGGGTTAAAGTGTATGCAAACTTGTACGGCGGTACGAACAAGCAAGCTGCTGCGGTCCCCGGCGTCGTCCCTGC
TpNCL4/49087362            1244 ATCGTCATGATATGGGTGAGTATGGTTGGGTTAAAGTGTATGCAAACTTGTACGGCGGTACGAACAAGCAAGCTGCTGCGGTCCCCGGCGTCGTCCCTGC
TpNCL4/56558172            1244 ATCGTCATGATATGGGTGAGTATGGTTGGGTTAAAGTGTATGCAAACTTGTACGGCGGTACGAACAAGCAAGCTGCTGCGGTCCCCGGCGTCGTCCCTGC
TpNCL4/24117649            1244 ATCGTCATGATATGGGTGAGTATGGTTGGGTTAAAGTGTATGCAAACTTGTACGGCGGTACGAACAAGCAAGCTGCTGCGGTCCCCGGCGTCGTCCCTGC
TpNCL4/74580654            1247 ATCGTCATGATATGGGTGAGTATGGTTGGGTTAAAGTGTATGCAAACTTGTACGGCGGTACGAACAAGCAAGCTGCTGCGGTCCCCGGCGTCGTCCCTGC
TpNCL4/29164148            1247 ATCGTCATGATATGGGTGAGTATGGTTGGGTTAAAGTGTATGCAAACTTGTACGGCGGTACGAACAAGCAAGCTGCTGCGGTCCCCGGCGTCGTCCCTGC
TpNIVA_d1288/44237532      1244 ATCGTCATGATATGGGTGAGTATGGTTGGGTTAAAGTGTATGCAAACTTGTACGGCGGTACGAACAAAAAGGCC~~~~~~~~~~~~~~~ACGCCCCCTGC
TpNIVA_d1288/52757423      1244 ATCGTCATGATATGGGTGAGTATGGTTGGGTTAAAGTGTATGCAAACTTGTACGGCGGTACGAACAAAAAGGCC~~~~~~~~~~~~~~~ACGCCCCCTGC
TpNRabbit/68420554         1259 ATCGTCATGATATGGGTGAGTATGGTTGGGTTAAAGTGTATGCAAACTTGTACGGCGGTACGAACAAAAAGGCC~~~~~~~~~~~~~~~ACGCCCCCTGC
TpNIVA_d1288/72286947      1271 ATCGTCATGATATGGGTGAGTATGGTTGGGTTAAAGTGTATGCAAACTTGTACGGCGGTACGAACAAGAAAAACGAT~~~~~~~~~~~~~~~~~~~~~~~
TpNIVA_d1288/15598261      1244 ATCGTCATGATATGGGTGAGTATGGTTGGGTTAAAGTGTATGCAAACTTGTACGGCGGTACGAACAAAAAGGCC~~~~~~~~~~~~~~~ACGCCCCCT~~
```

```
                                                    VR7
CP004010.2_tprK            1329 TGCTCCTGCT~~~~~~ACGAAGTGGANNNNNNNATATTGTGGGTATTACGAGTGTGGGGTAGTGGTCAGTCCGTTAGAGAAGGTGGAGATTCGGCTGAGC
TpNRabbit/11797378         1323 TGCTCCTGCTCTT~~~ACGAAGTGGAAGGCAGAATATTGTGGGTATTACGAGTGTGGGGTAGTGGTCAGTCCGTTAGAGAAGGTGGAGATTCGGCTGAGC
TpNRabbit/49086983         1326 TGCTCCTGCT~~~~~~ACGAAGTGGAAGGCAGGATATTGTGGGTATTACGAGTGTGGGGTAGTGGTCAGTCCGTTAGAGAAGGTGGAGATTCGGCTGAGC
TpNCL1/74908282            1326 TGCTCCTGCT~~~~~~ACGAAGTGGAAGGCAGGATATTGTGGGTATTACGAGTGTGGGGTAGTGGTCAGTCCGTTAGAGAAGGTGGAGATTCGGCTGAGC
TpNRabbit/74646114         1326 TGCTCCTGCT~~~~~~ACGAAGTGGAGCAAGGAATATTGTGGGTATTACGAGTGTGGGGTAGTGGTCAGTCCGTTAGAGAAGGTGGAGATTCGGCTGAGC
TpNIVA_d1288/58000085      1326 TGCTCCTGCT~~~~~~ACGAAGTGGAGCAAGGAATATTGTGGGTATTACGAGTGTGGGGTAGTGGTCAGTCCGTTAGAGAAGGTGGAGATTCGGCTGAGC
TpNCL1/67830753            1326 TGCTCCTGCT~~~~~~ACGAAGTGGAGCAAGGAATATTGTGGGTATTACGAGTGTGGGGTAGTGGTCAGTCCGTTAGAGAAGGTGGAGATTCGGCTGAGC
TpNRabbit/37356380         1320 TGCTCCTGCT~~~~~~ACGAAGTGGAGCAAGGAATATTGTGGGTATTACGAGTGTGGGGTAGTGGTCAGTCCGTTAGAGAAGGTGGAGATTCGGCTGAGC
TpNIVA_d1288/62194580      1320 TGCTCCTGCT~~~~~~ACGAAGTGGAGCAAGGAATATTGTGGGTATTACGAGTGTGGGGTAGTGGTCAGTCCGTTAGAGAAGGTGGAGATTCGGCTGAGC
TpNIVB_d1274/56361402      1320 TGCTCCTGCT~~~~~~ACGAAGTGGAGCAAGGAATATTGTGGGTATTACGAGTGTGGGGTAGTGGTCAGTCCGTTAGAGAAGGTGGAGATTCGGCTGAGC
TpNIVB_d1274/57606550      1335 TGCTCCTGCT~~~~~~ACGAAGTGGAGCAAGGAATATTGTGGGTATTACGAGTGTGGGGTAGTGGTCAGTCCGTTAGAGAAGGTGGAGATTCGGCTGAGC
TpNCL2/71434737            1320 TGCTCCTGCT~~~~~~ACGAAGTGGAGCAAGGAATATTGTGGGTATTACGAGTGTGGGGTAGTGGTCAGTCCGTTAGAGAAGGTGGAGATTCGGCTGAGC
TpNCL5/23396777            1320 TGCTCCTGCT~~~~~~ACGAAGTGGAGCAAGGAATATTGTGGGTATTACGAGTGTGGGGTAGTGGTCAGTCCGTTAGAGAAGGTGGAGATTCGGCTGAGC
TpNCL8/37093850            1320 TGCTCCTGCT~~~~~~ACGAAGTGGAGCAAGGAATATTGTGGGTATTACGAGTGTGGGGTAGTGGTCAGTCCGTTAGAGAAGGTGGAGATTCGGCTGAGC
TpNIVB_d1274/39452938      1320 TGCTCCTGCT~~~~~~ACGAAGTGGAGCAAGGAATATTGTGGGTATTACGAGTGTGGGGTAGTGGTCAGTCCGTTAGAGAAGGTGGAGATTCGGCTGAGC
TpNIVA_d1288/9765540       1320 TGCTCCTGCT~~~~~~ACGAAGTGGAGCAAGGAATATTGTGGGTATTACGAGTGTGGGGTAGTGGTCAGTCCGTTAGAGAAGGTGGAGATTCGGCTGAGC
TpNCL3/13042505            1320 TGCTCCT~~~~~~~~~ACGAAGTGGAAGGCAGGATATTGTGGGTATTACGAGTGTGGGGTAGTGGTCAGTCCGTTAGAGAAGGTGGAGATTCGGCTGAGC
TpNCL3/50332092            1320 TGCTCCT~~~~~~~~~ACGAAGTGGAAGGCAGGATATTGTGGGTATTACGAGTGTGGGGTAGTGGTCAGTCCGTTAGAGAAGGTGGAGATTCGGCTGAGC
TpNCL3/24642061            1323 TGCTCCT~~~~~~~~~ACGAAGTGGAAGGCAGGATATTGTGGGTATTACGAGTGTGGGGTAGTGGTCAGTCCGTTAGAGAAGGTGGAGATTCGGCTGAGC
TpNCL3/25952592            1317 TGCTCCT~~~~~~~~~ACGAAGTGGAAGGCAGGATATTGTGGGTATTACGAGTGTGGGGTAGTGGTCAGTCCGTTAGAGAAGGTGGAGATTCGGCTGAGC
TpNCL3/61080088            1317 TGCTCCT~~~~~~~~~ACGAAGTGGAAGGCAGGATATTGTGGGTATTACGAGTGTGGGGTAGTGGTCAGTCCGTTAGAGAAGGTGGAGATTCGGCTGAGC
TpNCL3/39322209            1320 TGCTCCT~~~~~~~~~ACGAAGTGGAAGGCAGGATATTGTGGGTATTACGAGTGTGGGGTAGTGGTCAGTCCGTTAGAGAAGGTGGAGATTCGGCTGAGC
TpNCL3/73007774            1320 TGCTCCT~~~~~~~~~ACGAAGTGGAAGGCAGGATATTGTGGGTATTACGAGTGTGGGGTAGTGGTCAGTCCGTTAGAGAAGGTGGAGATTCGGCTGAGC
TpNCL3/46924011            1314 TGCTCCT~~~~~~~~~ACGAAGTGGAAGGCAGGATATTGTGGGTATTACGAGTGTGGGGTAGTGGTCAGTCCGTTAGAGAAGGTGGAGATTCGGCTGAGC
TpNCL3/34013629            1317 TGCTCCT~~~~~~~~~ACGAAGTGGAAGGCAGGATATTGTGGGTATTACGAGTGTGGGGTAGTGGTCAGTCCGTTAGAGAAGGTGGAGATTCGGCTGAGC
TpNCL4/16253724            1344 TGCTCCTGCTGCTCCTACGAAGTGGAAGGCAGGATATTGTGGGTATTACGAGTGTGGGGTAGTGGTCAGTCCGTTAGAGAAGGTGGAGATTCGGCTGAGC
TpNCL4/67830320            1344 TGCTCCTGCT~~~~~~ACGAAGTGGAAGGCAGGATATTGTGGGTATTACGAGTGTGGGGTAGTGGTCAGTCCGTTAGAGAAGGTGGAGATTCGGCTGAGC
TpNCL4/49087362            1344 TGCTCCTGCTGCTCCTACGAAGTGGAAGGCAGGATATTGTGGGTATTACGAGTGTGGGGTAGTGGTCAGTCCGTTAGAGAAGGTGGAGATTCGGCTGAGC
TpNCL4/56558172            1344 TGCTCCTGCTGCTCCTACGAAGTGGAAGGCAGAATATTGTGGGTATTACGAGTGTGGGGTAGTGGTCAGTCCGTTAGAGAAGGTGGAGATTCGGCTGAGC
TpNCL4/24117649            1344 TGCTCCTGCTGCTCCTACGAAGTGGAAGGCAGGATATTGTGGGTATTACGAGTGTGGGGTAGTGGTCAGTCCGTTAGAGAAGGTGGAGATTCGGCTGAGC
TpNCL4/74580654            1347 TGCTCCTGCTGCTCCTACGAAGTGGAAGGCAGGATATTGTGGGTATTACGAGTGTGGGGTAGTGGTCAGTCCGTTAGAGAAGGTGGAGATTCGGCTGAGC
TpNCL4/29164148            1347 TGCTCCTGCTCCTGCTACGAAGTGGAAGGCAGAATATTGTGGGTATTACGAGTGTGGGGTAGTGGTCAGTCCGTTAGAGAAGGTGGAGATTCGGCTGAGC
TpNIVA_d1288/44237532      1329 TGCTCCTGCTGCTCCTACGAAGTGGAAGGCAGGATATTGTGGGTATTACGAGTGTGGGGTAGTGGTCAGTCCGTTAGAGAAGGTGGAGATTCGGCTGAGC
TpNIVA_d1288/52757423      1329 TGCTCCTGCTGCTCCTACGAAGTGGAAGGCAGGATATTGTGGGTATTACGAGTGTGGGGTAGTGGTCAGTCCGTTAGAGAAGGTGGAGATTCGGCTGAGC
TpNRabbit/68420554         1344 TGCTCTT~~~~~~~~~ACGAAGTGGGAGGCAGGATATTGTGGGTATTACGAGTGTGGGGTAGTGGTCAGTCCGTTAGAGAAGGTGGAGATTCGGCTGAGC
TpNIVA_d1288/72286947      1348 ~~~~~~~GCTGCTCCTACGAAGTGGAAGGCAGGATATTGTGGGTATTACGAGTGTGGGGTAGTGGTCAGTCCGTTAGAGAAGGTGGAGATTCGGCTGAGC
TpNIVA_d1288/15598261      1327 ~GCTCCTGCT~~~~~~ACGAAGTGGAAGGCAGAATATTGTGGGTATTACGAGTGTGGGGTAGTGGTCAGTCCGTTAGAGAAGGTGGAGATTCGGCTGAGC
```

```
CP004010.2_tprK           1423 TGGGAGCAAGGCAAGCTACAAGAGAACAGCAATGTAGTGATAGAGAAGAACGTGACGGAGCGTTGGCAATTCGTAGGGGCATGTCGCTTGATTTGGTAG
TpNRabbit/11797378        1420 TGGGAGCAAGGCAAGCTACAAGAGAACAGCAATGTAGTGATAGAGAAGAACGTGACGGAGCGTTGGCAATTCGTAGGGGCATGTCGCTTGATTTGGTAG
TpNRabbit/49086983        1420 TGGGAGCAAGGCAAGCTACAAGAGAACAGCAATGTAGTGATAGAGAAGAACGTGACGGAGCGTTGTCAATTCGTAGGGGCATGTCGCTTGATTTGGTAG
TpNCL1/74908282           1420 TGGGAGCAAGGCAAGCTACAAGAGAACAGCAATGTAGTGATAGAGAAGAACGTGACGGAGCGTTGTCAATTCGTAGGGGCATGTCGCTTGATTTGGTAG
TpNRabbit/74646114        1420 TGGGAGCAAGGCAAGCTACAAGAGAACAGCAATGTAGTGATAGAGAAGAACGTGACGGAGCGTTGGCAATTCGTAGGGGCATGTCGCTTGATTTGGTAG
TpNIVA_d1288/58000085     1420 TGGGAGCAAGGCAAGCTACAAGAGAACAGCAATGTAGTGATAGAGAAGAACGTGACGGAGCGTTGTCAATTCGTAGGGGCATGTCGCTTGATTTGGTAG
TpNCL1/67830753           1420 TGGGAGCAAGGCAAGCTACAAGAGAACAGCAATGTAGTGATAGAGAAGAACGTGACGGAGCGTTGTCAATTCGTAGGGGCATGTCGCTTGATTTGGTAG
TpNRabbit/37356380        1414 TGGGAGCAAGGCAAGCTACAAGAGAACAGCAATGTAGTGATAGAGAAGAACGTGACGGAGCGTTGGCAATTCGTAGGGGCATGTCGCTTGATTTGGTAG
TpNIVA_d1288/62194580     1414 TGGGAGCAAGGCAAGCTACAAGAGAACAGCAATGTAGTGATAGAGAAGAACGTGACGGAGCGTTGGCAATTCGTAGGGGCATGTCGCTTGATTTGGTAG
TpNIVB_d1274/56361402     1414 TGGGAGCAAGGCAAGCTACAAGAGAACAGCAATGTAGTGATAGAGAAGAACGTGACGGAGCGTTGGCAATTCGTAGGGGCATGTCGCTTGATTTGGTAG
TpNIVB_d1274/57606550     1429 TGGGAGCAAGGCAAGCTACAAGAGAACAGCAATGTAGTGATAGAGAAGAACGTGACGGAGCGTTGGCAATTCGTAGGGGCATGTCGCTTGATTTGGTAG
TpNCL2/71434737           1414 TGGGAGCAAGGCAAGCTACAAGAGAACAGCAATGTAGTGATAGAGAAGAACGTGACGGAGCGTTGGCAATTCGTAGGGGCATGTCGCTTGATTTGGTAG
TpNCL5/23396777           1414 TGGGAGCAAGGCAAGCTACAAGAGAACAGCAATGTAGTGATAGAGAAGAACGTGACGGAGCGTTGGCAATTCGTAGGGGCATGTCGCTTGATTTGGTAG
TpNCL8/37093850           1414 TGGGAGCAAGGCAAGCTACAAGAGAACAGCAATGTAGTGATAGAGAAGAACGTGACGGAGCGTTGGCAATTCGTAGGGGCATGTCGCTTGATTTGGTAG
TpNIVB_d1274/39452938     1414 TGGGAGCAAGGCAAGCTACAAGAGAACAGCAATGTAGTGATAGAGAAGAACGTGACGGAGCGTTGGCAATTCGTAGGGGCATGTCGCTTGATTTGGTAG
TpNIVA_d1288/9765540      1414 TGGGAGCAAGGCAAGCTACAAGAGAACAGCAATGTAGTGATAGAGAAGAACGTGACGGAGCGTTGGCAATTCGTAGGGGCATGTCGCTTGATTTGGTAG
TpNCL3/13042505           1411 TGGGAGCAAGGCAAGCTACAAGAGAACAGCAATGTAGTGATAGAGAAGAACGTGACGGAGCGTTGGCAATTCGTAGGGGCATGTCGCTTGATTTGGTAG
TpNCL3/50332092           1411 TGGGAGCAAGGCAAGCTACAAGAGAACAGCAATGTAGTGATAGAGAAGAACGTGACGGAGCGTTGGCAATTCGTAGGGGCATGTCGCTTGATTTGGTAG
TpNCL3/24642061           1414 TGGGAGCAAGGCAAGCTACAAGAGAACAGCAATGTAGTGATAGAGAAGAACGTGACGGAGCGTTGGCAATTCGTAGGGGCATGTCGCTTGATTTGGTAG
TpNCL3/25952592           1408 TGGGAGCAAGGCAAGCTACAAGAGAACAGCAATGTAGTGATAGAGAAGAACGTGACGGAGCGTTGGCAATTCGTAGGGGCATGTCGCTTGATTTGGTAG
TpNCL3/61080088           1408 TGGGAGCAAGGCAAGCTACAAGAGAACAGCAATGTAGTGATAGAGAAGAACGTGACGGAGCGTTGGCAATTCGTAGGGGCATGTCGCTTGATTTGGTAG
TpNCL3/39322209           1411 TGGGAGCAAGGCAAGCTACAAGAGAACAGCAATGTAGTGATAGAGAAGAACGTGACGGAGCGTTGGCAATTCGTAGGGGCATGTCGCTTGATTTGGTAG
TpNCL3/73007774           1411 TGGGAGCAAGGCAAGCTACAAGAGAACAGCAATGTAGTGATAGAGAAGAACGTGACGGAGCGTTGGCAATTCGTAGGGGCATGTCGCTTGATTTGGTAG
TpNCL3/46924011           1405 TGGGAGCAAGGCAAGCTACAAGAGAACAGCAATGTAGTGATAGAGAAGAACGTGACGGAGCGTTGGCAATTCGTAGGGGCATGTCGCTTGATTTGGTAG
TpNCL3/34013629           1408 TGGGAGCAAGGCAAGCTACAAGAGAACAGCAATGTAGTGATAGAGAAGAACGTGACGGAGCGTTGGCAATTCGTAGGGGCATGTCGCTTGATTTGGTAG
TpNCL4/16253724           1444 TGGGAGCAAGGCAAGCTACAAGAGAACAGCAATGTAGTGATAGAGAAGAACGTGACGGAGCGTTGGCAATTCGTAGGGGCATGTCGCTTGATTTGGTAG
TpNCL4/67830320           1438 TGGGAGCAAGGCAAGCTACAAGAGAACAGCAATGTAGTGATAGAGAAGAACGTGACGGAGCGTTGGCAATTCGTAGGGGCATGTCGCTTGATTTGGTAG
TpNCL4/49087362           1444 TGGGAGCAAGGCAAGCTACAAGAGAACAGCAATGTAGTGATAGAGAAGAACGTGACGGAGCGTTGGCAATTCGTAGGGGCATGTCGCTTGATTTGGTAG
TpNCL4/56558172           1444 TGGGAGCAAGGCAAGCTACAAGAGAACAGCAATGTAGTGATAGAGAAGAACGTGACGGAGCGTTGGCAATTCGTAGGGGCATGTCGCTTGATTTGGTAG
TpNCL4/24117649           1444 TGGGAG~~~GGCAAGCTACAAGAGAACAGCAATGTAGTGATAGAGAAGAACGTGACGGAGCGTTGGCAATTCGTAGGGGCATGTCGCTTGATTTGGTAG
TpNCL4/74580654           1447 TGGGAGCAAGGCAAGCTACAAGAGAACAGCAATGTAGTGATAGAGAAGAACGTGACGGAGCGTTGGCAATTCGTAGGGGCATGTCGCTTGATTTGGTAG
TpNCL4/29164148           1447 TGGGAGCAAGGCAAGCTACAAGAGAACAGCAATGTAGTGATAGAGAAGAACGTGACGGAGCGTTGGCAATTCGTAGGGGCATGTCGCTTGATTTGGTAG
TpNIVA_d1288/44237532     1429 TGGGAGCAAGGCAAGCTACAAGAGAACAGCAATGTAGTGATAGAGAAGAACGTGACGGAGCGTTGGCAATTCGTAGGGGCATGTCGCTTGATTTGGTAG
TpNIVA_d1288/52757423     1429 TGGGAGCAAGGCAAGCTACAAGAGAACAGCAATGTAGTGATAGAGAAGAACGTGACGGAGCGTTGGCAATTCGTAGGGGCATGTCGCTTGATTTGGTAG
TpNRabbit/68420554        1435 TGGGAGCAAGGCAAGCTACAAGAGAACAGCAATGTAGTGATAGAGAAGAACGTGACGGAGCGTTGGCAATTCGTAGGGGCATGTCGCTTGATTTGGTAG
TpNIVA_d1288/72286947     1441 TGGGAGCAAGGCAAGCTACAAGAGAACAGCAATGTAGTGATAGAGAAGAACGTGACGGAGCGTTGGCAATTCGTAGGGGCATGTCGCTTGATTTGGTAG
TpNIVA_d1288/15598261     1420 TGGGAGCAAGGCAAGCTACAAGAGAACAGCAATGTAGTGATAGAGAAGAACGTGACGGAGCGTTGTCAATTCGTAGGGGCATGTCGCTTGATTTGGTAG
```
