# Supplementary material for: Clonal isolates of Treponema pallidum subsp. pallidum Nichols provide evidence for the occurrence of microevolution during experimental rabbit infection and in vitro culture
Source: PLoS One. 2023 Mar 14;18(3):e0281187. doi: 10.1371/journal.pone.0281187 (PMC10013896; doi:10.1371/journal.pone.0281187)
Supplement: S2 Fig — (PDF) [file pone.0281187.s002.pdf]

Fig. S2. Alignment of the full-length *tprK* amino acid sequences obtained by PacBio sequencing. Each read is labeled with the sample name (e.g. TpNRabbit) and the read identification number; these correspond to the identifiers in Fig. 7. The reference sequence (CP004010.2) is included for comparison. The locations of the variable regions (VR1 through VR7) are indicated in red.

```
                                                                                                       VR1
NC_021490.2_tprK          1 MIDPSATSRYGSPRLVSNGFRHRRKVVYQRVGHRRFSLIFFFVVVLGRSPRLWAQVSFTPDIEGYAELAWGIASXXXXXXX~LKHGFKTTTDFKIVFPIV
TpNRabbit/11797378        1 MIDPSATSRYGSPRLVSNGFRHRRKVVYQRVGHRRFSLIFFFVVVLGRSPRLWAQVSFTPDIEGYAELAWGIASD~~GGAQPLKHGFKTTTDFKIVFPIV
TpNRabbit/49086983        1 MIDPSATSRYGSPRLVSNGFRHRRKVVYQRVGHRRFSLIFFFVVVLGRSPRLWAQVSFTPDIEGYAELAWGIASE~DGSAGNLKHGFKTTTDFKIVFPIV
TpNCL1/74908282           1 MIDPSATSRYGSPRLVSNGFRHRRKVVYQRVGHRRFSLIFFFVVVLGRSPRLWAQVSFTPDIEGYAELAWGIASE~DGSAGNLKHGFKTTTDFKIVFPIV
TpNRabbit/74646114        1 MIDPSATSRYGSPRLVSNGFRHRRKVVYQRVGHRRFSLIFFFVVVLGRSPRLWAQVSFTPDIEGYAELAWGIASE~DGSAGNLKHGFKTTTDFKIVFPIV
TpNIVA_d1288/58000085     1 MIDPSATSRYGSPRLVSNGFRHRRKVVYQRVGHRRFSLIFFFVVVLGRSPRLWAQVSFTPDIEGYAELAWGIASE~DGSAGNLKHGFKTTTDFKIVFPIV
TpNCL1/67830753           1 MIDPSATSRYGSPRLVSNGFRHRRKVVYQRVGHRRFSLIFFFVVVLGRSPRLWAQVSFTPDIEGYAELAWGIASE~DGSAGNLKHGFKTTTDFKIVFPIV
TpNRabbit/37356380        1 MIDPSATSRYGSPRLVSNGFRHRRKVVYQRVGHRRFSLIFFFVVVLGRSPRLWAQVSFTPDIEGYAELAWGIASD~GGA~~~LKHGFKTTTDFKIVFPIV
TpNIVA_d1288/62194580     1 MIDPSATSRYGSPRLVSNGFRHRRKVVYQRVGHRRFSLIFFFVVVLGRSPRLWAQVSFTPDIEGYAELAWGIASD~GGA~~~LKHGFKTTTDFKIVFPIV
TpNIVB_d1274/56361402     1 MIDPSATSRYGSPRLVSNGFRHRRKVVYQRVGHRRFSLIFFFVVVLGRSPRLWAQVSFTPDIEGYAELAWGIASD~GGA~~~LKHGFKTTTDFKIVFPIV
TpNIVB_d1274/57606550     1 MIDPSATSRYGSPRLVSNGFRHRRKVVYQRVGHRRFSLIFFFVVVLGRSPRLWAQVSFTPDIEGYAELAWGIASD~GGA~~~LKHGFKTTTDFKIVFPIV
TpNCL2/71434737           1 MIDPSATSRYGSPRLVSNGFRHRRKVVYQRVGHRRFSLIFFFVVVLGRSPRLWAQVSFTPDIEGYAELAWGIASD~GGA~~~LKHGFKTTTDFKIVFPIV
TpNCL5/23396777           1 MIDPSATSRYGSPRLVSNGFRHRRKVVYQRVGHRRFSLIFFFVVVLGRSPRLWAQVSFTPDIEGYAELAWGIASD~GGA~~~LKHGFKTTTDFKIVFPIV
TpNCL8/37093850           1 MIDPSATSRYGSPRLVSNGFRHRRKVVYQRVGHRRFSLIFFFVVVLGRSPRLWAQVSFTPDIEGYAELAWGIASD~GGA~~~LKHGFKTTTDFKIVFPIV
TpNIVB_d1274/39452938     1 MIDPSATSRYGSPRLVSNGFRHRRKVVYQRVGHRRFSLIFFFVVVLGRSPRLWAQVSFTPDIEGYAELAWGIASD~GGA~~~LKHGFKTTTDFKIVFPIV
TpNIVA_d1288/9765540      1 MIDPSATSRYGSPRLVSNGFRHRRKVVYQRVGHRRFSLIFFFVVVLGRSPRLWAQVSFTPDIEGYAELAWGIASD~GGA~~~LKHGFKTTTDFKIVFPIV
TpNCL3/13042505           1 MIDPSATSRYGSPRLVSNGFRHRRKVVYQRVGHRRFSLIFFFVVVLGRSPRLWAQVSFTPDIEGYAELAWGIAYE~NGGAQPLKHGFKTTTDFKIVFPIV
TpNCL3/50332092           1 MIDPSATSRYGSPRLVSNGFRHRRKVVYQRVGHRRFSLIFFFVVVLGRSPRLWAQVSFTPDIEGYAELAWGIAYE~NGGAQPLKHGFKTTTDFKIVFPIV
TpNCL3/24642061           1 MIDPSATSRYGSPRLVSNGFRHRRKVVYQRVGHRRFSLIFFFVVVLGRSPRLWAQVSFTPDIEGYAELAWGIAYE~NGGAQPLKHGFKTTTDFKIVFPIV
TpNCL3/25952592           1 MIDPSATSRYGSPRLVSNGFRHRRKVVYQRVGHRRFSLIFFFVVVLGRSPRLWAQVSFTPDIEGYAELAWGIAYE~NGGAQPLKHGFKTTTDFKIVFPIV
TpNCL3/61080088           1 MIDPSATSRYGSPRLVSNGFRHRRKVVYQRVGHRRFSLIFFFVVVLGRSPRLWAQVSFTPDIEGYAELAWGIAYE~NGGAQPLKHGFKTTTDFKIVFPIV
TpNCL3/39322209           1 MIDPSATSRYGSPRLVSNGFRHRRKVVYQRVGHRRFSLIFFFVVVLGRSPRLWAQVSFTPDIEGYAELAWGIAYE~NGGAQPLKHGFKTTTDFKIVFPIV
TpNCL3/73007774           1 MIDPSATSRYGSPRLVSNGFRHRRKVVYQRVGHRRFSLIFFFVVVLGRSPRLWAQVSFTPDIEGYAELAWGIASE~NGGAQPLKHGFKTTTDFKIVFPIV
TpNCL3/46924011           1 MIDPSATSRYGSPRLVSNGFRHRRKVVYQRVGHRRFSLIFFFVVVLGRSPRLWAQVSFTPDIEGYAELAWGIAYE~NGGAQPLKHGFKTTTDFKIVFPIV
TpNCL3/34013629           1 MIDPSATSRYGSPRLVSNGFRHRRKVVYQRVGHRRFSLIFFFVVVLGRSPRLWAQVSFTPDIEGYAELAWGIAYE~NGGAQPLKHGFKTTTDFKIVFPIV
TpNCL4/16253724           1 MIDPSATSRYGSPRLVSNGFRHRRKVVYQRVGHRRFSLIFFFVVVLGRSPRLWAQVSFTPDIEGYAELAWGIAYE~NGGAQPLKHGFKTTTDFKIVFPIV
TpNCL4/67830320           1 MIDPSATSRYGSPRLVSNGFRHRRKVVYQRVGHRRFSLIFFFVVVLGRSPRLWAQVSFTPDIEGYAELAWGIAYE~NGGAQPLKHGFKTTTDFKIVFPIV
TpNCL4/49087362           1 MIDPSATSRYGSPRLVSNGFRHRRKVVYQRVGHRRFSLIFFFVVVLGRSPRLWAQVSFTPDIEGYAELAWGIASE~TGGAGALKHGFKTTTDFKIVFPIV
TpNCL4/56558172           1 MIDPSATSRYGSPRLVSNGFRHRRKVVYQRVGHRRFSLIFFFVVVLGRSPRLWAQVSFTPDIEGYAELAWGIAYE~NGGAQPLKHGFKTTTDFKIVFPIV
TpNCL4/24117649           1 MIDPSATSRYGSPRLVSNGFRHRRKVVYQRVGHRRFSLIFFFVVVLGRSPRLWAQVSFTPDIEGYAELAWGIAYE~NGGAQPLKHGFKTTTDFKIVFPIV
TpNCL4/74580654           1 MIDPSATSRYGSPRLVSNGFRHRRKVVYQRVGHRRFSLIFFFVVVLGRSPRLWAQVSFTPDIEGYAELAWGIAYE~NGGAQPLKHGFKTTTDFKIVFPIV
TpNCL4/29164148           1 MIDPSATSRYGSPRLVSNGFRHRRKVVYQRVGHRRFSLIFFFVVVLGRSPRLWAQVSFTPDIEGYAELAWGIAYE~NGGAQPLKHGFKTTTDFKIVFPIV
TpNIVA_d1288/44237532     1 MIDPSATSRYGSPRLVSNGFRHRRKVVYQRVGHRRFSLIFFFVVVLGRSPRLWAQVSFTPDIEGYAELAWGIASE~DGSAGALKHGFKTTTDFKIVFPIV
TpNIVA_d1288/52757423     1 MIDPSATSRYGSPRLVSNGFRHRRKVVYQRVGHRRFSLIFFFVVVLGRSPRLWAQVSFTPDIEGYAELAWGIASE~DGSAGALKHGFKTTTDFKIVFPIV
TpNRabbit/68420554        1 MIDPSATSRYGSPRLVSNGFRHRRKVVYQRVGHRRFSLIFFFVVVLGRSPRLWAQVSFTPDIEGYAELAWGIASEKNGGAQPLKHGFKTTTDFKIVFPIV
TpNIVA_d1288/72286947     1 MIDPSATSRYGSPRLVSNGFRHRRKVVYQRVGHRRFSLIFFFVVVLGRSPRLWAQVSFTPDIEGYAELAWGIASE~TGGAQPLKHGFKTTTDFKIVFPIV
TpNIVA_d1288/15598261     1 MIDPSATSRYGSPRLVSNGFRHRRKVVYQRVGHRRFSLIFFFVVVLGRSPRLWAQVSFTPDIEGYAELAWGIASD~GG~~~AIKHGFKTTTDFKIVFPIV
```

```
NC_021490.2_tprK        100 AKKDFKYRGEGNVYAEINVKALKLSLESNGGAKFDTKGSAKTIEATLHCY~GAYLTIGKNPDFKSTFAVLWEPWTANGDYKSKGDKPVYEPGFEGAGGKL
TpNRabbit/11797378       99 AKKDFKYRGEGNVYAEINVKALKLSLESNGGAKFDTKGSAKTIEATLHCY~GAYLTIGKNPDFKSTFAVLWEPWTANGDYKSKGDKPVYEPGFEGAGGKL
TpNRabbit/49086983      100 AKKDFKYRGEGNVYAEINVKALKLSLESNGGAKFDTKGSAKTIEATLHCY~GAYLTIGKNPDFKSTFAVLWEPWTANGDYKSKGDKPVYEPGFEGAGGKL
TpNCL1/74908282         100 AKKDFKYRGEGNVYAEINVKALKLSLESNGGAKFDTKGSAKTIEATLHCY~GAYLTIGKNPDFKSTFAVLWEPWTANGDYKSKGDKPVYEPGFEGAGGKL
TpNRabbit/74646114      100 AKKDFKYRGEGNVYAEINVKALKLSLESNGGAKFDTKGSAKTIEATLHCY~GAYLTIGKNPDFKSTFAVLWEPWTANGDYKSKGDKPVYEPGFEGAGGKL
TpNIVA_d1288/58000085   100 AKKDFKYRGEGNVYAEINVKALKLSLESNGGAKFDTKGSAKTIEATLHCY~GAYLTIGKNPDFKSTFAVLWEPWTANGDYKSKGDKPVYEPGFEGAGGKL
TpNCL1/67830753         100 AKKDFKYRGEGNVYAEINVKALKLSLESNGGAKFDTKGSAKTIEATLHCY~GAYLTIGKNPDFKSTFAVLWEPWTANGDYKSKGDKPVYEPGFEGAGGKL
TpNRabbit/37356380       97 AKKDFKYRGEGNVYAEINVKALKLSLESNGGAKFDTKGSAKTIEATLHCY~GAYLTIGKNPDFKSTFAVLWEPWTANGDYKSKGDKPVYEPGFEGAGGKL
TpNIVA_d1288/62194580    97 AKKDFKYRGEGNVYAEINVKALKLSLESNGGAKFDTKGSAKTIEATLHCY~GAYLTIGKNPDFKSTFAVLWEPWTANGDYKSKGDKPVYEPGFEGAGGKL
TpNIVB_d1274/56361402    97 AKKDFKYRGEGNVYAEINVKALKLSLESNGGAKFDTKGSAKTIEATLHCY~GAYLTIGKNPDFKSTFAVLWEPWTANGDYKSKGDKPVYEPGFEGAGGKL
TpNIVB_d1274/57606550    97 AKKDFKYRGEGNVYAEINVKALKLSLESNGGAKFDTKGSAKTIEATLHCY~GAYLTIGKNPDFKSTFAVLWEPWTANGDYKSKGDKPVYEPGFEGAGGKL
TpNCL2/71434737          97 AKKDFKYRGEGNVYAEINVKALKLSLESNGGAKFDTKGSAKTIEATLHCY~GAYLTIGKNPDFKSTFAVLWEPWTANGDYKSKGDKPVYEPGFEGAGGKL
TpNCL5/23396777          97 AKKDFKYRGEGNVYAEINVKALKLSLESNGGAKFDTKGSAKTIEATLHCY~GAYLTIGKNPDFKSTFAVLWEPWTANGDYKSKGDKPVYEPGFEGAGGKL
TpNCL8/37093850          97 AKKDFKYRGEGNVYAEINVKALKLSLESNGGAKFDTKGSAKTIEATLHCY~GAYLTIGKNPDFKSTFAVLWEPWTANGDYKSKGDKPVYEPGFEGAGGKL
TpNIVB_d1274/39452938    97 AKKDFKYRGEGNVYAEINVKALKLSLESNGGAKFDTKGSAKTIEATLHCY~GAYLTIGKNPDFKSTFAVLWEPWTANGDYKSKGDKPVYEPGFEGAGGKL
TpNIVA_d1288/9765540     97 AKKDFKYRGEGNVYAEINVKALKLSLESNGGAKFDTKGSAKTIEATLHCY~GAYLTIGKNPDFKSTLAVLWEPWTANGDYKSKGDKPVYEPGFEGAGGKL
TpNCL3/13042505         100 AKKDFKYRGEGNVYAEINVKALKLSLESNGGAKFDTKGSAKTIEATLHCY~GAYLTIGKNPDFKSTFAVLWEPWTANGDYKSKGDKPVYEPGFEGAGGKL
TpNCL3/50332092         100 AKKDFKYRGEGNVYAEINVKALKLSLESNGGAKFDTKGSAKTIEATLHCL~GAYLTIGKNPDFKSTFAVLWEPWTANGDYKSKGDKPVYEPGFEGAGGKL
TpNCL3/24642061         100 AKKDFKYRGEGNVYAEINVKALKLSLESNGGAKFDTKGSAKTIEATLHCYGGAYLTIGKNPDFKSTFAVLWEPWTANGDYKSKGDKPVYEPGFEGAGGKL
TpNCL3/25952592         100 AKKDFKYRGEGNVYAEINVKALKLSLESNGGAKFDTKGSAKTIEATLHCY~GAYLTIGKNPDFKSTFAVLWEPWTANGDYKSKGDKPVYEPGFEGAGGKL
TpNCL3/61080088         100 AKKDFKYRGEGNVYAEINVKALKLSLESNGGAKFDTKGSAKTIEATLHCY~GAYLTIGKNPDFKSTFAVLWEPWTANGDYKSKGDKPVYEPGFEGAGGKL
TpNCL3/39322209         100 AKKDFKYRGEGNVYAEINVKALKLSLESNGGAKFDTKGSAKTIEATLHCY~GAYLTIGKNPDFKSTFAVLWEPWTANGDYKSKGDKPVYEPGFEGAGGKL
TpNCL3/73007774         100 AKKDFKYRGEGNVYAEINVKALKLSLESNGGAKFDTKGSAKTIEATLHCY~GAYLTIGKNPDFKSTFAVLWEPWTANGDYKSKGDKPVYEPGFEGAGGKL
TpNCL3/46924011         100 AKKDFKYRGEGNVYAEINVKALKLSLESNGGAKFDTKGSAKTIEATLHCY~GAYLTIGKNPDFKSTFAVLWEPWTANGDYKSKGDKPVYEPGFEGAGGKL
TpNCL3/34013629         100 AKKDFKYRGEGNVYAEINVKALKLSLESNGGAKFDTKGSAKTIEATLHCY~GAYLTIGKNPDFKSTFAVLWEPWTANGDYKSKGDKPVYEPGFEGAGGKL
TpNCL4/16253724         100 AKKDFKYRGEGNVYAEINVKALKLSLESNGGAKFDTKGSAKTIEATLHCY~GAYLTIGKNPDFKSTFAVLWEPWTANGDYKSKGDKPVYEPGFEGAGGKL
TpNCL4/67830320         100 AKKDFKYRGEGNVYAEINVKALKLSLESNGGAKFDTKGSAKTIEATLHCY~GAYLTIGKNPDFKSTFAVLWEPWTANGDYKSKGDKPVYEPGFEGAGGKL
TpNCL4/49087362         100 AKKDFKYRGEGNVYAEINVKALKLSLESNGGAKFDTKGSAKTIEATLHCY~GAYLTIGKNPDFKSTFAVLWEPWTANGDYKSKGDKPVYEPGFEGAGGKL
TpNCL4/56558172         100 AKKDFKYRGEGNVYAEINVKALKLSLESNGGAKFDTKGSAKTIEATLHCY~GAYLTIGKNPDFKSTFAVLWEPWTANGDYKSKGDKPVYEPGFEGAGGKL
TpNCL4/24117649         100 AKKDFKYRGEGNVYAEINVKALKLSLESNGGAKFDTKGSAKTIEATLHCY~GAYLTIGKNPDFKSTFAVLWEPWTANGDYKSKGDKPVYEPGFEGAGGKL
TpNCL4/74580654         100 AKKDFKYRGEGNVYAEINVKALKLSLESNGGAKFDTKGSAKTIEATLHCY~GAYLTIGKNPDFKSTFAVLWEPWTANGDYKSKGDKPVYEPGFEGAGGKL
TpNCL4/29164148         100 AKKDFKYRGEGNVYAEINVKALKLSLESNGGAKFDTKGSAKTIEATLHCY~GAYLTIGKNPDFKSTFAVLWEPWTANGDYKSKGDKPVYEPGFEGAGGKL
TpNIVA_d1288/44237532   100 AKKDFKYRGEGNVYAEINVKALKLSLESNGGAKFDTKGSAKTIEATLHCY~GAYLTIGKNPDFKSTFAVLWEPWTANGDYKSKGDKPVYEPGFEGAVGKL
TpNIVA_d1288/52757423   100 AKKDFKYRGEGNVYAEINVKALKLSLESNGGAKFDTKGSAKTIEATLHCY~GAYLTIGKNPDFKSTFAVLWEPWTANGDYKSKGDKPVYEPGFEGAGGKL
TpNRabbit/68420554      101 AKKDFKYRGEGNVYAEINVKALKLSLESNGGAKFDTKGSAKTIEATLHCY~GAYLTIGKNPDFKSTFAVLWEPWTANGDYKSKGDKPVYEPGFEGAGGKL
TpNIVA_d1288/72286947   100 AKKDFKYRGEGNVYAEINVKALKLSLESNGGAKFDTKGSAKTIEATLHCY~GAYLTIGKNPDFKSTFAVLWEPWTANGDYKSKGDKPVYEPGFEGAGGKL
TpNIVA_d1288/15598261    97 AKKDFKYRGEGNVYAEINVKALKLSLESNGGAKFDTKGSAKTIEATLHCY~GAYLTIGKNPDFKSTFAVLWEPWTANGDYKSKGDKPVYEPGFEGAGGKL
```

```
                                                                        VR2                                                      VR3
NC_021490.2_tprK           199 GYKQTDIAGTGLTFDIAFKFASNTDWEGKDSKGNVPAGVTPSKYGLGGDILFGWERTREDGVQEYIKVELTGNSTLSSDY~AQA~RALAAGAKVSMKLWG
TpNRabbit/11797378         198 GYKQTDIAGTGLTFDIAFKFASNTDWEGKDSKGNVPAGVTPSKYGLGGDILFGWERTREDGVQEYIKVELTGNSTLSSDY~AQA~RALAAGAKVSMKLWG
TpNRabbit/49086983         199 GYKQTDIAGTGLTFDIAFKFASNTDWEGKDSKGNVPAGVTPSKYGLGGDILFGWERTREDGVQEYIKVELTGNSTLSSDY~AQA~RALAAGAKVSMKLWG
TpNCL1/74908282            199 GYKQTDIAGTGLTFDIAFKFASNTDWEGKDSKGNVPAGVTPSKYGLGGDILFGWERTREDGVQEYIKVELTGNSTLSSDY~AQA~RALAAGAKVSMKLWG
TpNRabbit/74646114         199 GYKQTDIAGTGLTFDIAFKFASNTDWEGKDSKGNVPAGVTPSKYGLGGDILFGWERTREDGVQEYIKVELTGNSTLSSDY~AQA~RALAAGAKVSMKLWG
TpNIVA_d1288/58000085      199 GYKQTDIAGTGLTFDIAFKFASNTDWEGKDSKGNVPAGVTPSKYGLGGDILFGWERTREDGVQEYIKVELTGNSTLSSDY~AQA~RALAAGAKVSMKLWG
TpNCL1/67830753            199 GYKQTDIAGTGLTFDIAFKFASNTDWEGKDSKGNVPAGVTPSKYGLGGDILFGWERTREDGVQEYIKVELTGNSTLSSDY~AQA~RALAAGAKVSMKLWG
TpNRabbit/37356380         196 GYKQTDIAGTGLTFDIAFKFASNTDWEGKDSKGNVPAGVTPSKYGLGGDILFGWERTREDGVQEYIKVELTGNSTLSSDY~AQA~RALAAGAKVSMKLWG
TpNIVA_d1288/62194580      196 GYKQTDIAGTGLTFDIAFKFASNTDWEGKDSKGNVPAGVTPSKYGLGGDILFGWERTREDGVQEYIKVELTGNSTLSSDY~AQA~RALAAGAKVSMKLWG
TpNIVB_d1274/56361402      196 GYKQTDIAGTGLTFDIAFKFASNTDWEGKDSKGNVPAGVTPSKYGLGGDILFGWERTREDGVQEYIKVELTGNSTLSSDY~AQA~RALAAGAKVSMKLWG
TpNIVB_d1274/57606550      196 GYKQTDIAGTGLTFDIAFKFASNTDWEGKDSKGNVPAGVTPSKYGLGGDILFGWERTREDGVQEYIKVELTGNSTLSSDY~AQA~RALAAGAKVSMKLWG
TpNCL2/71434737            196 GYKQTDIAGTGLTFDIAFKFASNTDWEGKDSKGNVPAGVTPSKYGLGGDILFGWERTREDGVQEYIKVELTGNSTLSSDY~AQA~RALAAGAKVSMKLWG
TpNCL5/23396777            196 GYKQTDIAGTGLTFDIAFKFASNTDWEGKDSKGNVPAGVTPSKYGLGGDILFGWERTREDGVQEYIKVELTGNSTLSSDY~AQA~RALAAGAKVSMKLWG
TpNCL8/37093850            196 GYKQTDIAGTGLTFDIAFKFASNTDWEGKDSKGNVPAGVTPSKYGLGGDILFGWERTREDGVQEYIKVELTGNSTLSSDY~AQA~RALAAGAKVSMKLWG
TpNIVB_d1274/39452938      196 GYKQTDIAGTGLTFDIAFKFASNTDWEGKDSKGNVPAGVTPSKYGLGGDILFGWERTREDGVQEYIKVELTGNSHLSSDY~AQA~RALAAGAKVSMKLWG
TpNIVA_d1288/9765540       196 GYKQTDIAGTGLTFDIAFKFASNTDWEGKDSKGNVPAGVTPSKYGLGGDILFGWERTREDGVQEYIKVELTGNSTLSSDY~AQA~RALAAGAKVSMKLWG
TpNCL3/13042505            199 GYKQTDIAGTGLTFDIAFKFASNTDWEGKDSKGNVPAGVTPSKYGLGGDILFGWERTREDGVQEYIKVELTGNSTLSSGY~AQAARALAAGAKVSMKLWG
TpNCL3/50332092            199 GYKQTDIAGTGLTFDIAFKFASNTDWEGKDSKGNVPAGVTPSKYGLGGDILFGWERTREDGVQEYIKVELTGNSTLSSGY~AQAARALAAGAKVSMKLWG
TpNCL3/24642061            200 GYKQTDIAGTGLTFDIAFKFASNTDWEGKDSKGNVPAGVTPSKYGLGGDILFGWERTREDGVQEYIKVELTGNSTLSSGY~AQAARALAAGAKVSMKLWG
TpNCL3/25952592            199 GYKQTDIAGTGLTFDIAFKFASNTDWEGKDSKGNVPAGVTPSKYGLGGDILFGWERTREDGVQEYIKVELTGNSTLSSGY~AQAAPA~AAGAKVSMKLWG
TpNCL3/61080088            199 GYKQTDIAGTGLTFDIAFKFASNTDWEGKDSKGKAPAG~TPSKYGLGGDILFGWERTREDGVQEYIKVELTGNSTLSSGY~AQAARALAAGAKVSMKLWG
TpNCL3/39322209            199 GYKQTDIAGTGLTFDIAFKFASNTDWEGKSNTGAARAGVTPSKYGLGGDILFGWERTREDGVQEYIKVELTGNSTLSSGY~AQAARALAAGAKVSMKLWG
TpNCL3/73007774            199 GYKQTDIAGTGLTFDIAFKFASNTDWEGKDSKGNVPAGVTPSKYGLGGDILFGWERTREDGVQEYIKVELTGNSTLSSGY~AQAARALAAGAKVSMKLWG
TpNCL3/46924011            199 GYKQTDIAGTGLTFDIAFKFASNTDWEGKDSKGNVPAGVTPSKYGLGGDILFGWERTREDGVQEYIKVELTGNSTLSSGY~AQAARALAAGAKVSMKLWG
TpNCL3/34013629            199 GYKQTDIAGTGLTFDIAFKFASNTDWEGKPN~GNVPAGVTPSKYGLGGDILFGWERTREDGVQEYIKVELTGNSTLSSGY~AQAARALAAGAKVSMKLWG
TpNCL4/16253724            199 GYKQTDIAGTGLTFDIAFKFASNTDWEGKDSKGNVPAGVTPSKYGLGGDILFGWERTREDGVQEYIKVELTGNSTLSSGY~AQAARALAAGAKVSMKLWG
TpNCL4/67830320            199 GYKQTDIAGTGLTFDIAFKFASNTDWEGKDSKGNVPAGVTPSKYGLGGDILFGWERTREDGVQEYIKVELTGNSTLSSGY~AQAARALAAGAKVSMKLWG
TpNCL4/49087362            199 GYKQTDIAGTGLTFDIAFKFASNTDWEGKDSKGNVPAGVTPSKYGLGGDILFGWERTREDGVQEYIKVELTGNSTLSSGY~AQAARALAAGAKVSMKLWG
TpNCL4/56558172            199 GYKQTDIAGTGLTFDIAFKFASNTDWEGKDSKGNVPAGVTPSKYGLGGDILFGWERTREDGVQEYIKVELTGNSTLSSGY~AQAARALAAGAKVSMKLWG
TpNCL4/24117649            199 GYKQTDIAGTGLTFDIAFKFASNTDWEGKDSKGNVPAGVTPSKYGLGGDILFGWERTREDGVQEYIKVELTGNSTLSSGY~AQAARALAAGAKVSMKLWG
TpNCL4/74580654            199 GYKQTDIAGTGLTFDIAFKFASNTDWEGKDSKGNVPAGVTPSKYGLGGDILFGWERTREDGVQEYIKVELTGNSTLSSGY~AQAARALAAGAKVSMKLWG
TpNCL4/29164148            199 GYKQTDIAGTGLTFDIAFKFASNTDWEGKDSKGVVQAGVTPSKYGLGGDILFGWERTREDGVQEYIKVELTGNSTLSSGY~AQAARALAAGAKVSMKLWG
TpNIVA_d1288/44237532      199 GYKQTDIAGTGLTFDIAFKFASNTDWEGKDSQGKAPAGVTPSKYGLGGDILFGWERTREDGVQEYIKVELTGNSTLSSGY~AQAARALAAGAKVSMKLWG
TpNIVA_d1288/52757423      199 GYKQTDIAGTGLTFDIAFKFASNTDWEGKDSQGKAPAGVTPSKYGLGGDILFGWERTREDGVQEYIKVELTGNSTLSSGY~AQAARALAAGAKVSMKLWG
TpNRabbit/68420554         200 GYKQTDIAGTGLTFDIAFKFASNTDWEGKDSKGNVHAGVTPSKYGLGGDILFGWERTREDGVQEYIKVELTGNSTLSSGYXAQAARALAAGAKVSMKLWG
TpNIVA_d1288/72286947      199 GYKQTDIAGTGLTFDIAFKFASNTDWEGKDSKGVVQAGVTPSKYGLGGDILFGWERTREDGVQEYIKVELTGNSTLSSDY~~AQARALAAGAKVSMKLWG
TpNIVA_d1288/15598261      196 GYKQTDIAGTGLTFDIAFKFASNTDWEGKDSKGNVPAGVTPSKYGLGGDILFGWERTREDGVQEYIKVELTGNSTLSSGY~~AQAAGAAAGAKVSMKLWG
```

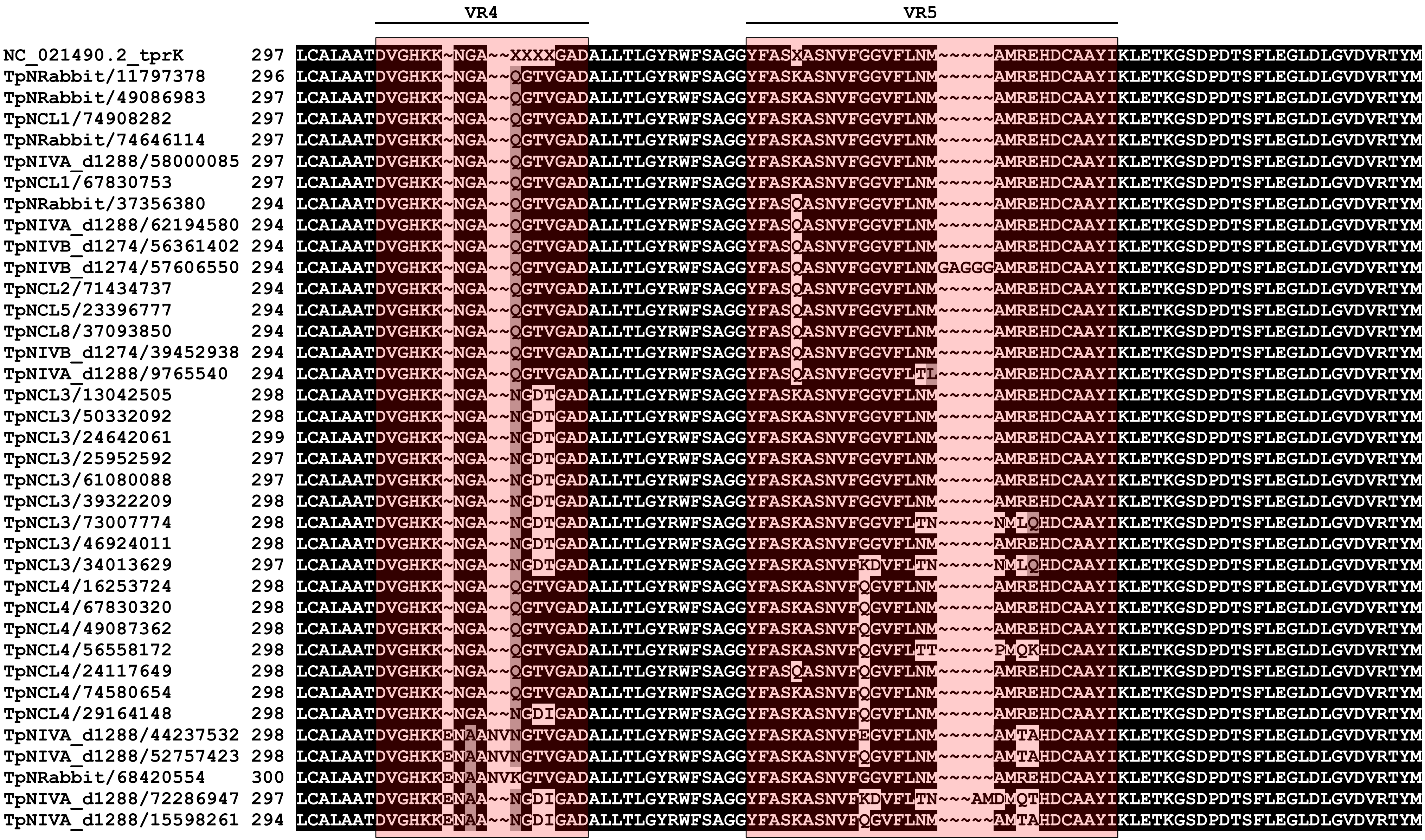
VR4
VR5
NC_021490.2_tprK 297 LCALAATDVGHKK~NGA~~XXXXGADALLTLGYRWFSAGGYFASXASNVFGGVFLNM~~~~~AMREHDCAAYIKLETKGSDPDTSFLEGLDLGVDVRTYM
TpNRabbit/11797378 296 LCALAATDVGHKK~NGA~~QGTVGADALLTLGYRWFSAGGYFASKASNVFGGVFLNM~~~~~AMREHDCAAYIKLETKGSDPDTSFLEGLDLGVDVRTYM
TpNRabbit/49086983 297 LCALAATDVGHKK~NGA~~QGTVGADALLTLGYRWFSAGGYFASKASNVFGGVFLNM~~~~~AMREHDCAAYIKLETKGSDPDTSFLEGLDLGVDVRTYM
TpNCL1/74908282 297 LCALAATDVGHKK~NGA~~QGTVGADALLTLGYRWFSAGGYFASKASNVFGGVFLNM~~~~~AMREHDCAAYIKLETKGSDPDTSFLEGLDLGVDVRTYM
TpNRabbit/74646114 297 LCALAATDVGHKK~NGA~~QGTVGADALLTLGYRWFSAGGYFASKASNVFGGVFLNM~~~~~AMREHDCAAYIKLETKGSDPDTSFLEGLDLGVDVRTYM
TpNIVA_d1288/58000085 297 LCALAATDVGHKK~NGA~~QGTVGADALLTLGYRWFSAGGYFASKASNVFGGVFLNM~~~~~AMREHDCAAYIKLETKGSDPDTSFLEGLDLGVDVRTYM
TpNCL1/67830753 297 LCALAATDVGHKK~NGA~~QGTVGADALLTLGYRWFSAGGYFASKASNVFGGVFLNM~~~~~AMREHDCAAYIKLETKGSDPDTSFLEGLDLGVDVRTYM
TpNRabbit/37356380 294 LCALAATDVGHKK~NGA~~QGTVGADALLTLGYRWFSAGGYFASQASNVFGGVFLNM~~~~~AMREHDCAAYIKLETKGSDPDTSFLEGLDLGVDVRTYM
TpNIVA_d1288/62194580 294 LCALAATDVGHKK~NGA~~QGTVGADALLTLGYRWFSAGGYFASQASNVFGGVFLNM~~~~~AMREHDCAAYIKLETKGSDPDTSFLEGLDLGVDVRTYM
TpNIVB_d1274/56361402 294 LCALAATDVGHKK~NGA~~QGTVGADALLTLGYRWFSAGGYFASQASNVFGGVFLNM~~~~~AMREHDCAAYIKLETKGSDPDTSFLEGLDLGVDVRTYM
TpNIVB_d1274/57606550 294 LCALAATDVGHKK~NGA~~QGTVGADALLTLGYRWFSAGGYFASQASNVFGGVFLNMGAGGGAMREHDCAAYIKLETKGSDPDTSFLEGLDLGVDVRTYM
TpNCL2/71434737 294 LCALAATDVGHKK~NGA~~QGTVGADALLTLGYRWFSAGGYFASQASNVFGGVFLNM~~~~~AMREHDCAAYIKLETKGSDPDTSFLEGLDLGVDVRTYM
TpNCL5/23396777 294 LCALAATDVGHKK~NGA~~QGTVGADALLTLGYRWFSAGGYFASQASNVFGGVFLNM~~~~~AMREHDCAAYIKLETKGSDPDTSFLEGLDLGVDVRTYM
TpNCL8/37093850 294 LCALAATDVGHKK~NGA~~QGTVGADALLTLGYRWFSAGGYFASQASNVFGGVFLNM~~~~~AMREHDCAAYIKLETKGSDPDTSFLEGLDLGVDVRTYM
TpNIVB_d1274/39452938 294 LCALAATDVGHKK~NGA~~QGTVGADALLTLGYRWFSAGGYFASQASNVFGGVFLNM~~~~~AMREHDCAAYIKLETKGSDPDTSFLEGLDLGVDVRTYM
TpNIVA_d1288/9765540 294 LCALAATDVGHKK~NGA~~QGTVGADALLTLGYRWFSAGGYFASQASNVFGGVFLTL~~~~~AMREHDCAAYIKLETKGSDPDTSFLEGLDLGVDVRTYM
TpNCL3/13042505 298 LCALAATDVGHKK~NGA~~NGDTGADALLTLGYRWFSAGGYFASKASNVFGGVFLNM~~~~~AMREHDCAAYIKLETKGSDPDTSFLEGLDLGVDVRTYM
TpNCL3/50332092 298 LCALAATDVGHKK~NGA~~NGDTGADALLTLGYRWFSAGGYFASKASNVFGGVFLNM~~~~~AMREHDCAAYIKLETKGSDPDTSFLEGLDLGVDVRTYM
TpNCL3/24642061 299 LCALAATDVGHKK~NGA~~NGDTGADALLTLGYRWFSAGGYFASKASNVFGGVFLNM~~~~~AMREHDCAAYIKLETKGSDPDTSFLEGLDLGVDVRTYM
TpNCL3/25952592 297 LCALAATDVGHKK~NGA~~NGDTGADALLTLGYRWFSAGGYFASKASNVFGGVFLNM~~~~~AMREHDCAAYIKLETKGSDPDTSFLEGLDLGVDVRTYM
TpNCL3/61080088 297 LCALAATDVGHKK~NGA~~NGDTGADALLTLGYRWFSAGGYFASKASNVFGGVFLNM~~~~~AMREHDCAAYIKLETKGSDPDTSFLEGLDLGVDVRTYM
TpNCL3/39322209 298 LCALAATDVGHKK~NGA~~NGDTGADALLTLGYRWFSAGGYFASKASNVFGGVFLNM~~~~~AMREHDCAAYIKLETKGSDPDTSFLEGLDLGVDVRTYM
TpNCL3/73007774 298 LCALAATDVGHKK~NGA~~NGDTGADALLTLGYRWFSAGGYFASKASNVFGGVFLTN~~~~~NMLQHDCAAYIKLETKGSDPDTSFLEGLDLGVDVRTYM
TpNCL3/46924011 298 LCALAATDVGHKK~NGA~~NGDTGADALLTLGYRWFSAGGYFASKASNVFGGVFLNM~~~~~AMREHDCAAYIKLETKGSDPDTSFLEGLDLGVDVRTYM
TpNCL3/34013629 297 LCALAATDVGHKK~NGA~~NGDTGADALLTLGYRWFSAGGYFASKASNVFKDVFLTN~~~~~NMLQHDCAAYIKLETKGSDPDTSFLEGLDLGVDVRTYM
TpNCL4/16253724 298 LCALAATDVGHKK~NGA~~QGTVGADALLTLGYRWFSAGGYFASKASNVFQGVFLNM~~~~~AMREHDCAAYIKLETKGSDPDTSFLEGLDLGVDVRTYM
TpNCL4/67830320 298 LCALAATDVGHKK~NGA~~QGTVGADALLTLGYRWFSAGGYFASKASNVFQGVFLNM~~~~~AMREHDCAAYIKLETKGSDPDTSFLEGLDLGVDVRTYM
TpNCL4/49087362 298 LCALAATDVGHKK~NGA~~QGTVGADALLTLGYRWFSAGGYFASKASNVFQGVFLNM~~~~~AMREHDCAAYIKLETKGSDPDTSFLEGLDLGVDVRTYM
TpNCL4/56558172 298 LCALAATDVGHKK~NGA~~QGTVGADALLTLGYRWFSAGGYFASKASNVFQGVFLTT~~~~~PMQKHDCAAYIKLETKGSDPDTSFLEGLDLGVDVRTYM
TpNCL4/24117649 298 LCALAATDVGHKK~NGA~~QGTVGADALLTLGYRWFSAGGYFASQASNVFQGVFLNM~~~~~AMREHDCAAYIKLETKGSDPDTSFLEGLDLGVDVRTYM
TpNCL4/74580654 298 LCALAATDVGHKK~NGA~~QGTVGADALLTLGYRWFSAGGYFASKASNVFQGVFLNM~~~~~AMREHDCAAYIKLETKGSDPDTSFLEGLDLGVDVRTYM
TpNCL4/29164148 298 LCALAATDVGHKK~NGA~~NGDIGADALLTLGYRWFSAGGYFASKASNVFQGVFLNM~~~~~AMREHDCAAYIKLETKGSDPDTSFLEGLDLGVDVRTYM
TpNIVA_d1288/44237532 298 LCALAATDVGHKKENAANVNGTVGADALLTLGYRWFSAGGYFASKASNVFEGVFLNM~~~~~AMTAHDCAAYIKLETKGSDPDTSFLEGLDLGVDVRTYM
TpNIVA_d1288/52757423 298 LCALAATDVGHKKENAANVNGTVGADALLTLGYRWFSAGGYFASKASNVFQGVFLNM~~~~~AMTAHDCAAYIKLETKGSDPDTSFLEGLDLGVDVRTYM
TpNRabbit/68420554 300 LCALAATDVGHKKENAANVKGTVGADALLTLGYRWFSAGGYFASKASNVFGGVFLNM~~~~~AMREHDCAAYIKLETKGSDPDTSFLEGLDLGVDVRTYM
TpNIVA_d1288/72286947 297 LCALAATDVGHKKENAA~~NGDIGADALLTLGYRWFSAGGYFASKASNVFKDVFLTN~~~AMDMQTHDCAAYIKLETKGSDPDTSFLEGLDLGVDVRTYM
TpNIVA_d1288/15598261 294 LCALAATDVGHKKENAA~~NGDIGADALLTLGYRWFSAGGYFASKASNVFQGVFLNM~~~~~AMTAHDCAAYIKLETKGSDPDTSFLEGLDLGVDVRTYM

```
                                 VR6                                             VR7
NC_021490.2_tprK          389 PVHYKVLK~~~~~~~ALPPXXXXFPVYGKVWGSYRHDMGEYGWVKVYANLYGGTNKKA~~~~~TPPAAPA~~TKWXXXYCGYYECGVVVSPLEKVEIRLS
TpNRabbit/11797378        388 PVHYKVLK~~~~~~~ALPPA~IYFPVYGKVWGSYRHDMGEYGWVKVYANLYGGTNKKA~~~~~TPPAAPAL~TKWKAEYCGYYECGVVVSPLEKVEIRLS
TpNRabbit/49086983        389 PVHYKVLK~~~~~~~ALPPA~IYFPVYGKVWGSYRHDMGEYGWVKVYANLYGGTNKKA~~~~~TPPAAPA~~TKWKAGYCGYYECGVVVSPLEKVEIRLS
TpNCL1/74908282           389 PVHYKVLK~~~~~~~ALPPA~IYFPVYGKVWGSYRHDMGEYGWVKVYANLYGGTNKKA~~~~~TPPAAPA~~TKWKAGYCGYYECGVVVSPLEKVEIRLS
TpNRabbit/74646114        389 PVHYKVLK~~~~~~~ALPPA~IYFPVYGKVWGSYRHDMGEYGWVKVYANLYGGTNKKA~~~~~TPPAAPA~~TKWSKEYCGYYECGVVVSPLEKVEIRLS
TpNIVA_d1288/58000085     389 PVHYKVLK~~~~~~~ALPPA~IYFPVYGKVWGSYRHDMGEYGWVKVYANLYGGTNKKA~~~~~TPPAAPA~~TKWSKEYCGYYECGVVVSPLEKVEIRLS
TpNCL1/67830753           389 PVHYKVLK~~~~~~~ALPPA~IYFPVYGKVWGSYRHDMGEYGWVKVYANLYGGTNKKA~~~~~TPPAAPA~~TKWSKEYCGYYECGVVVSPLEKVEIRLS
TpNRabbit/37356380        386 PVHYKVLK~~~~~~~ALPPADIHFPVYGKVWGSYRHDMGEYGWVKVYANLYGGTNKKA~~~~~TPPAAPA~~TKWSKEYCGYYECGVVVSPLEKVEIRLS
TpNIVA_d1288/62194580     386 PVHYKVLK~~~~~~~ALPPADIHFPVYGKVWGSYRHDMGEYGWVKVYANLYGGTNKKA~~~~~TPPAAPA~~TKWSKEYCGYYECGVVVSPLEKVEIRLS
TpNIVB_d1274/56361402     386 PVHYKVLK~~~~~~~ALPPADIHFPVYGKVWGSYRHDMGEYGWVKVYANLYGGTNKKA~~~~~TPPAAPA~~TKWSKEYCGYYECGVVVSPLEKVEIRLS
TpNIVB_d1274/57606550     391 PVHYKVLK~~~~~~~ALPPADIHFPVYGKVWGSYRHDMGEYGWVKVYANLYGGTNKKA~~~~~TPPAAPA~~TKWSKEYCGYYECGVVVSPLEKVEIRLS
TpNCL2/71434737           386 PVHYKVLK~~~~~~~ALPPADIHFPVYGKVWGSYRHDMGEYGWVKVYANLYGGTNKKA~~~~~TPPAAPA~~TKWSKEYCGYYECGVVVSPLEKVEIRLS
TpNCL5/23396777           386 PVHYKVLK~~~~~~~ALPPADIHFPVYGKVWGSYRHDMGEYGWVKVYANLYGGTNKKA~~~~~TPPAAPA~~TKWSKEYCGYYECGVVVSPLEKVEIRLS
TpNCL8/37093850           386 PVHYKVLK~~~~~~~ALPPADIHFPVYGKVWGSYRHDMGEYGWVKVYANLYGGTNKKA~~~~~TPPAAPA~~TKWSKEYCGYYECGVVVSPLEKVEIRLS
TpNIVB_d1274/39452938     386 PVHYKVLK~~~~~~~ALPPADIHFPVYGKVWGSYRHDMGEYGWVKVYANLYGGTNKKA~~~~~TPPAAPA~~TKWSKEYCGYYECGVVVSPLEKVEIRLS
TpNIVA_d1288/9765540      386 PVHYKVLK~~~~~~~ALPPADIHFPVYGKVWGSYRHDMGEYGWVKVYANLYGGTNKKA~~~~~TPPAAPA~~TKWSKEYCGYYECGVVVSPLEKVEIRLS
TpNCL3/13042505           390 PVHWK~~~~~~~~~~ALPPA~IYFPVYGKVWGSYRHDMGEYGWVKVYANLYGGTNKKA~~~~~TPPAAP~~~TKWKAGYCGYYECGVVVSPLEKVEIRLS
TpNCL3/50332092           390 PVHWK~~~~~~~~~~ALPPA~IYFPVYGKVWGSYRHDMGEYGWVKVYANLYGGTNKKA~~~~~TPPAAP~~~TKWKAGYCGYYECGVVVSPLEKVEIRLS
TpNCL3/24642061           391 PVHWK~~~~~~~~~~ALPPA~IYFPVYGKVWGSYRHDMGEYGWVKVYANLYGGTNKKA~~~~~TPPAAP~~~TKWKAGYCGYYECGVVVSPLEKVEIRLS
TpNCL3/25952592           389 PVHWK~~~~~~~~~~ALPPA~IYFPVYGKVWGSYRHDMGEYGWVKVYANLYGGTNKKA~~~~~TPPAAP~~~TKWKAGYCGYYECGVVVSPLEKVEIRLS
TpNCL3/61080088           389 PVHWK~~~~~~~~~~ALPPA~IYFPVYGKVWGSYRHDMGEYGWVKVYANLYGGTNKKA~~~~~TPPAAP~~~TKWKAGYCGYYECGVVVSPLEKVEIRLS
TpNCL3/39322209           390 PVHWK~~~~~~~~~~ALPPA~IYFPVYGKVWGSYRHDMGEYGWVKVYANLYGGTNKKA~~~~~TPPAAP~~~TKWKAGYCGYYECGVVVSPLEKVEIRLS
TpNCL3/73007774           390 PVHWK~~~~~~~~~~ALPPA~IYFPVYGKVWGSYRHDMGEYGWVKVYANLYGGTNKKA~~~~~TPPAAP~~~TKWKAGYCGYYECGVVVSPLEKVEIRLS
TpNCL3/46924011           390 PVHWK~~~~~~~~~~ALPPA~IYFPVYGKVWGSYRHDMGEYGWVKVYANLYGGTNKKND~~~~~~~AAP~~~TKWKAGYCGYYECGVVVSPLEKVEIRLS
TpNCL3/34013629           389 PVHWK~~~~~~~~~~ALPPA~IYFPVYGKVWGSYRHDMGEYGWVKVYANLYGGTNKKA~~~~~TPPAAP~~~TKWKAGYCGYYECGVVVSPLEKVEIRLS
TpNCL4/16253724           390 PVHYKVLK~~~~~~~ALPPA~IYFPVYGKVWGSYRHDMGEYGWVKVYANLYGGTNKQAAAVPGVVPAAPAAPTKWKAGYCGYYECGVVVSPLEKVEIRLS
TpNCL4/67830320           390 PVHYKVLK~~~~~~~ALPPA~IYFPVYGKVWGSYRHDMGEYGWVKVYANLYGGTNKQAAAVPGVVPAAPA~~TKWKAGYCGYYECGVVVSPLEKVEIRLS
TpNCL4/49087362           390 PVHYKVLK~~~~~~~ALPPA~IYFPVYGKVWGSYRHDMGEYGWVKVYANLYGGTNKQAAAVPGVVPAAPAAPTKWKAGYCGYYECGVVVSPLEKVEIRLS
TpNCL4/56558172           390 PVHYKVLK~~~~~~~ALPPA~IYFPVYGKVWGSYRHDMGEYGWVKVYANLYGGTNKQAAAVPGVVPAAPAAPTKWKAEYCGYYECGVVVSPLEKVEIRLS
TpNCL4/24117649           390 PVHYKVLK~~~~~~~ALPPA~IYFPVYGKVWGSYRHDMGEYGWVKVYANLYGGTNKQAAAVPGVVPAAPAAPTKWKAGYCGYYECGVVVSPLEKVEIRLS
TpNCL4/74580654           390 PVHWKAPAP~~~~~~AQPPA~INFPVYGKVWGSYRHDMGEYGWVKVYANLYGGTNKQAAAVPGVVPAAPAAPTKWKAGYCGYYECGVVVSPLEKVEIRLS
TpNCL4/29164148           390 PVHWNAFTQ~~~~~~ALPPA~IYFPVYGKVWGSYRHDMGEYGWVKVYANLYGGTNKQAAAVPGVVPAAPAPATKWKAEYCGYYECGVVVSPLEKVEIRLS
TpNIVA_d1288/44237532     393 PVHAQ~~~~~~~~~~APAPA~IYFPVYGKVWGSYRHDMGEYGWVKVYANLYGGTNKKA~~~~~TPPAAPAAPTKWKAGYCGYYECGVVVSPLEKVEIRLS
TpNIVA_d1288/52757423     393 PVHWK~~~~~~~~~~APAPA~IYFPVYGKVWGSYRHDMGEYGWVKVYANLYGGTNKKA~~~~~TPPAAPAAPTKWKAGYCGYYECGVVVSPLEKVEIRLS
TpNRabbit/68420554        395 PVHYKVLK~~~~~~~ALPPA~IYFPVYGKVWGSYRHDMGEYGWVKVYANLYGGTNKKA~~~~~TPPAAL~~~TKWEAGYCGYYECGVVVSPLEKVEIRLS
TpNIVA_d1288/72286947     392 PVHWKAPAQARALPGAPVPA~IYFPVYGKVWGSYRHDMGEYGWVKVYANLYGGTNKKND~~~~~~~~~~AAPTKWKAGYCGYYECGVVVSPLEKVEIRLS
TpNIVA_d1288/15598261     387 PVYYFAAPAAAGAGV~~~~~DINFPVYGKVWGSYRHDMGEYGWVKVYANLYGGTNKKA~~~~~TPP~APA~~TKWKAEYCGYYECGVVVSPLEKVEIRLS
```

NC_021490.2_tprK	475	WEQGKLQENSNVVIEKNVTERWQFVGACRLIW*
TpNRabbit/11797378	474	WEQGKLQENSNVVIEKNVTERWQFVGACRLIW*
TpNRabbit/49086983	474	WEQGKLQENSNVVIEKNVTERCQFVGACRLIW*
TpNCL1/74908282	474	WEQGKLQENSNVVIEKNVTERCQFVGACRLIW*
TpNRabbit/74646114	474	WEQGKLQENSNVVIEKNVTERWQFVGACRLIW*
TpNIVA_d1288/58000085	474	WEQGKLQENSNVVIEKNVTERCQFVGACRLIW*
TpNCL1/67830753	474	WEQGKLQENSNVVIEKNVTERCQFVGACRLIW*
TpNRabbit/37356380	472	WEQGKLQENSNVVIEKNVTERWQFVGACRLIW*
TpNIVA_d1288/62194580	472	WEQGKLQENSNVVIEKNVTERWQFVGACRLIW*
TpNIVB_d1274/56361402	472	WEQGKLQENSNVVIEKNVTERWQFVGACRLIW*
TpNIVB_d1274/57606550	477	WEQGKLQENSNVVIEKNVTERWQFVGACRLIW*
TpNCL2/71434737	472	WEQGKLQENSNVVIEKNVTERWQFVGACRLIW*
TpNCL5/23396777	472	WEQGKLQENSNVVIEKNVTERWQFVGACRLIW*
TpNCL8/37093850	472	WEQGKLQENSNVVIEKNVTERWQFVGACRLIW*
TpNIVB_d1274/39452938	472	WEQGKLQENSNVVIEKNVTERWQFVGACRLIW*
TpNIVA_d1288/9765540	472	WEQGKLQENSNVVIEKNVTERWQFVGACRLIW*
TpNCL3/13042505	471	WEQGKLQENSNVVIEKNVTERWQFVGACRLIW*
TpNCL3/50332092	471	WEQGKLQENSNVVIEKNVTERWQFVGACRLIW*
TpNCL3/24642061	472	WEQGKLQENSNVVIEKNVTERWQFVGACRLIW*
TpNCL3/25952592	470	WEQGKLQENSNVVIEKNVTERWQFVGACRLIW*
TpNCL3/61080088	470	WEQGKLQENSNVVIEKNVTERWQFVGACRLIW*
TpNCL3/39322209	471	WEQGKLQENSNVVIEKNVTERWQFVGACRLIW*
TpNCL3/73007774	471	WEQGKLQENSNVVIEKNVTERWQFVGACRLIW*
TpNCL3/46924011	469	WEQGKLQENSNVVIEKNVTERWQFVGACRLIW*
TpNCL3/34013629	470	WEQGKLQENSNVVIEKNVTERWQFVGACRLIW*
TpNCL4/16253724	482	WEQGKLQENSNVVIEKNVTERWQFVGACRLIW*
TpNCL4/67830320	480	WEQGKLQENSNVVIEKNVTERWQFVGACRLIW*
TpNCL4/49087362	482	WEQGKLQENSNVVIEKNVTERWQFVGACRLIW*
TpNCL4/56558172	482	WEQGKLQENSNVVIEKNVTERWQFVGACRLIW*
TpNCL4/24117649	482	WE~GKLQENSNVVIEKNVTERWQFVGACRLIW*
TpNCL4/74580654	483	WEQGKLQENSNVVIEKNVTERWQFVGACRLIW*
TpNCL4/29164148	483	WEQGKLQENSNVVIEKNVTERWQFVGACRLIW*
TpNIVA_d1288/44237532	477	WEQGKLQENSNVVIEKNVTERWQFVGACRLIW*
TpNIVA_d1288/52757423	477	WEQGKLQENSNVVIEKNVTERWQFVGACRLIW*
TpNRabbit/68420554	479	WEQGKLQENSNVVIEKNVTERWQFVGACRLIW*
TpNIVA_d1288/72286947	481	WEQGKLQENSNVVIEKNVTERWQFVGACRLIW*
TpNIVA_d1288/15598261	474	WEQGKLQENSNVVIEKNVTERCQFVGACRLIW*
