# Supplementary material for: Clonal isolates of Treponema pallidum subsp. pallidum Nichols provide evidence for the occurrence of microevolution during experimental rabbit infection and in vitro culture
Source: PLoS One. 2023 Mar 14;18(3):e0281187. doi: 10.1371/journal.pone.0281187 (PMC10013896; doi:10.1371/journal.pone.0281187)
Supplement: S1 Table — (PDF) [file pone.0281187.s003.pdf]

Table S1. Genomic sequencing information for rabbit-derived samples, in vitro culture-derived samples, and in vitro culture clones of *Treponema pallidum* subsp. *pallidum* Nichols.

**Illumina sequencing sample name**	Source	Genome Size (bp)	Total Number of Reads	Number of Reads Matching *T. pallidum*	Percent Reads Matching *T. pallidum*	Average Read Length	Total Read Length Mapped	Depth of Coverage
TpNRabbit	Rabbit 6L105	1139633	3171238	2898506	91.4	254.0	7.36E+08	646.0
TpNIVA_d137	In vitro culture Tp171020, day 137	1139633	1343914	340636	25.3	255.0	86862180	76.2
TpNIVA_d368	In vitro culture Tp171020, day 368	1139633	1.5E+07	1003061	6.7	142.0	1.42E+08	125.0
TpNIVA_d473	In vitro culture Tp171020, day 473	1139633	2.2E+07	1014281	4.6	141.5	1.44E+08	125.9
TpNIVB_d538	In vitro culture Tp171103, day 538	1139633	2827544	961770	34.0	141.5	1.36E+08	119.4
TpN-CL1	In vitro clone 1	1138497	1.5E+07	1211596	8.1	142.5	1.73E+08	151.6
TpN-CL2	In vitro clone 2	1139537	6443842	1337800	20.8	141.5	1.89E+08	166.1
TpN-CL3	In vitro clone 3	1139665	1.2E+07	1319766	11.2	142.0	1.87E+08	164.4
TpN-CL4	In vitro clone 4	1139647	8930407	1397795	15.7	141.5	1.98E+08	173.6
TpN-CL5	In vitro clone 5	1139582	4462371	999099	22.4	142.5	1.42E+08	124.9
TpN-CL8	In vitro clone 8	1139539	9763203	1144348	11.7	142.5	1.63E+08	143.1

**PacBio CCS sample name**	Source	Genome Size	Total Number of Reads	Number of Reads Matching *T. pallidum*	Percent Reads Matching *T. pallidum*	Average Read Length	Total Read Length Mapped	Depth of Coverage
TpNRabbit	Rabbit 6L105	1139633	12650	985	7.8	5707	5621395	4.9
TpNIVA_d473	In vitro culture Tp171020, day 473	1139633	3359	163	4.9	6609	1077267	0.9
TpNIVA_d1288	In vitro culture Tp171020, day 128	1139633	7180	1097	15.3	5396	5919412	5.2
TpNIVB_d1274	In vitro culture Tp171103, day 127	1139633	6924	1312	18.9	6573	8623776	7.6
TpN-CL1	In vitro clone 1	1138497	7445	1049	14.1	5993	6286657	5.5
TpN-CL2	In vitro clone 2	1139537	16539	2887	17.5	5955	17192085	15.1
TpN-CL3	In vitro clone 3	1139665	23177	2245	9.7	5299	11896255	10.4
TpN-CL4	In vitro clone 4	1139647	9364	1525	16.3	5828	8887700	7.8
TpN-CL5	In vitro clone 5	1139582	18523	769	4.2	5986	4603234	4.0
TpN-CL8	In vitro clone 8	1139539	18868	1987	10.5	5798	11520626	10.1
